# Genetic Variants and Early Cigarette Smoking and Nicotine Dependence Phenotypes in Adolescents

**DOI:** 10.1371/journal.pone.0115716

**Published:** 2014-12-29

**Authors:** Jennifer O'Loughlin, Marie-Pierre Sylvestre, Aurélie Labbe, Nancy C. Low, Marie-Hélène Roy-Gagnon, Erika N. Dugas, Igor Karp, James C. Engert

**Affiliations:** 1 Centre de recherche CHUM, Montreal, Quebec, Canada; 2 Department of Social and Preventive Medicine, University of Montreal, Montreal, Quebec, Canada; 3 Institut national de santé publique du Québec, Montreal, Quebec, Canada; 4 Department of Epidemiology, McGill University, Montreal, Quebec, Canada; 5 Department of Psychiatry, McGill University, Montreal, Quebec, Canada; 6 Department of Epidemiology and Community Medicine, Faculty of Medicine, University of Ottawa, Ottawa, Ontario, Canada; 7 Department of Epidemiology and Biostatistics, Schulich School of Medicine and Dentistry, University of Western Ontario, London, Ontario, Canada; 8 Departments of Medicine and Human Genetics, McGill University, Montreal, Quebec, Canada; 9 Research Institute of the McGill University Health Center, Montreal, Quebec, Canada; Yale University, United States of America

## Abstract

**Background:**

While the heritability of cigarette smoking and nicotine dependence (ND) is well-documented, the contribution of specific genetic variants to specific phenotypes has not been closely examined. The objectives of this study were to test the associations between 321 tagging single-nucleotide polymorphisms (SNPs) that capture common genetic variation in 24 genes, and early smoking and ND phenotypes in novice adolescent smokers, and to assess if genetic predictors differ across these phenotypes.

**Methods:**

In a prospective study of 1294 adolescents aged 12–13 years recruited from ten Montreal-area secondary schools, 544 participants who had smoked at least once during the 7–8 year follow-up provided DNA. 321 single-nucleotide polymorphisms (SNPs) in 24 candidate genes were tested for an association with number of cigarettes smoked in the past 3 months, and with five ND phenotypes (a modified version of the Fagerstrom Tolerance Questionnaire, the ICD-10 and three clusters of ND symptoms representing withdrawal symptoms, use of nicotine for self-medication, and a general ND/craving symptom indicator).

**Results:**

The pattern of SNP-gene associations differed across phenotypes. Sixteen SNPs in seven genes (*ANKK1, CHRNA7, DDC, DRD2, COMT, OPRM1, SLC6A3* (also known as *DAT1*)) were associated with at least one phenotype with a p-value <0.01 using linear mixed models. After permutation and FDR adjustment, none of the associations remained statistically significant, although the p-values for the association between rs557748 in *OPRM1* and the ND/craving and self-medication phenotypes were both 0.076.

**Conclusions:**

Because the genetic predictors differ, specific cigarette smoking and ND phenotypes should be distinguished in genetic studies in adolescents. Fifteen of the 16 top-ranked SNPs identified in this study were from loci involved in dopaminergic pathways (*ANKK1/DRD2, DDC, COMT, OPRM1*, and *SLC6A3*).

**Impact:**

Dopaminergic pathways may be salient during early smoking and the development of ND.

## Introduction

Cigarette smoking remains a major preventable cause of disability and premature death. Nicotine dependence (ND), which involves a complex interplay between learned or conditioned factors, genetics, and social and environmental factors [1], underpins this burden because it causes (prolonged) exposure to carcinogens and the other deleterious constituents in cigarette smoke.

Pharmacologic reasons for nicotine use include mood enhancement, either directly or through relief of withdrawal symptoms, and augmentation of mental or physical function [2]. The psychoactive effects of nicotine are mediated through its influence on the dopamine, serotonin, γ-aminobutyric acid, glutamate, and opioid peptide neurotransmitter systems [3], all of which are involved in the mesocorticolimbic dopamine system and its connections to the nucleus accumbens and amygdala [4] as parts of the dopaminergic reward system [5]. The release of mesolimbic dopamine is triggered by nicotine, which activates striatal neuronal acetylcholine receptors and inhibits its reuptake, thereby increasing levels of synaptic dopamine [6].

Twin and family studies [7], [8] concur that smoking has an important genetic component. The estimated heritability for cigarette smoking initiation is 37% in males and 55% in females. For smoking persistence, it is 59% in males and 46% in females [7]. Sullivan et al. [8] reported a heritability estimate of 70% for tobacco dependence. Because smoking is a complex behavior, it is likely that many genes are involved, each making a small contribution [9].

A ‘reward deficiency syndrome’ has been postulated as one unifying theme to account for the role of diverse neurotransmitters in ND. Consequently many studies have evaluated genes in opioid, serotinergic, dopaminergic, nicotinic, muscarinic and cholinergic receptor genes [10] although candidate gene association studies frequently produce inconsistent results [9], [11], [12]. Recently in a meta-analysis of 15 genome-wide linkage scans, Han et al. [13] identified several chromosomal regions showing evidence of linkage with smoking behavior. Several of these regions contained notable candidate genes including the nicotinic acetylcholine receptor α4 subunit gene (*CHRNA4*) for maximum number of cigarettes smoked in 24 hours, the *PLEKHG1* gene and the μ-opioid receptor gene (*OPRM1*) for ND, and *DRD4* and *COMT* for smoking behavior and maximum number of cigarettes smoked in 24 hours. However, linkage was not detected to regions that contain other candidate genes such as the *ANKK1-DRD2* gene locus and *DDC*.

Genome-wide association studies (GWAS) have also confirmed the role of variants in several nicotinic receptor genes as well as in *CYP2A6*. Three meta-analyses of GWAS, [12], [14], [15] all of which used cigarettes smoked per day as the phenotype, identified genetic variants (i.e., single-nucleotide polymorphisms (SNPs)) in the q25.1 region of chromosome 15, which contains a cluster of nicotinic receptor genes (*CHRNA5-CHRNA3-CHRNB4*). Two of the meta-analyses identified the q13.2 region of chromosome 19 that contains the *EGLN2*/*CYP2A6* locus. One study identified the *CHRNB3/CHRNA6* locus [14], and another observed an association between the *DBH* locus and smoking cessation [12].

The general lack of consistency across studies likely relates to several issues including the assorted phenotypes studied (i.e., cigarettes per day, maximum cigarettes per day, ND, smoking status, initiation, withdrawal, and regular smoking), differing candidate gene coverage, imperfect choice of controls in case control studies, and incomplete control of population heterogeneity [11], [16]. The accurate parsing of broad constructs such as ND which likely meld numerous phenotypes, may help distinguish the unique importance of specific genes and pathways.

While several candidate gene studies have been undertaken in youth, [3], [17], [18] most genetic studies on smoking have been conducted in adults. Accumulating evidence suggests that ND symptoms can occur within months of first puff in some young smokers, [19], [20] challenging older assumptions that the development of ND takes at least two years after smoking onset [21]. Therefore, better understanding of the genetic predictors of levels of cigarette smoking and the development of ND in the early stages of smoking onset is needed. The objectives of this study were to test the associations between 321 tagging single-nucleotide polymorphisms (SNPs) that capture common genetic variation in 24 genes, and early smoking and ND phenotypes in novice adolescent smokers, and to assess if genetic predictors differ across these phenotypes.

## Materials and Methods

The Nicotine Dependence in Teens (NDIT) Study is a prospective cohort investigation of the natural course of ND in adolescents [22]. 1294 students aged 12–13 years were recruited in 1999–2000 in a convenience sample of 10 Montreal-area secondary schools selected to include French and English schools, schools in urban, suburban, and rural areas, and schools in high, moderate and low-income neighbourhoods. Participants were recruited in all grade 7 classes. The baseline response proportion was 55%. Nonparticipation in the NDIT study at baseline primarily related to the need for blood sampling for genetic analysis and to a province-wide labour dispute that resulted in some teachers not collecting consent forms.

Data were collected in classroom-administered self-report questionnaires every three months during the 10-month school year for five years from grade 7 to 11 (1999–2005), for a total of 20 data collection cycles. Blood was collected from 523 participants in 2002. In 2007 participants (mean age 20 years) completed mailed self-report questionnaires in cycle 21, and 336 participants who had not provided blood provided saliva samples in Oragene DNA kits (DNA Genotek Inc. Kanata, Ontario, Canada). In total, 859 participants (66% of 1294) provided either blood or saliva. DNA was extracted using established protocols and genotyped at the McGill University and Genome Quebec Innovation Centre.

### Ethics Statement

Parents/guardians provided informed written consent and participants, who had attained legal age, provided informed written consent in cycle 21. NDIT received institutional approval from the Centre de recherche du Centre Hospitalier de l'Université de Montréal (CRCHUM) (ND06.087).

### Study variables

Data on all six phenotypes were collected in each data collection cycle. Data on number of cigarettes smoked per month were collected in a 3-month recall [23] with two items for each month, one measuring number of days on which the participant had smoked, and one measuring average number of cigarettes smoked per day. Total number of cigarettes smoked in each of the past three months was computed and “number of cigarettes” represented the total number of cigarettes smoked in the past three months. The intraclass correlation coefficient quantifying test-retest reliability of number of cigarettes smoked per month based on the 3-month recall was 0.64 [24].

Table S1 in S1 File details the items that comprise each of the five ND indicators investigated including response options and psychometric properties. Briefly, well-known ND indicators included the modified Fagerstom Tolerance Questionnaire (mFTQ) [25] and an indicator based on the six criteria for tobacco dependence in the International Classification of Diseases 10th Revision (ICD-10) [26]. The other three indicators were ND symptom clusters. In earlier principal components analyses [27], clusters of ND-related symptoms in young smokers were identified from among 46 items drawn from the Hooked on Nicotine Checklist [28], the Stanford Dependence Index [29], and from six focus groups with young smokers [30]. This analysis resulted in the creation of three new scales: an indicator of withdrawal; an indicator of self-medication to alleviate negative affect; and a general ND indicator which included several items that measured cravings referred to herein as a general ND/craving symptom indicator.

### Gene/SNP selection

We included genes from loci that met the genome-wide significance threshold in GWAS studies, including *CHRNA5, CHRNA3, CHRNB4, CHRNB3, CHRNA6, EGLN2* and *DBH* (Table S2 in S1 File). In addition, we selected genes from recent reviews that were reported to be associated with a smoking or ND phenotype in at least two articles and which were involved in neurotransmitter pathways implicated in the development of substance dependence or abuse. These included *BDNF, CHAT, CHRNA4, CHRNB2, COMT, DDC, ANKK1, DRD2, DRD4, MAO-A, OPRM1, SLC6A3, SLC6A4* and *TH*. Finally, we included three other genes (*CHRNA7, GSTM1* and *NR4A2*) of specific interest to the authors, which made biological sense as stated above but for which there was only one article that reported an association. Of the 24 genes investigated, seven (*ANKK1, DBH, DDC, DRD2, DRD4, SLC6A3 (DAT1)*, and *TH*) were in the dopaminergic pathway.

A ‘greedy’ pairwise tagging approach [31], as implemented in the software Haploview [32], was used to tag polymorphisms present in these genes using the International HapMap Project CEU data [33]. We “forced in” 36 candidate SNPs of interest. We captured all SNPs with a minor allele frequency (MAF) >2.5% in the HapMap CEU population but selected tagging SNPs with MAF >5%. The linkage disequilibrium (LD) cut-off was r^2^ = 0.8. We tagged polymorphisms from 10 kb upstream to 10 kb downstream of each gene using genomic positions according to NCBI build 36. SequenomiPlex Gold technology (SequenomInc, San Diego, California) was used for genotyping. The sample and SNP call rate thresholds were 0.95 and 0.80, respectively. Genotype reproducibility was >0.998. A total of 394 SNPs from 24 genes were genotyped. Seventy-three SNPs were excluded (three were monomorphic, three had >20% missing values, 51 had MAF <0.05, and 19 were in Hardy-Weinberg disequilibrium in ≥1 ethnic group). A total of 321 SNPs (Table S2 in S1 File) were retained for analysis.

### Analysis

Of 859 participants with a DNA sample, 544 reported cigarette smoking in at least one data collection cycle. The association between each SNP and each phenotype was investigated with linear mixed models to account for the correlation between repeated measures within participants (i.e., a random intercept and a random linear slope for age were included to account for within-participant correlation). All models also included fixed effects for sex, ethnicity (European, French-Canadian, or Other/Mixed) and age, which was represented by both linear and quadratic terms.

The distribution of number of cigarettes was left-skewed and therefore transformed using the natural logarithm. Each SNP-phenotype association was investigated under an additive genetic model. A total of 21 SNPs (identified in Table S3 in S1 File) with a genotypic frequency ≤15 were recoded into a “dominant model”.

A permutation approach was used to take the dependence structure of the variables used for each of the 1,926 models considered into account, corresponding to the combinations of 321 SNPs from 24 candidate genes and six correlated phenotypes [34]–[35]. Permutation tests are more accurate when variables are correlated [35] as in this analysis, given the moderate to high correlations between phenotypes (Pearson correlation coefficients ranged from 0.47 to 0.83 (Table S4 in S1 File)) and the LD between some SNPs. We thus preserved the structure of the data by permuting individual vectors of 321 genotypes and individual sets of repeated measures of six phenotypes.

Associations with raw p-values <0.01 in the linear mixed models were regarded as statistically significant and multiple testing adjustments were performed using both the false discovery rate (FDR) method [36] and Westfall and Young maxT procedure [34]. Analyses were conducted using R [37] and the lme4 package for linear mixed models [38]. Permutation analyses were conducted on the supercomputer Briarée of the Université de Montréal managed by Calcul Québec and Compute Canada.

## Results

Forty-one percent of participants were male (Table 1). Most participants (76%) were of French-Canadian or other European ancestry. Mean (sd) age at cigarette smoking onset was 14.7 (2.5) years. Participants reported a median (IQR) of 13 (160) cigarettes smoked in the past 3 months.

**Table 1:** Selected Characteristics of NDIT Participants Who Smoked Cigarettes and Had DNA Genotyped.

Characteristic	Total
	(n = 544)
Male, %	41.0
Ethnicity, %	
French Canadian	35.0
Other European (not French-Canadian)	40.6
Other/Mixed*	23.5
Missing	0.9
Age (y) at smoking onset, mean (SD)	14.7 (2.5)
No. cigarettes smoked in past 3 months, median (IQR)	13.0 (160.4)
ND/craving score, median (IQR)	9.3 (13.1)
ICD-10 score, median (IQR)	1.0 (2.0)
mFTQ score, median (IQR)	2.0 (2.4)
Self-medication score, median (IQR)	1.0 (3.0)
Withdrawal score, median (IQR)	2.0 (7.0)

NDIT Study, 1999–2008.

Abbreviations: DNA, deoxyribonucleic acid; ICD-10, International Classification of Disease, 10th Revision;

IQR, interquartile range; mFTQ, Modified Fagerstrom Tolerance Questionnaire; ND, nicotine dependence; NDIT, Nicotine Dependence in Teens; No., number; sd, standard deviation; y, year.

*Includes participants of Chinese, South Asian, Black, Latin American, South-East Asian, Arabic, West Asian, Japanese and mixed ethnicity descent.

The phenotypes investigated were moderately-to-strongly correlated (r = 0.47–0.83) (Table S4 in S1 File). Two SNPs in *COMT* (rs2020917 and rs8140265) were in high LD (r^2^ = 0.98) (Table S5 in S1 File). Similarly, the r^2^ for SNP rs2242592 in *ANKK1* and rs6276 in *DRD2* was 0.98 (hereafter referred to as *DRD2/ANKK1*). Finally two pairs of SNPs in *OPRM1* were in high LD (r^2^ = 0.74 for rs510769 and rs557748, and r^2^ = 0.94 for rs590761 and rs613341).

Table S3 in S1 File shows the estimated regression coefficients for all SNP-phenotype associations investigated controlling for age, sex and ethnicity. While several associations were statistically significant in the linear mixed models, none remained significant after correction for multiple testing according to the significance level obtained from the maxT method (α = 0.000033). None of the FDR corrected permutation-based p-values were statistically significant, but the p-values for the association between rs557748 in *OPRM1* and the ND/craving and self-medication phenotypes were both 0.07632.

Table 2 shows the estimated beta coefficients for all SNP-phenotype associations in which the association was nominally statistically significant (i.e., the p-value before permutation in the linear mixed models <0.01). Sixteen SNPs located in seven genes (*ANKK1, DRD2, CHRNA7, COMT, DDC, OPRM1*, and *SLC6A3*) were associated with at least one phenotype. MAFs for these 16 SNPs were ≥0.08 (Table 3).

**Table 2:** SNPs Associated with Early Smoking and Nicotine Dependence Phenotypes (P-value from Linear Mixed Model <0.01) (n = 544).

Gene	SNP	Phenotype	*β*	p-value from linear mixed model	Permutation-based p-value*	FDR-corrected p-value from linear mixed model	FDR-corrected permutation-based p-value*
*ANKK1*	rs2242592	No. cigarettes	−0.44	0.00594	0.00625	0.48756	0.49807
*DRD2*	rs4586205	No. cigarettes	−0.55	0.00114	0.00129	0.15836	0.17757
	rs4936270	No. cigarettes	−0.87	0.00115	0.00113	0.15836	0.16775
	rs6276	No. cigarettes	−0.44	0.00700	0.00722	0.49934	0.51529
*CHRNA7*	rs868437	mFTQ	−0.30	0.00331	0.00352	0.35432	0.37678
*COMT*	rs174696	Self-medication	−0.36	0.00734	0.00763	0.50491	0.52493
	rs2020917	ND/craving	1.39	0.00681	0.00647	0.49934	0.49807
	rs887200	ND/craving	−2.08	0.00038	0.00045	0.12423	0.12539
		Self-medication	−0.58	0.00031	0.00040	0.12423	0.12539
		mFTQ	−0.42	0.00052	0.00050	0.12423	0.12539
		ICD-10	−0.33	0.00211	0.00204	0.23925	0.23090
	rs8140265	ND/craving	1.41	0.00633	0.00626	0.48756	0.49807
*DDC*	rs4947644	ND/craving	−1.14	0.00977	0.01026	0.52414	0.53402
	rs921451	No. cigarettes	−0.44	0.00568	0.00608	0.48756	0.49807
*OPRM1*	rs510769	ND/craving	1.67	0.00139	0.00151	0.16678	0.18127
		Self-medication	0.50	0.00049	0.00070	0.12423	0.13877
		mFTQ	0.30	0.00626	0.00680	0.48756	0.50408
		ICD-10	0.26	0.00609	0.00642	0.48756	0.49807
		Withdrawal	0.95	0.00076	0.00079	0.13255	0.13877
	rs557748	No. cigarettes	0.64	0.00058	0.00052	0.12423	0.12539
		ND/craving	2.10	0.00005	0.00007	0.05473	0.07632
		Self-medication	0.57	0.00006	0.00008	0.05473	0.07632
		mFTQ	0.35	0.00110	0.00099	0.15836	0.15810
		ICD-10	0.33	0.00050	0.00049	0.12423	0.12539
		Withdrawal	1.05	0.00020	0.00025	0.12423	0.12539
	rs590761	ND/craving	2.29	0.00447	0.00437	0.43006	0.42088
		Self-medication	0.64	0.00369	0.00394	0.37363	0.39941
	rs613341	No. cigarettes	0.75	0.00790	0.00804	0.52414	0.53390
		ND/craving	2.72	0.00067	0.00076	0.12982	0.13877
		Self-medication	0.70	0.00136	0.00151	0.16678	0.18127
		ICD-10	0.38	0.00916	0.00934	0.52414	0.53402
SLC6A3**	rs11737901	Withdrawal	0.68	0.00888	0.00882	0.52414	0.53402

NDIT Study, 1999–2008.

Abbreviations: ICD-10, International Classification of Disease, 10^th^ Revision; mFTQ, Modified Fagerstrom Tolerance Questionnaire; NDIT, Nicotine Dependence In Teens; ND, nicotine dependence; No., number; SNP, single-nucleotide polymorphism.

*Based on 88,326 permutations **Also known as DAT1.

**Table 3:** Biological System, Minor and Major Alleles and Minor Allele Frequency of 16 SNPs from Seven Genes (n = 544). NDIT Study, 1999–2008.

Gene	Biological	SNP	Minor allele	Major allele	MAF*
	System				
*ANKK1*	Ankyrin repeat and kinase domain containing 1) is a protein kinase gene in close physical proximity to DRD2 and contains the DRD2 Taq1A variant	rs2242592	G	A	0.31
*DRD2*	Encodes the D2 subtype of the dopamine receptor	rs4936270	A	G	0.09
		rs4586205	G	A	0.27
		rs6276	C	T	0.31
*CHRNA7*	Nicotinic acetylcholine receptor subunit gene	rs868437	G	A	0.28
*COMT*	Encodes catechol-O methyl transferase, the breakdown enzyme for dopamine, epinephrine, and norepinephrine	rs174696	C	T	0.25
		rs2020917	T	C	0.27
		rs887200	G	A	0.14
		rs8140265	T	C	0.27
*DDC*	Encodes DOPA decarboxylase, a protein that catalyzes essential reactions in the synthesis of dopamine, serotonin and tryptamine	rs4947644	T	C	0.46
		rs921451	C	T	0.33
*OPRM1*	Mu-opioid receptor gene	rs510769	T	C	0.26
		rs557748	T	C	0.23
		rs590761	T	C	0.08
		rs613341	G	A	0.09
*SLC6A3* **	Dopamine transporter gene	rs11737901	A	G	0.37

Abbreviations: NDIT, Nicotine Dependence In Teens; SNP, single-nucleotide polymorphism; MAF, Minor allele frequency.

*Includes French-Canadian and other European ethnicities only.

**Also known as *DAT1*.

Five SNPs from two loci (rs887200 in *COMT*, and rs510769, rs557748, rs590761 and rs613341 in *OPMR1*) were associated with more than one phenotype (Table 4). In *OPRM1*, rs557748 was associated with all six phenotypes and rs510769 was associated with all the phenotypes except number of cigarettes. Number of cigarettes was the only phenotype investigated that was associated with SNPs in *ANKK1/DRD2* and with rs921451 in *DDC*.

**Table 4:** Presence/Absence of Nominally Statistically Significant Associations** Between 16 SNPs from Seven Genes and Early Smoking and Nicotine Dependence Phenotypes (n = 544).

Gene	SNP	Withdrawal	ND/cravings	Self-medication	ICD-10	mFTQ	No. cigarettes
*ANKK1*	rs2242592						x
*DRD2*	rs4936270						x
	rs4586205						x
	rs6276						x
*CHRNA7*	rs868437					x	
*COMT*	rs174696			x			
	rs887200		x	x	x	x	
	rs2020917		x				
	rs8140265		x				
*DDC*	rs4947644		x				
	rs921451						x
*OPRM1*	rs510769	x	x	x	x	x	
	rs557748	x	x	x	x	x	x
	rs590761		x	x			
	rs613341		x	x	x		x
*SLC6A3* *	rs11737901	x					

NDIT Study, 1999–2008.

Abbreviations: ICD-10, International Classification of Diseases. 10th Revision; mFTQ, Modified Fagerstrom Tolerance Questionnaire; NDIT, Nicotine Dependence in Teens; ND, nicotine dependence; No., number; SNP, single-nucleotide polymorphism.

*Also known as *DAT1*.

**p-value from linear mixed models <0.01.

## Discussion

From an analysis of 24 candidate genes in this longitudinal study of adolescents, we identified 16 tagging SNPs at seven genetic loci that were associated with number of cigarettes smoked in the past three months and/or ND phenotypes. Because none of the associations remained statistically significant after correction for multiple testing, the findings in this study must be viewed as preliminary. This discussion focuses on the nominally significant findings.

Despite the relatively high correlation between the phenotypes investigated, the specific pattern of SNP associations differed across phenotypes. In particular, the pattern of SNP-gene associations for number of cigarettes was distinct from the patterns of SNP-gene associations for the ND phenotypes. Number of cigarettes was the only phenotype investigated that was associated with SNPs in *ANKK1/DRD2* and with one SNP in *DDC*. Overall these findings support the tenet that accurate parsing of broad constructs such as smoking and ND may help identify and distinguish the relative importance of specific genes and pathways.

The most notable finding in this study is that five of the seven genes with SNPs associated with the phenotypes in this study (i.e., *ANKK1/DRD2, DDC, CHRNA7*, and *SLC6A3*) are directly involved in the dopaminergic pathway. Further, the opioid pathway was also implicated (i.e., *OPRM1*). This suggests that the rewarding effects of nicotine may be salient during early smoking, and that novice smokers genetically predisposed to experiencing positive dopamine or opioid-related psychoactive effects from nicotine may be susceptible to continuing and/or escalating cigarette consumption and developing ND.

*COMT* is responsible for the breakdown of catecholamines (i.e., dopamine, epinephrine and norepinephrine) [39]. *COMT* Val158Met (rs4680), which has a MAF of 0.50 in European populations [40], causes a valine (high-activity allele) to methionine (low-activity allele) substitution that results in a 3–4-fold difference in *COMT* enzyme activity [41], [42]. In our study, rs4680 was not associated with any of the phenotypes investigated although four other *COMT* SNPs (two of which (rs2020917 and rs8140265) were in high LD) were associated with the ND indicators. In fact, rs887200 was associated with four of the five ND phenotypes, suggesting that *COMT* may underpin the early development of ND in novice smokers. *COMT* findings across studies are generally inconsistent [40], [43], [44].

The candidate gene *DDC* encodes DOPA decarboxylase which catalyzes the decarboxylation of DOPA to dopamine. Ma et al. [45] found that rs921451 was associated with both number of cigarettes smoked per day and the Heaviness of Smoking Index in a European sample of 200 nuclear families (*p* = 0.01–0.04). Further, Yu et al. [46] reported that rs921451 was associated with the Fagerstrom Test for Nicotine Dependence (FTND) score in a sample of 319 African-American and 302 European-American families selected for cocaine or opioid dependence (p = 0.002). In the current study, the *DDC* SNP rs4947644 was associated with one ND phenotype (i.e., ND/cravings), and rs921451 was associated with number of cigarettes.

Although some studies have not detected an association with variation at the *ANKK1/DRD2* locus [47]–[50], multiple studies including two meta-analyses [51], [52] report that *ANKK1/DRD2* is associated with smoking phenotypes [16], [53]–[59] including progression from never to current smoking [3], [17], [60]. In addition, *ANKK1/DRD2* haplotypes have been associated with daily smoking [61] and ND [62], [63]. In our study, one *ANKK1* SNP (rs2242592) and three *DRD2* SNPs (rs4936270, rs4586205 and rs6276) were associated with number of cigarettes smoked in the past three months, but not with any of the ND phenotypes. The *DRD2* SNP rs4586205 is part of the 4-SNP haplotype associated with the Heaviness of Smoking Index in 1266 African-Americans from 402 families [57]. These findings underscore again that number of cigarettes smoked should not be viewed as a proxy measure for ND.

Our study found that rs11737901 in *SLC6A3* (also known as *DAT1* (i.e., dopamine active transporter 1)) was associated with withdrawal. This gene encodes a membrane-spanning protein that mediates the synaptic reuptake of dopamine. *SLC6A3* is the primary regulator of dopamine neurotransmission and is expressed primarily in areas characterized by dopaminergic circuits (striatum and nucleus accumbens). Early studies found an association between *SLC6A3* variants and smoking which several later studies failed to replicate [64], [65]. However in a sample of 668 nicotine-dependent siblings age <18 years in China, the risk of ND (FTND ≥8) was 3-fold higher in those with the *SLC6A3* rs27072-A allele [66]. Bergen et al. [68] reported an association between three SNPs (rs2975226, rs2652510 and rs2652511) and baseline FTND scores in an adult sample of 828 white smokers in two trials of smoking cessation and a pedigree cohort. *SLC6A3* contains a variable number of tandem repeats that affects protein quantity. This polymorphism has two common alleles, a 10-repeat allele and a 9-repeat allele. In a sample of 2,448 young adults from the National Study of Adolescent Health, never smokers and current non-smokers had a higher frequency of the 9-repeat allele, suggesting a protective effect [67]. Stapleton et al. [69], in a meta-analysis of the dopamine transporter genes and smoking cessation, found that four of five cohorts reported a trend in favor of cessation in those carrying the 9-repeat allele. Finally in 2013, Hiemstra et al. [70] did not detect an association between *SLC6A3* genotypes and smoking onset in a longitudinal sample of 365 adolescents.

Mu-opioid receptors are expressed in multiple brain regions and mediate feelings of reward, analgesia and withdrawal. Mu-receptors in the ventral tegmental area are found predominantly on GABAnergic neurons and decrease the level of *GABA* released, which in turn disinhibits dopamine neurons. The opioid receptor is the primary site of action for highly addictive opiates. Non-opiate drugs such as nicotine may also activate mu-opioid receptors by stimulating the release of endogenous endorphins [71]. *OPRM1* (opioid receptor, mu 1) variants have been associated with increased mu-receptor binding in smokers [72] as well as with smoking reward [73]. In our study, the *OPRM1* SNPs rs510769 and rs557748 were in high LD and the results for these two SNPs were very similar. While rs557748 was associated with all phenotypes, rs510769 was associated with five of the six phenotypes investigated. rs510769 was recently reported to be associated with the plasma concentration of cotinine [74]. Our results support the role of this gene across several diverse early smoking phenotypes.

In addition to SNPs from genes involved in dopaminergic pathways, our study also identified an association between rs868437 in the *CHRNA7* nicotinic receptor gene and the mFTQ. In earlier work using NDIT data, we reported that several SNPs located in *CHRN* genes (including rs7178176 in *CHRNA7*) are associated with dizziness at first inhalation, a smoking initiation phenotype that may relate to sustained smoking [75]. There is considerable evidence from both candidate-gene and GWAS studies that nicotinic receptor genes are associated with smoking phenotypes including cigarettes per day [10], [12], [14], [15], [76], serum cotinine [77], withdrawal [78], the Fagerstrom test [79] and early smoking behaviors [80]. However, variation in *CHRNA7* has not been associated with smoking phenotypes as often as other members of the family. One notable exception was confined to African Americans [81]. Several studies used longitudinal designs in population-based samples of youth, but the phenotypes investigated differed from those in our study [17], [18]. Future investigations of nicotinic receptor genes need to differentiate between cigarette smoking and ND phenotypes, at least in novice smokers.

Limitations of this study include the use of self-reported phenotypes which could result in misclassification. However, self-reports of number of cigarettes smoked are generally considered to be valid and reliable in youth, [82] and there are no gold standard measures of ND. Even though we controlled for ethnicity, there may be residual differences in ND phenotypes as well as in LD structure between individuals of French-Canadian, European, and Other/Mixed ancestry. Finally, our study did not detect significant associations between SNPs and the six phenotypes after correction for multiple testing possibly due to a lack of statistical power.

## Conclusions

Genetic predictors appear to differ across early smoking and ND phenotypes supporting the tenet that accurate parsing of broad constructs such as smoking and ND may help distinguish the relative importance of specific genes and pathways in early smoking and the development of ND. Five of the seven genetic loci identified in this study are directly involved in the dopaminergic pathway suggesting that the rewarding psychoactive effects of nicotine may be salient during early smoking in novice smokers.

## Supporting Information

S1 File **This file contains Tables S1–S5.** Table S1, Questionnaire Items, Response Options and Psychometric Properties of Five Nicotine Dependence Phenotypes. NDIT Study, 1999–2008. Table S2, List of 24 Genes and 321 Tag SNPs Tested for an Association with Number of Cigarettes Smoked and Nicotine Dependence Phenotypes, NDIT Study, 1999–2008. Table S3, Estimated Beta Coefficients and *P*-Values for the Associations Between 321 SNPs And Number of Cigarettes Smoked and Nicotine Dependence Phenotypes. (n = 544). NDIT Study, 1999–2008. Table S4, Correlation Coefficients Between Number of Cigarettes Smoked and Nicotine Dependence Phenotypes. NDIT Study (n = 544), 1999–2008. Table S5, Linkage Disequilibrium Correlation Coefficients for SNPs in Genes *COMT, DDC, DRD2/ANKK1* and *OPRM1* that Were Statistically Significantly Associated with the Number of Cigarettes Smoked and/or Nicotine Dependence Phenotypes. NDIT Study, 1999–2008.
(DOCX)
