# Supplementary material for: Genetic Variants and Early Cigarette Smoking and Nicotine Dependence Phenotypes in Adolescents
Source: PLoS One. 2014 Dec 29;9(12):e115716. doi: 10.1371/journal.pone.0115716 (PMC4278712; doi:10.1371/journal.pone.0115716)
Supplement: S1 File — This file contains Tables S1–S5. Table S1, Questionnaire Items, Response Options and Psychometric Properties of Five Nicotine Dependence Phenotypes. NDIT Study, 1999–2008. Table S2, List of 24 Genes and 321 Tag SNPs Tested for an Association with Number of Cigarettes Smoked and Nicotine Dependence Phenotypes, NDIT Study, 1999–2008. Table S3, Estimated Beta Coefficients and P-Values for the Associations Between 321 SNPs And Number of Cigarettes Smoked and Nicotine Dependence Phenotypes. (n = 544). NDIT Study, 1999–2008. Table S4, Correlation Coefficients Between Number of Cigarettes Smoked and Nicotine Dependence Phenotypes. NDIT Study (n = 544), 1999–2008. Table S5, Linkage Disequilibrium Correlation Coefficients for SNPs in Genes COMT, DDC, DRD2/ANKK1 and OPRM1 that Were Statistically Significantly Associated with the Number of Cigarettes Smoked and/or Nicotine Dependence Phenotypes. NDIT Study, 1999–2008. (DOCX) [file pone.0115716.s001.docx]

**Table S1**. Questionnaire Items, Response Options and Psychometric Properties of Five Nicotine Dependence Phenotypes. NDIT Study, 1999-2008.

| Scale | Items | Response options | Psychometric properties |
| --- | --- | --- | --- |
| ICD-10 tobacco dependence - was developed for use in the NDIT Study (1). Comprised 2-4 items for each of its 6 criteria for a total of 18 items. An item was considered positive only if the most extreme response choice was endorsed. A criterion was considered positive if any of its items were positive (the withdrawal syndrome required that ≥2 of 4 items be endorsed). Participants were categorized as tobacco dependent (yes, no) if he/she met ≥3 criteria. | *(1) Strong desire or sense of compulsion to take tobacco* | ― | In NDIT (1), The internal consistency of the ICD-10 was 0.91, test retest reliability was 0.49, and convergent construct validity was demonstrated against quit attempts (2). |
|  | 1. Have you ever had strong cravings to smoke cigarettes? | no, yes |  |
|  | 2. How physically/mentally addicted to smoking are you? | not at all addicted, a little addicted, quite addicted, very addicted |  |
|  | 3. How often have you felt like you really need a cigarette? | never, rarely, sometimes, often |  |
|  | 4. Do you find it difficult not to smoke in places where it’s not allowed (at a movie theatre, at home if your parents don’t know you smoke)? | not at all difficult/I don’t know, a bit difficult, very difficult |  |
|  | *(2) Difficulties controlling tobacco taking behaviour in terms of onset, termination, or level* | ― |  |
|  | 1. In the past 3 months, did you seriously try to quit smoking completely and forever? | yes, I quit completely and have remained non-smoking; I never tried to quit; yes, I tried to quit but failed |  |
|  | 2. Do you smoke cigarettes now because it is really hard to quit? | other/I don’t know/I smoke so little; I don’t know because I have never tried to quit; no; sometimes; often/always |  |
|  | *(3) Physiological withdrawal state when tobacco use has ceased or been reduced, as evidenced by the characteristic withdrawal syndrome for tobacco; or use of the same (or a closely related) substance with the intention of relieving or avoiding withdrawal symptoms* | ― |  |
|  | Now think about the times when you have cut down or stopped using cigarettes or when you haven’t been able to smoke for a long period (like most of the day). How often did you experience the following . . .?  1. Feeling irritable or angry  2. Feeling restless/Feeling nervous, anxious or tense  3. Trouble concentrating  4. Feeling a strong urge or need to smoke | never, rarely, sometimes, often |  |
|  | (*4) Evidence of tolerance, such that increased doses of tobacco are required to achieve effects originally produced by lower doses* | ― |  |
|  | How true are each of the following statements for you?  1. Compared to when I first started smoking, I need to smoke a lot more now to be satisfied. | not at all true, a bit true, very true |  |
|  | 2. Compared to when I first started smoking, I can smoke much more now before I start to feel nauseated or ill. | I’ve never felt nauseated or ill from smoking, not at all true, a bit true, very true |  |
|  | *(5) Progressive neglect of alternative pleasure or interests because of tobacco use, increased amount of time necessary to obtain or take the substance or to recover from its effects* | ― |  |
|  | How true are each of the following statements for you?  1. I spend a lot of time getting cigarettes (going out of my way to a store where I know they will sell to me; trying to find someone who will buy them for me)  2. I’ve stopped hanging out with certain people because of my smoking  3. I avoid going to a friend’s house where you’re not allowed to smoke even though I might enjoy hanging out with him/her  4. I have cut down or stopped physical activity or sports because of my smoking | not at all true, a bit true, very true |  |
|  | *(6) Persisting with tobacco use despite clear evidence of overtly harmful consequences* | ― |  |
|  | How true are each of the following statements for you?  1. In situations where I need to go outside to smoke, it’s worth it even in cold or rainy weather | not at all true, a bit true, very true |  |
|  | 2. If you are sick with a bad cold or sore throat, do you smoke? | no, I don’t have to, I smoke so little; no, I stop smoking when I’m sick; yes, but I cut down on the amount I smoke; yes, I smoke the same amount as when I am sick |  |
| ND/cravings - included 14 items. Responses to all items were summed to create a continuous score which ranged from 0 to 33. | 1. In the past 3 months, did you seriously try to quit smoking completely and forever? | yes, I quit completely and have remained non-smoking ever since; I  never tried to quit; yes, I tried to quit but failed | The internal consistency of the ND/craving symptoms score (α = 0.94) and test retest reliability (ICC = 0.78) are moderate to high, scores increase with increased exposure to tobacco, and they show convergent construct validity against quit attempts (2). |
|  | 2. How often do you have cravings to smoke cigarettes? | never, very rarely, sometimes, often, very often |  |
|  | 3. How physically addicted to smoking cigarettes are you? | not at all, a little addicted, quite addicted, very addicted |  |
|  | 4. How mentally addicted to smoking cigarettes are you? | not at all, a little addicted, quite addicted, very addicted |  |
|  | 5. How often have you felt like you really need a cigarette? | never, rarely, sometimes, often |  |
|  | 6. Do you find it difficult not to smoke in places where it’s not allowed (at a movie theatre, at home if your parents don’t know you smoke)? | not at all/don’t know, a bit difficult, very difficult |  |
|  | 7. If you are sick with a bad cold or sore throat, do you smoke? | no, I don’t have to, I smoke so little; no, I stop smoking when I’m sick; yes, but I cut down on the amount I smoke; yes, I smoke the same amount when I am sick |  |
|  | 8. How deeply do you usually inhale the smoke? | just into my mouth; back into my throat; into my lungs shallow; into my lungs deep |  |
|  | 9. How true is the following statement for you? Cigarettes are good for dealing with boredom | not at all true, a bit true, very true |  |
|  | 10. Do you smoke cigarettes now because it is really hard to quit? | I don’t know/I smoke so little/I quit; no, it is not hard to quit; never tried to quit/I don’t want to quit; yes sometimes, often/always |  |
|  | How true are each of the following statements for you?  11. I often run out of cigarettes quicker than I thought I would  12. I spend a lot of time getting cigarettes (going out of my way to a store where I know they will sell to me; trying to find someone who will buy them for me)  13. I spend a lot of time smoking cigarettes (chain smoking, smoking a lot throughout the day) | not at all true, a bit true, very true |  |
|  | 14. When you see other kids your age smoking cigarettes, how easy is it for you not to smoke? | very easy, quite easy, a bit difficult, very difficult |  |
| Withdrawal - was measured in 6 items with 4-point Likert type response choices, all of which measure withdrawal/compulsion (3). Responses were summed to create a continuous withdrawal score which ranged from 0 to 18 | Think about the times when you have cut down or stopped using cigarettes or when you haven’t been able to smoke for a long period (like most of the day). How often did you experience the following?  1. Feeling irritable or angry  2. Feeling restless  3. Feeling nervous, anxious, or tense  4. Trouble concentrating  5. Feeling a strong urge or need to smoke  6. Trouble sleeping | never, rarely, sometimes, often | The internal consistency of the withdrawal scale (α = 0.88) and test retest reliability (ICC = 0.78) are moderate, and scores on the scale show convergent construct validity against quit attempts and smoking status (2). |
| Self-medication - was measured in 5 items with 3-point Likert type response choices. Responses were summed to create a continuous score which ranged from 0 to 10 | How true are each of the following statements for you?  1. I can function much better in the morning after I’ve had a cigarette  2. When I’m feeling down, a cigarette makes me feel good  3. A cigarette gives me energy when I’m tired  4. Smoking cigarettes helps me concentrate on my homework  5. Smoking cigarettes relieves tension when I am stressed | not at all true, a bit true, very true | The internal consistency (α = 0.85) and test retest reliability (ICC = 0.74) of the self-medication symptom cluster are moderate. Scores increase with increased exposure to tobacco and are associated with quit attempts (1). |
| Modified Fagerström Tolerance Questionnaire (mFTQ) - a 7-item modification of the Fagerstrom Tolerance Questionnaire for adolescents (4). | 1. How many cigarettes a day do you smoke? | <1; 1-15; 16-25; > 25 | Exploratory factor analysis supports a single factor structure (2, 5). The mFTQ has variable internal consistency (α = 0.62–0.90) across studies, and moderate test retest reliability (ICC = 0.76–0.79) (3, 6). Compared to other measures, the mFTQ items show poorer discrimination at lower levels of ND (2). The mFTQ is associated with smoking frequency and predicted cessation at 6-, but not 12-month follow-up (3,, 6). It is also associated with self-reported smoking and saliva cotinine levels (7). |
|  | 2. Do you inhale? | never; seldom; quite often; always |  |
|  | 3. How soon after you wake up do you smoke your first cigarette? | <30 minutes; >30 minutes but before noon; in the afternoon; in the evening |  |
|  | 4. Which cigarette would you hate to give up? | first cigarette in the morning; any other cigarette before noon; any other cigarette in the afternoon; any other cigarette in the evening |  |
|  | 5. Do you find it difficult to refrain from smoking in places where it is forbidden? | yes, very difficult; yes, somewhat difficult; no, not usually difficult; no, not at all difficult |  |
|  | 6. Do you smoke if you are so ill that you are in bed most of the day? | Yes, always; yes, quite often; no, not usually; no, never |  |

**References**

1. [O'Loughlin J](http://www.ncbi.nlm.nih.gov/pubmed?term=O'Loughlin%20J%5BAuthor%5D&cauthor=true&cauthor_uid=12432161), [DiFranza J](http://www.ncbi.nlm.nih.gov/pubmed?term=DiFranza%20J%5BAuthor%5D&cauthor=true&cauthor_uid=12432161), [Tarasuk J](http://www.ncbi.nlm.nih.gov/pubmed?term=Tarasuk%20J%5BAuthor%5D&cauthor=true&cauthor_uid=12432161), [Meshefedjian G](http://www.ncbi.nlm.nih.gov/pubmed?term=Meshefedjian%20G%5BAuthor%5D&cauthor=true&cauthor_uid=12432161), [McMillan-Davey E](http://www.ncbi.nlm.nih.gov/pubmed?term=McMillan-Davey%20E%5BAuthor%5D&cauthor=true&cauthor_uid=12432161), et al. (2002) Assessment of nicotine dependence symptoms in adolescents: a comparison of five indicators. Tob Control 11: 354-360.
2. Wellman RJ, DiFranza JR, Pbert L, Fletcher KE, Flint A, et al. (2006) A comparison of the psychometric properties of the hooked on nicotine checklist and the modified Fagerström tolerance questionnaire. Addict Behav 31: 486–495.
3. O'Loughlin J, Tarasuk J, Difranza J, Paradis G (2002) [Reliability of selected measures of nicotine dependence among adolescents.](http://www.ncbi.nlm.nih.gov/pubmed/12062924) Ann Epidemiol 12: 353-362.
4. [Heatherton TF](http://www.ncbi.nlm.nih.gov/pubmed?term=Heatherton%20TF%5BAuthor%5D&cauthor=true&cauthor_uid=1932883), [Kozlowski LT](http://www.ncbi.nlm.nih.gov/pubmed?term=Kozlowski%20LT%5BAuthor%5D&cauthor=true&cauthor_uid=1932883), [Frecker RC](http://www.ncbi.nlm.nih.gov/pubmed?term=Frecker%20RC%5BAuthor%5D&cauthor=true&cauthor_uid=1932883), [Fagerström KO](http://www.ncbi.nlm.nih.gov/pubmed?term=Fagerstr%C3%B6m%20KO%5BAuthor%5D&cauthor=true&cauthor_uid=1932883) (1991) The Fagerström Test for Nicotine Dependence: a revision of the Fagerström Tolerance Questionnaire. Br J Addict 86: 1119–1127.
5. Strong DR, Schonbrun YC, Schaffran C, Griesler PC, Kandel D (2009) [Linking measures of adolescent nicotine dependence to a common latent continuum.](http://www.ncbi.nlm.nih.gov/pubmed/18938047)Drug Alcohol Depend 99: 296–308.
6. Johnson JL, Ratner PA, Tucker RS, Bottorff JL, Zumbo B, et al. (2005) Development of a multidimensional measure of tobacco dependence in adolescence. [Addict Behav](http://www.ncbi.nlm.nih.gov/pubmed?term=Development%20of%20a%20multidimensional%20measure%20of%20tobacco%20dependence%20in%20adolescence##) 30: 501-515.
7. Chen X, Zheng H, Steve S, Gong J, Stacy A, et al. (2002) Use of the fagerstrom tolerance questionnaire for measuring nicotine dependence among adolescent smokers in China: a pilot test. [Psychol Addict Behav](http://www.ncbi.nlm.nih.gov/pubmed?term=Use%20of%20the%20Fagerstrom%20tolerance%20questionnaire%20for%20measuring%20nicotine%20dependence%20among%20adolescent%20smokers%20in%20China%3A%20a%20pilot%20test##) 16: 260-263.

**Table S2**. List of 24 Genes and 321 Tag SNPs Tested for an Association with Number of Cigarettes Smoked and Nicotine Dependence Phenotypes, NDIT Study, 1999-2008.

| Gene (chromosome) | Function | Tag SNPs |
| --- | --- | --- |
| *1. ANKK1* (11)^1-5^ | Ankyrin repeat and kinase domain containing 1 is a protein kinase gene in close physical proximity to *DRD2* and contains the *DRD2 Taq1A* variant | rs10891545, rs12422191, rs2734848, rs877138, rs11604671, rs11214601, rs1800497, rs2734849, rs2242592, rs754672 (n=10) |
| *2. BDNF* (11)^1,2,5-7^ | The protein encoded by this gene is a member of the nerve growth factor family; gene may play a role in the regulation of stress response and in the biology of mood disorders | rs1013402, rs11030123, rs2049048, rs6265, rs10767653, rs10742179, rs12807253, rs2203877, rs7927728, rs10835201, rs2030324, rs1491851, rs4074134, rs4923456  (n=14) |
| *3. CHAT* (10)^8,25^ | Encodes the enzyme which catalyzes the biosynthesis of the neurotransmitter acetylcholine | rs10857520, rs12266458, rs3793790, rs733722, rs11101204, rs11101179, rs1917810, rs3793791, rs7903496f, rs11591558, rs11101182, rs1917818, rs3810950, rs8178980, rs12217567, rs11101200, rs2177370, rs3810951, rs8178990, rs12246528, rs2889759, rs2377877, rs4838392, rs868750, rs885834, rs3729496, rs3750752, rs7067651, rs7076926, rs7091005 (n=30) |
| *4. CHRNA3* (15) ^2,5,6,9-14^ | Nicotinic acetylcholine receptor subunit gene | rs1051730, rs578776, rs3743077, rs4887069 (n=4) |
| *5. CHRNA4* (20*)* ^1-5,12,15^ | Nicotinic acetylcholine receptor subunit gene | rs1044396, rs9680065, rs2093107, rs6011747, rs755204, rs3787138, rs735501, rs4809538, rs755203, rs4522666 (n=10) |
| *6. CHRNA5* (15) ^2,5,10-14^ | Nicotinic acetylcholine receptor subunit gene | rs11637635, rs16969968, rs569207 (n=3) |
| *7. CHRNA6* (8) ^2,5,12,16^ | Nicotinic acetylcholine receptor subunit gene | rs16891604, rs17621710, rs2304297, rs7812298, rs7828365 (n=5) |
| *8. CHRNA7* (15)^1-3,5,12^ | Nicotinic acetylcholine receptor subunit gene | rs10438342, rs11071530, rs12904458, rs16956223, rs2337980, rs11071512, rs11852956, rs12906868, rs17604861, rs2611603, rs2611605, rs6494212, rs7178176, rs868437, rs4779565, rs7175581, rs8028396, rs885071 (n=18) |
| *9. CHRNB2* (1)^1-5,12,15^ | Nicotinic acetylcholine receptor subunit gene | rs1127309, rs2072660, rs4845652,rs9427094, rs9616, rs2072659, rs3811450, rs9427092 (n=8) |
| *10. CHRNB3* (8)^1-5,9,12,16^ | Nicotinic acetylcholine receptor subunit gene | rs6987323 (n=1) |
| *11. CHRNB4* (15)^2,5,11-14^ | Nicotinic acetylcholine receptor subunit gene | rs11636605, rs17487223, rs1948, rs6495309, rs950776 (n=5) |
| *12. COMT* (22)^1-5,17^ | Encodes catechol-O methyl transferase, the breakdown enzyme for dopamine, epinephrine, and norepinephrine | rs165728, rs174696, rs4646316, rs740603, rs9605030, rs165774, rs2020917, rs4680, rs887200, rs9617850, rs165824, rs2239395, rs5746848, rs9265, rs9332377, rs16982844, rs4646310, rs740601, rs9306235, rs8140265 (n=20) |
| *13. DBH* (9)^1,2,4-6,18^ | Encodes dopamine beta hydroxylase which converts dopamine to noradrenaline in nerve terminals | rs1029372, rs1541332, rs2519143, rs3025382, rs2283124, rs1076150, rs1611114, rs2519148, rs3025388, rs251914, rs1076153, rs1611115, rs2519154, rs3025411, rs3025369, rs10993874, rs1611120, rs2797849, rs5320, rs3025373, rs10993947, rs1611124, rs2797853, rs6271, rs7872903, rs1108580, rs17150735, rs2797855, rs6479643, rs7848964, rs1108581, rs2007153, rs3025343, rs732833, rs3025365, rs129882, rs2073837, rs3025355, rs77905, rs2097628, rs13306304, rs2283123 (n=42) |
| *14.DDC* (7)^1-3,5,19^ | Encodes DOPA decarboxylase, a protein that catalyzes essential reactions in the synthesis of dopamine, serotonin and tryptamine | rs10499696, rs11575464, rs17133877, rs4947644, rs11980368, rs11238133, rs11575500, rs2044859, rs6592963, rs3779078, rs11238134, rs11575548, rs2060762, rs6593011, rs4947510, rs11575282, rs11575553, rs2167364, rs6951648, rs921451, rs11575383, rs11575575, rs3735273, rs880028, rs978784 (n=25) |
| *15. DRD2* (11)^1-5,12^ | Encodes the D2 subtype of the dopamine receptor | rs1079597, rs4245146, rs7131056, rs17529477, rs4936270, rs1107162, rs4586205, rs1799978, rs4938019, rs11214606, rs4630328, rs2440390, rs6276, rs7125415 (n=14) |
| *16. DRD4* (11)^1-4,18^ | Encodes the D4 subtype of the dopamine receptor | rs11246228, rs3758653, rs4331145, rs752306 (n=4) |
| *17. EGLN2* (19)^6^ | Involved in oxygen homeostasis | rs10403040, rs11083568, rs11881124, rs3733829, rs7937, rs10405596, rs11666504, rs2644916, rs4803372 (n=9) |
| *18. GSTM1* (1)^20^ | Detoxification of a wide variety of compounds, such as carcinogens, therapeutic drugs, environmental toxins | rs534314 (n=1) |
| *19. MAO-A* (X)^1-3,5,18,21^ | Encodes monoamine oxidase A, an enzyme that catalyzes the breakdown of dopamine, norepinephrine and serotonin | rs1137070, rs12843533, rs3027415, rs5906957, rs6609257, rs6610845 (n=6) |
| *20. NR4A2* (2)^2^ | Encodes a member of the steroid-thyroid hormone-retinoid receptor superfamily | rs7564993, rs834835 (n=2) |
| *21. OPRM1* (6)^1,2,4,5,22^ | Mu-opiod receptor gene | rs10223804, rs10485060, rs11965988, rs12527056, rs13203628, rs10485058, rs11155954, rs12199124, rs13193545, rs17214592, rs17277929, rs1852629, rs2103277, rs4626436, rs4870266, rs17278409, rs1950005, rs3798683, rs483481, rs510587, rs17292684, rs2010884, rs4295492, rs4869817, rs510769, rs518596, rs590761, rs6557337, rs790258, rs9397687, rs557748, rs610231, rs6557339, rs9322446, rs9478527, rs563649, rs613341, rs6913456, rs9322451, rs9479769, rs581439, rs633387, rs6938037, rs9371331, rs9479771, rs9479791, rs655059, rs7745095, rs9383697, rs9479780 (n=50) |
| *22. SCL6A3 (also known as DAT1)* (5)^1,2,4,5,23^ | Dopamine transporter gene | rs10064525, rs1048953, rs11564772, rs12516758, rs2042449, rs1042098, rs11564757, rs11737901, rs12652860, rs2455391, rs250681, rs27048, rs40184, rs6876225, rs3756450, rs2550948, rs27072, rs40358, rs6347, rs3776511, rs2617605, rs27074, rs456082 (n=23) |
| *23. SCL6A4* (17)^5,24^ | Serotonin transporter gene | rs11080121, rs2020942, rs4251417, rs140700, rs7214991, rs25528 (n=6) |
| *24. TH* (11)^2-5^ | Encodes tyrosine hydroxylase, the enzyme relate to the synthesis of dopamine, epinephrine and epinephrine | rs10743149, rs11564709, rs3842752, rs6578993, rs6356, rs10840447, rs2070762, rs4930046, rs7119275, rs3842727, rs10840491 (n=11) |

**References**

1. National Cancer Institute (2009) Phenotypes and Endophenotypes: Foundations for Genetic Studies of Nicotine Use and Dependence. Tobacco Control Monograph No. 20. Bethesda: U.S. Department of Health and Human Services, National Institutes of Health, National Cancer Institute. NIH Publication No. 09-6366.
2. Conti DV, Lee W, Li D, Liu J, Van Den Berg D, et al. (2008) Pharmacogenetics of Nicotine Addiction and Treatment Consortium. Nicotinic acetylcholine receptor beta2 subunit gene implicated in a systems-based candidate gene study of smoking cessation. Hum Mol Genet 17: 2834-2848.
3. Munafò M, Johnstone EC (2008) Genes and cigarette smoking. Addiction 103:893–904.
4. Schnoll RA, Johnson TA, Lerman C (2007) Genetics and smoking behavior. Curr Psychiatry Rep 9: 349-357.
5. Saccone SF, Hinrichs AL, Saccone NL, Chase GA, Konvicka K, et al. (2007) Cholinergic nicotinic receptor genes implicated in a nicotine dependence association study targeting 348 candidate genes with 3713 SNPs. Hum Mol Genet 16: 36-49.
6. Tobacco and Genetics Consortium (2010) Genome-wide meta-analyses identify multiple loci associated with smoking behavior. Nat Genet 42: 441-447.
7. Vink JM, Smit AB, Geus EJ, Sullivan P, Willemsen G, et al. (2009) Genome-wide association study of smoking initiation and current smoking. Am J Hum Genet 84: 367-379.
8. Ray R, Mitra N, Baldwin D, Guo M, Patterson F, et al. (2010) Convergent evidence that choline acetyltransferase gene variation is associated with prospective smoking cessation and nicotine dependence. Neuropsychopharmacology 35: 1374-1382.
9. Bierut LJ, Madden PA, Breslau N, Johnson EO, Hatsukami D, et al. (2007) Novel genes identified in a high-density genome wide association study for nicotine dependence. Hum Mol Genet 16: 24-35.
10. Berrettini W, Yuan X, Tozzi F, Song K, Francks C, et al. (2008) Alpha-5/alpha-3 nicotinic receptor subunit alleles increase risk for heavy smoking. Mol Psychiatry 13: 368-373.
11. Thorgeirsson TE, Geller F, Sulem P, Rafnar T, Wiste A, et al. (2008) A variant associated with nicotine dependence, lung cancer and peripheral arterial disease. Nature 452: 638-642.
12. Li MD, Ma JZ, Beuten J (2004) Progress in searching for susceptibility loci and genes for smoking-related behaviour. Clin Genet 66: 382-392.
13. Bierut LJ, Stitzel JA, Wang JC, Hinrichs AL, Grucza RA, et al. (2008) Variants in nicotinic receptors and risk for nicotine dependence. Am J Psychiatry 165: 1163-1171.
14. Weiss RB, Baker TB, Cannon DS, von Niederhausern A, Dunn DM, et al. (2008) A candidate gene approach identifies the CHRNA5-A3-B4 region as a risk factor for age-dependent nicotine addiction. PLoS Genet 4: e1000125.
15. Feng Y, Niu T, Xing H, Xu X, Chen C, et al. (2004) A common haplotype of the nicotine acetylcholine receptor alpha 4 subunit gene is associated with vulnerability to nicotine addiction in men. Am J Hum Genet 75: 112-121.
16. Hoft NR, Corley RP, McQueen MB, Schlaepfer IR, Huizinga D, et al. (2009) Genetic association of the CHRNA6 and CHRNB3 genes with tobacco dependence in a nationally representative sample. Neuropsychopharmacology 34: 698-706.
17. Omidvar M, Stolk L, Uitterlinden AG, Hofman A, Van Duijn CM, et al. (2009) The effect of catechol-O-methyltransferase Met/Val functional polymorphism on smoking cessation: retrospective and prospective analyses in a cohort study. Pharmacogenet Genomics 19: 45-51.
18. Huang S, Cook DG, Hinks LJ, Chen XH, Ye S, et al. (2005) CYP2A6, MAOA, DBH, DRD4, and 5HT2A genotypes, smoking behaviour and cotinine levels in 1518 UK adolescents. Pharmacogenet Genomics15: 839-850.
19. Ma JZ, Beuten J, Payne TJ, Dupont RT, Elston RC, et al. (2005) Haplotype analysis indicates an association between the DOPA decarboxylase (DDC) gene and nicotine dependence. Hum Mol Genet 14: 1691-1698.
20. Odebrecht Vargas Nunes S, Pizzo de Castro MR, Ehara Watanabe MA, Losi Guembarovski R, Odebrecht Vargas H, et al. (2014) Genetic polymorphisms in glutathione-S-transferases are associated with anxiety and mood disorders in nicotine dependence. Psychiatr Genet 24:87-93.
21. Caporaso N, Gu F, Chatterjee N, Sheng-Chih J, Yu K, et al. (2009) Genome-wide and candidate gene association study of cigarette smoking behaviors. PloS One 4: e4653.
22. Zhang L, Kendler KS, Chen X (2006) The mu-opioid receptor gene and smoking initiation and nicotine dependence. Behav Brain Funct 2: 28.
23. Ling D, Niu T, Feng Y, Xing H, Xu X (2004) Association between polymorphism of the dopamine transporter gene and early smoking onset: an interaction risk on nicotine dependence. J Hum Genet 49: 35-39.
24. Iordanidou M, Tavridou A, Petridis I, Kyroglou S, Kaklamanis L, et al. (2010) Association of polymorphisms of the serotonergic system with smoking initiation in Caucasians. Drug Alcohol Depend 108: 70-76.
25. [Wei J](http://www.ncbi.nlm.nih.gov/pubmed?term=Wei%20J%5BAuthor%5D&cauthor=true&cauthor_uid=20383528), [Ma JZ](http://www.ncbi.nlm.nih.gov/pubmed?term=Ma%20JZ%5BAuthor%5D&cauthor=true&cauthor_uid=20383528), [Payne TJ](http://www.ncbi.nlm.nih.gov/pubmed?term=Payne%20TJ%5BAuthor%5D&cauthor=true&cauthor_uid=20383528), [Cui W](http://www.ncbi.nlm.nih.gov/pubmed?term=Cui%20W%5BAuthor%5D&cauthor=true&cauthor_uid=20383528), [Ray R](http://www.ncbi.nlm.nih.gov/pubmed?term=Ray%20R%5BAuthor%5D&cauthor=true&cauthor_uid=20383528), et al. (2010) [Replication and extension of association of choline acetyltransferase with nicotine dependence in European and African American smokers.](http://www.ncbi.nlm.nih.gov/pubmed/20383528) Hum Genet 127:691-8.

**Table S3**. Estimated Beta Coefficients and *P*-Values for the Associations Between 321 SNPs And Number of Cigarettes Smoked and Nicotine Dependence Phenotypes. (n= 544). NDIT Study, 1999-2008.

| Gene | SNP | Phenotype | Estimated regression coefficient beta | Raw p-value from linear mixed models | Permutation-based p-value from linear mixed models* | FDR-corrected raw p-value from linear mixed models* | FDR-adjusted permutation-based p-value from linear mixed models* |
| --- | --- | --- | --- | --- | --- | --- | --- |
| *ANKK1* | rs10891545 | No. cigarettes | -0.38 | 0.06117 | 0.06182 | 0.68275 | 0.69122 |
|  |  | ND/craving | -0.67 | 0.22868 | 0.23154 | 0.81692 | 0.82073 |
|  |  | Self-medication | -0.20 | 0.18500 | 0.18864 | 0.81687 | 0.82073 |
|  |  | mFTQ | -0.20 | 0.09014 | 0.09156 | 0.71653 | 0.72276 |
|  |  | ICD-10 | -0.16 | 0.11013 | 0.11179 | 0.74516 | 0.75032 |
|  |  | Withdrawal | -0.35 | 0.25111 | 0.25159 | 0.81692 | 0.82073 |
|  | rs11214601 | No. cigarettes | 0.25 | 0.24314 | 0.24339 | 0.81692 | 0.82073 |
|  |  | ND/craving | 0.31 | 0.60527 | 0.60507 | 0.91171 | 0.91247 |
|  |  | Self-medication | 0.07 | 0.68881 | 0.69022 | 0.93909 | 0.94037 |
|  |  | mFTQ | 0.07 | 0.58464 | 0.58417 | 0.90443 | 0.90491 |
|  |  | ICD-10 | 0.09 | 0.38733 | 0.38670 | 0.84836 | 0.85020 |
|  |  | Withdrawal | 0.14 | 0.67558 | 0.67540 | 0.93294 | 0.93426 |
|  | rs11604671 | No. cigarettes | 0.35 | 0.02300 | 0.02351 | 0.61858 | 0.62356 |
|  |  | ND/craving | 0.58 | 0.17563 | 0.17668 | 0.81239 | 0.81790 |
|  |  | Self-medication | 0.19 | 0.09710 | 0.09852 | 0.71653 | 0.72775 |
|  |  | mFTQ | 0.17 | 0.06451 | 0.06509 | 0.68275 | 0.69122 |
|  |  | ICD-10 | 0.08 | 0.27868 | 0.27844 | 0.81692 | 0.82073 |
|  |  | Withdrawal | 0.43 | 0.06737 | 0.06634 | 0.68275 | 0.69122 |
|  | rs12422191† | No. cigarettes | -0.25 | 0.41380 | 0.41633 | 0.85330 | 0.85620 |
|  |  | ND/craving | -0.84 | 0.32987 | 0.33235 | 0.82975 | 0.83365 |
|  |  | Self-medication | -0.37 | 0.11211 | 0.11353 | 0.74516 | 0.75032 |
|  |  | mFTQ | -0.18 | 0.31974 | 0.32317 | 0.82787 | 0.83152 |
|  |  | ICD-10 | -0.19 | 0.23597 | 0.23704 | 0.81692 | 0.82073 |
|  |  | Withdrawal | -0.53 | 0.26763 | 0.27159 | 0.81692 | 0.82073 |
|  | rs1800497 | No. cigarettes | 0.11 | 0.55348 | 0.55368 | 0.89350 | 0.89469 |
|  |  | ND/craving | 0.01 | 0.98486 | 0.98447 | 0.99655 | 0.99647 |
|  |  | Self-medication | -0.01 | 0.96422 | 0.96394 | 0.99503 | 0.99540 |
|  |  | mFTQ | -0.01 | 0.93565 | 0.93689 | 0.98732 | 0.98766 |
|  |  | ICD-10 | 0.04 | 0.66731 | 0.66637 | 0.93268 | 0.93384 |
|  |  | Withdrawal | -0.11 | 0.70917 | 0.71022 | 0.94589 | 0.94578 |
|  | rs2242592 | No. cigarettes | -0.44 | 0.00594 | 0.00625 | 0.48756 | 0.49807 |
|  |  | ND/craving | -0.71 | 0.11656 | 0.11737 | 0.74516 | 0.75032 |
|  |  | Self-medication | -0.23 | 0.06465 | 0.06531 | 0.68275 | 0.69122 |
|  |  | mFTQ | -0.20 | 0.03631 | 0.03718 | 0.68275 | 0.69122 |
|  |  | ICD-10 | -0.11 | 0.17182 | 0.17341 | 0.81132 | 0.81734 |
|  |  | Withdrawal | -0.52 | 0.03445 | 0.03481 | 0.68275 | 0.69122 |
|  | rs2734848 | No. cigarettes | -0.32 | 0.09364 | 0.09453 | 0.71653 | 0.72775 |
|  |  | ND/craving | -0.71 | 0.19113 | 0.19279 | 0.81687 | 0.82073 |
|  |  | Self-medication | -0.13 | 0.39674 | 0.40101 | 0.84836 | 0.85368 |
|  |  | mFTQ | -0.17 | 0.12497 | 0.12805 | 0.76898 | 0.78540 |
|  |  | ICD-10 | -0.13 | 0.20002 | 0.20095 | 0.81687 | 0.82073 |
|  |  | Withdrawal | -0.55 | 0.06044 | 0.06137 | 0.68275 | 0.69122 |
|  | rs2734849 | No. cigarettes | 0.33 | 0.03290 | 0.03325 | 0.68275 | 0.69122 |
|  |  | ND/craving | 0.58 | 0.17902 | 0.17991 | 0.81687 | 0.82073 |
|  |  | Self-medication | 0.20 | 0.09441 | 0.09519 | 0.71653 | 0.72775 |
|  |  | mFTQ | 0.16 | 0.07503 | 0.07615 | 0.70208 | 0.71201 |
|  |  | ICD-10 | 0.08 | 0.29266 | 0.29303 | 0.81833 | 0.82138 |
|  |  | Withdrawal | 0.42 | 0.07119 | 0.06971 | 0.68903 | 0.69122 |
|  | rs754672 | No. cigarettes | 0.24 | 0.12669 | 0.12771 | 0.77712 | 0.78540 |
|  |  | ND/craving | 0.24 | 0.58654 | 0.58679 | 0.90520 | 0.90630 |
|  |  | Self-medication | 0.05 | 0.67465 | 0.67663 | 0.93294 | 0.93426 |
|  |  | mFTQ | 0.10 | 0.28722 | 0.28955 | 0.81833 | 0.82138 |
|  |  | ICD-10 | 0.01 | 0.93529 | 0.93524 | 0.98732 | 0.98766 |
|  |  | Withdrawal | 0.29 | 0.22455 | 0.22476 | 0.81692 | 0.82073 |
|  | rs877138 | No. cigarettes | -0.01 | 0.96293 | 0.96265 | 0.99503 | 0.99540 |
|  |  | ND/craving | 0.20 | 0.67400 | 0.67661 | 0.93294 | 0.93426 |
|  |  | Self-medication | 0.04 | 0.73289 | 0.73330 | 0.95166 | 0.95174 |
|  |  | mFTQ | 0.01 | 0.94523 | 0.94545 | 0.98902 | 0.98910 |
|  |  | ICD-10 | 0.09 | 0.28489 | 0.28734 | 0.81833 | 0.82138 |
|  |  | Withdrawal | -0.11 | 0.67274 | 0.67010 | 0.93294 | 0.93384 |
| *BDNF* | rs1013402 | No. cigarettes | 0.07 | 0.66978 | 0.67000 | 0.93275 | 0.93384 |
|  |  | ND/craving | -0.21 | 0.67574 | 0.67749 | 0.93294 | 0.93426 |
|  |  | Self-medication | -0.11 | 0.42722 | 0.42640 | 0.85803 | 0.86008 |
|  |  | mFTQ | -0.05 | 0.64468 | 0.64685 | 0.92403 | 0.92627 |
|  |  | ICD-10 | -0.07 | 0.45381 | 0.45334 | 0.86906 | 0.87224 |
|  |  | Withdrawal | -0.38 | 0.15544 | 0.15526 | 0.81063 | 0.81147 |
|  | rs10742179 | No. cigarettes | -0.13 | 0.45943 | 0.46148 | 0.86906 | 0.87224 |
|  |  | ND/craving | 0.29 | 0.56912 | 0.56718 | 0.89822 | 0.89972 |
|  |  | Self-medication | -0.02 | 0.86155 | 0.86330 | 0.97659 | 0.97697 |
|  |  | mFTQ | 0.06 | 0.57992 | 0.58007 | 0.90052 | 0.90294 |
|  |  | ICD-10 | 0.04 | 0.64792 | 0.64757 | 0.92516 | 0.92659 |
|  |  | Withdrawal | 0.16 | 0.55729 | 0.55768 | 0.89350 | 0.89469 |
|  | rs10767653 | No. cigarettes | -0.19 | 0.23405 | 0.23745 | 0.81692 | 0.82073 |
|  |  | ND/craving | -0.09 | 0.84263 | 0.84282 | 0.97554 | 0.97638 |
|  |  | Self-medication | -0.09 | 0.44757 | 0.44976 | 0.86722 | 0.87224 |
|  |  | mFTQ | -0.05 | 0.60396 | 0.60534 | 0.91171 | 0.91247 |
|  |  | ICD-10 | -0.03 | 0.73186 | 0.73049 | 0.95166 | 0.95174 |
|  |  | Withdrawal | -0.24 | 0.32863 | 0.32927 | 0.82975 | 0.83365 |
|  | rs10835201 | No. cigarettes | 0.16 | 0.36482 | 0.36480 | 0.84491 | 0.84876 |
|  |  | ND/craving | -0.12 | 0.81134 | 0.81321 | 0.96758 | 0.96905 |
|  |  | Self-medication | -0.01 | 0.96091 | 0.96058 | 0.99503 | 0.99540 |
|  |  | mFTQ | 0.00 | 0.98183 | 0.98187 | 0.99655 | 0.99647 |
|  |  | ICD-10 | -0.03 | 0.72834 | 0.72888 | 0.95166 | 0.95174 |
|  |  | Withdrawal | -0.29 | 0.28965 | 0.29111 | 0.81833 | 0.82138 |
|  | rs11030123 | No. cigarettes | -0.13 | 0.66678 | 0.66818 | 0.93268 | 0.93384 |
|  |  | ND/craving | -0.90 | 0.27928 | 0.28082 | 0.81692 | 0.82073 |
|  |  | Self-medication | -0.24 | 0.28139 | 0.28407 | 0.81833 | 0.82138 |
|  |  | mFTQ | -0.22 | 0.20006 | 0.20339 | 0.81687 | 0.82073 |
|  |  | ICD-10 | -0.22 | 0.14510 | 0.14575 | 0.79921 | 0.80409 |
|  |  | Withdrawal | -0.68 | 0.13115 | 0.13090 | 0.78201 | 0.78587 |
|  | rs12807253 | No. cigarettes | 0.19 | 0.48173 | 0.48565 | 0.87147 | 0.87340 |
|  |  | ND/craving | 0.40 | 0.59759 | 0.59767 | 0.91084 | 0.91141 |
|  |  | Self-medication | 0.04 | 0.85404 | 0.85507 | 0.97659 | 0.97697 |
|  |  | mFTQ | 0.23 | 0.14565 | 0.14744 | 0.79921 | 0.80409 |
|  |  | ICD-10 | 0.04 | 0.76613 | 0.76603 | 0.95851 | 0.95944 |
|  |  | Withdrawal | -0.04 | 0.92606 | 0.92591 | 0.98534 | 0.98615 |
|  | rs1491851 | No. cigarettes | -0.18 | 0.25411 | 0.25708 | 0.81692 | 0.82073 |
|  |  | ND/craving | -0.50 | 0.25421 | 0.25788 | 0.81692 | 0.82073 |
|  |  | Self-medication | -0.08 | 0.48518 | 0.48658 | 0.87147 | 0.87340 |
|  |  | mFTQ | -0.08 | 0.36666 | 0.36943 | 0.84491 | 0.84876 |
|  |  | ICD-10 | -0.09 | 0.26738 | 0.27197 | 0.81692 | 0.82073 |
|  |  | Withdrawal | -0.10 | 0.67795 | 0.67909 | 0.93333 | 0.93439 |
|  | rs2030324 | No. cigarettes | -0.02 | 0.87871 | 0.87722 | 0.97659 | 0.97697 |
|  |  | ND/craving | -0.28 | 0.51917 | 0.51969 | 0.88888 | 0.89054 |
|  |  | Self-medication | 0.00 | 0.98311 | 0.98310 | 0.99655 | 0.99647 |
|  |  | mFTQ | -0.04 | 0.62157 | 0.62223 | 0.91735 | 0.91973 |
|  |  | ICD-10 | 0.00 | 0.96830 | 0.96860 | 0.99655 | 0.99647 |
|  |  | Withdrawal | 0.05 | 0.83215 | 0.83144 | 0.97193 | 0.97182 |
|  | rs2049048 | No. cigarettes | 0.24 | 0.29151 | 0.29331 | 0.81833 | 0.82138 |
|  |  | ND/craving | 0.20 | 0.75520 | 0.75480 | 0.95749 | 0.95849 |
|  |  | Self-medication | 0.04 | 0.80602 | 0.80701 | 0.96758 | 0.96843 |
|  |  | mFTQ | 0.00 | 0.97298 | 0.97381 | 0.99655 | 0.99647 |
|  |  | ICD-10 | -0.04 | 0.75138 | 0.75252 | 0.95711 | 0.95794 |
|  |  | Withdrawal | 0.05 | 0.88819 | 0.88916 | 0.97659 | 0.97697 |
|  | rs2203877 | No. cigarettes | 0.09 | 0.54191 | 0.54308 | 0.89247 | 0.89469 |
|  |  | ND/craving | 0.00 | 0.99978 | 0.99969 | 0.99989 | 0.99990 |
|  |  | Self-medication | 0.02 | 0.86349 | 0.86382 | 0.97659 | 0.97697 |
|  |  | mFTQ | 0.02 | 0.78750 | 0.78793 | 0.96746 | 0.96783 |
|  |  | ICD-10 | 0.02 | 0.80581 | 0.80799 | 0.96758 | 0.96843 |
|  |  | Withdrawal | 0.14 | 0.53999 | 0.53892 | 0.89247 | 0.89469 |
|  | rs4074134 | No. cigarettes | -0.02 | 0.92098 | 0.92157 | 0.98436 | 0.98444 |
|  |  | ND/craving | 0.73 | 0.16789 | 0.16872 | 0.81132 | 0.81734 |
|  |  | Self-medication | 0.13 | 0.34893 | 0.34804 | 0.83692 | 0.83895 |
|  |  | mFTQ | 0.12 | 0.28154 | 0.28226 | 0.81833 | 0.82138 |
|  |  | ICD-10 | 0.11 | 0.25448 | 0.25536 | 0.81692 | 0.82073 |
|  |  | Withdrawal | 0.36 | 0.21130 | 0.21182 | 0.81692 | 0.82073 |
|  | rs4923456 | No. cigarettes | -0.19 | 0.27756 | 0.28048 | 0.81692 | 0.82073 |
|  |  | ND/craving | 0.11 | 0.82433 | 0.82438 | 0.96985 | 0.97009 |
|  |  | Self-medication | 0.04 | 0.78694 | 0.78586 | 0.96746 | 0.96713 |
|  |  | mFTQ | -0.01 | 0.94205 | 0.94288 | 0.98879 | 0.98910 |
|  |  | ICD-10 | 0.02 | 0.86176 | 0.86182 | 0.97659 | 0.97697 |
|  |  | Withdrawal | 0.11 | 0.67702 | 0.67920 | 0.93294 | 0.93439 |
|  | rs6265 | No. cigarettes | 0.04 | 0.85179 | 0.84988 | 0.97659 | 0.97697 |
|  |  | ND/craving | 0.76 | 0.16310 | 0.16288 | 0.81132 | 0.81697 |
|  |  | Self-medication | 0.12 | 0.43591 | 0.43761 | 0.85904 | 0.86375 |
|  |  | mFTQ | 0.16 | 0.16244 | 0.16219 | 0.81132 | 0.81586 |
|  |  | ICD-10 | 0.15 | 0.13462 | 0.13531 | 0.79100 | 0.79456 |
|  |  | Withdrawal | 0.44 | 0.13828 | 0.13927 | 0.79750 | 0.79829 |
|  | rs7927728† | No. cigarettes | -0.55 | 0.13910 | 0.13918 | 0.79750 | 0.79829 |
|  |  | ND/craving | -1.69 | 0.10337 | 0.10367 | 0.73197 | 0.73783 |
|  |  | Self-medication | -0.67 | 0.01759 | 0.01737 | 0.57781 | 0.58246 |
|  |  | mFTQ | -0.31 | 0.15594 | 0.15369 | 0.81063 | 0.81147 |
|  |  | ICD-10 | -0.24 | 0.19507 | 0.19631 | 0.81687 | 0.82073 |
|  |  | Withdrawal | -0.85 | 0.13726 | 0.13761 | 0.79750 | 0.79665 |
| *CHAT* | rs10857520 | No. cigarettes | 0.17 | 0.26133 | 0.26253 | 0.81692 | 0.82073 |
|  |  | ND/craving | 0.58 | 0.17129 | 0.17075 | 0.81132 | 0.81734 |
|  |  | Self-medication | 0.13 | 0.26932 | 0.27026 | 0.81692 | 0.82073 |
|  |  | mFTQ | 0.10 | 0.23428 | 0.23530 | 0.81692 | 0.82073 |
|  |  | ICD-10 | 0.10 | 0.18218 | 0.18359 | 0.81687 | 0.82073 |
|  |  | Withdrawal | 0.32 | 0.16779 | 0.16856 | 0.81132 | 0.81734 |
|  | rs11101179 | No. cigarettes | -0.13 | 0.45491 | 0.45879 | 0.86906 | 0.87224 |
|  |  | ND/craving | 0.11 | 0.81533 | 0.81800 | 0.96885 | 0.97009 |
|  |  | Self-medication | 0.02 | 0.90414 | 0.90246 | 0.98078 | 0.98062 |
|  |  | mFTQ | 0.07 | 0.51523 | 0.51928 | 0.88888 | 0.89054 |
|  |  | ICD-10 | 0.03 | 0.74277 | 0.74521 | 0.95464 | 0.95552 |
|  |  | Withdrawal | -0.16 | 0.53855 | 0.54265 | 0.89247 | 0.89469 |
|  | rs11101182 | No. cigarettes | -0.34 | 0.19031 | 0.19291 | 0.81687 | 0.82073 |
|  |  | ND/craving | -0.38 | 0.60089 | 0.60207 | 0.91171 | 0.91247 |
|  |  | Self-medication | -0.14 | 0.46905 | 0.46957 | 0.87147 | 0.87340 |
|  |  | mFTQ | -0.06 | 0.68074 | 0.68171 | 0.93525 | 0.93717 |
|  |  | ICD-10 | -0.12 | 0.37814 | 0.37845 | 0.84491 | 0.84876 |
|  |  | Withdrawal | -0.08 | 0.84894 | 0.84869 | 0.97659 | 0.97697 |
|  | rs11101200 | No. cigarettes | 0.04 | 0.80197 | 0.80296 | 0.96758 | 0.96843 |
|  |  | ND/craving | -0.14 | 0.75193 | 0.75241 | 0.95719 | 0.95794 |
|  |  | Self-medication | -0.04 | 0.71140 | 0.71200 | 0.94689 | 0.94578 |
|  |  | mFTQ | -0.01 | 0.87653 | 0.87632 | 0.97659 | 0.97697 |
|  |  | ICD-10 | -0.01 | 0.93605 | 0.93674 | 0.98732 | 0.98766 |
|  |  | Withdrawal | -0.08 | 0.74349 | 0.74280 | 0.95464 | 0.95503 |
|  | rs11101204 | No. cigarettes | -0.11 | 0.52383 | 0.52428 | 0.88948 | 0.89054 |
|  |  | ND/craving | -0.07 | 0.87986 | 0.88016 | 0.97659 | 0.97697 |
|  |  | Self-medication | 0.00 | 0.96961 | 0.96987 | 0.99655 | 0.99647 |
|  |  | mFTQ | 0.01 | 0.94258 | 0.94383 | 0.98879 | 0.98910 |
|  |  | ICD-10 | 0.01 | 0.92651 | 0.92768 | 0.98534 | 0.98638 |
|  |  | Withdrawal | -0.18 | 0.47647 | 0.47682 | 0.87147 | 0.87340 |
|  | rs11591558 | No. cigarettes | 0.10 | 0.54441 | 0.54494 | 0.89247 | 0.89469 |
|  |  | ND/craving | 0.47 | 0.32459 | 0.32397 | 0.82813 | 0.83152 |
|  |  | Self-medication | 0.04 | 0.73185 | 0.73123 | 0.95166 | 0.95174 |
|  |  | mFTQ | 0.12 | 0.22679 | 0.22738 | 0.81692 | 0.82073 |
|  |  | ICD-10 | 0.08 | 0.33690 | 0.33892 | 0.82975 | 0.83365 |
|  |  | Withdrawal | 0.00 | 0.99109 | 0.99072 | 0.99669 | 0.99647 |
|  | rs12217567 | No. cigarettes | 0.14 | 0.42468 | 0.42781 | 0.85803 | 0.86008 |
|  |  | ND/craving | 0.48 | 0.31071 | 0.31368 | 0.82443 | 0.82983 |
|  |  | Self-medication | 0.18 | 0.17244 | 0.17411 | 0.81132 | 0.81734 |
|  |  | mFTQ | 0.09 | 0.33908 | 0.34167 | 0.83024 | 0.83615 |
|  |  | ICD-10 | 0.07 | 0.41671 | 0.41752 | 0.85388 | 0.85620 |
|  |  | Withdrawal | 0.31 | 0.21905 | 0.22040 | 0.81692 | 0.82073 |
|  | rs12246528 | No. cigarettes | 0.06 | 0.72912 | 0.72815 | 0.95166 | 0.95174 |
|  |  | ND/craving | 0.27 | 0.58591 | 0.58598 | 0.90520 | 0.90578 |
|  |  | Self-medication | 0.06 | 0.66721 | 0.67034 | 0.93268 | 0.93384 |
|  |  | mFTQ | 0.03 | 0.73242 | 0.73307 | 0.95166 | 0.95174 |
|  |  | ICD-10 | 0.05 | 0.59135 | 0.58922 | 0.90572 | 0.90738 |
|  |  | Withdrawal | 0.33 | 0.21745 | 0.21618 | 0.81692 | 0.82073 |
|  | rs12266458 | No. cigarettes | -0.51 | 0.06406 | 0.06579 | 0.68275 | 0.69122 |
|  |  | ND/craving | -0.88 | 0.25173 | 0.25519 | 0.81692 | 0.82073 |
|  |  | Self-medication | -0.21 | 0.31221 | 0.31551 | 0.82443 | 0.82983 |
|  |  | mFTQ | -0.34 | 0.03449 | 0.03504 | 0.68275 | 0.69122 |
|  |  | ICD-10 | -0.09 | 0.53308 | 0.53687 | 0.89247 | 0.89469 |
|  |  | Withdrawal | -0.42 | 0.31291 | 0.31459 | 0.82443 | 0.82983 |
|  | rs1917810 | No. cigarettes | 0.09 | 0.57088 | 0.57286 | 0.89822 | 0.90267 |
|  |  | ND/craving | 0.58 | 0.18643 | 0.18712 | 0.81687 | 0.82073 |
|  |  | Self-medication | 0.13 | 0.26326 | 0.26330 | 0.81692 | 0.82073 |
|  |  | mFTQ | 0.11 | 0.21812 | 0.21969 | 0.81692 | 0.82073 |
|  |  | ICD-10 | 0.05 | 0.49549 | 0.49555 | 0.87955 | 0.88128 |
|  |  | Withdrawal | 0.29 | 0.22550 | 0.22516 | 0.81692 | 0.82073 |
|  | rs1917818 | No. cigarettes | 0.20 | 0.19530 | 0.19660 | 0.81687 | 0.82073 |
|  |  | ND/craving | 0.48 | 0.27497 | 0.27836 | 0.81692 | 0.82073 |
|  |  | Self-medication | 0.12 | 0.30817 | 0.31241 | 0.82443 | 0.82983 |
|  |  | mFTQ | 0.11 | 0.22903 | 0.23050 | 0.81692 | 0.82073 |
|  |  | ICD-10 | 0.07 | 0.37520 | 0.37947 | 0.84491 | 0.84876 |
|  |  | Withdrawal | 0.25 | 0.29206 | 0.29446 | 0.81833 | 0.82138 |
|  | rs2177370 | No. cigarettes | -0.17 | 0.27952 | 0.27878 | 0.81692 | 0.82073 |
|  |  | ND/craving | -0.36 | 0.40748 | 0.40786 | 0.85202 | 0.85488 |
|  |  | Self-medication | -0.12 | 0.33163 | 0.33334 | 0.82975 | 0.83365 |
|  |  | mFTQ | -0.06 | 0.54393 | 0.54589 | 0.89247 | 0.89469 |
|  |  | ICD-10 | -0.09 | 0.26394 | 0.26687 | 0.81692 | 0.82073 |
|  |  | Withdrawal | -0.18 | 0.43982 | 0.44069 | 0.86332 | 0.86652 |
|  | rs2377877 | No. cigarettes | -0.14 | 0.59252 | 0.59479 | 0.90572 | 0.90846 |
|  |  | ND/craving | -1.09 | 0.14546 | 0.14778 | 0.79921 | 0.80409 |
|  |  | Self-medication | -0.32 | 0.11396 | 0.11442 | 0.74516 | 0.75032 |
|  |  | mFTQ | -0.25 | 0.11473 | 0.11647 | 0.74516 | 0.75032 |
|  |  | ICD-10 | -0.15 | 0.26204 | 0.26334 | 0.81692 | 0.82073 |
|  |  | Withdrawal | -0.38 | 0.35175 | 0.35350 | 0.83974 | 0.84367 |
|  | rs2889759 | No. cigarettes | 0.04 | 0.89632 | 0.89858 | 0.97915 | 0.98062 |
|  |  | ND/craving | -0.29 | 0.73948 | 0.74123 | 0.95259 | 0.95428 |
|  |  | Self-medication | -0.10 | 0.68718 | 0.68861 | 0.93866 | 0.94037 |
|  |  | mFTQ | -0.11 | 0.55688 | 0.56137 | 0.89350 | 0.89577 |
|  |  | ICD-10 | -0.05 | 0.75823 | 0.76053 | 0.95749 | 0.95943 |
|  |  | Withdrawal | -0.49 | 0.30298 | 0.30542 | 0.82443 | 0.82839 |
|  | rs3729496 | No. cigarettes | -0.11 | 0.54702 | 0.54947 | 0.89247 | 0.89469 |
|  |  | ND/craving | -0.49 | 0.31718 | 0.31668 | 0.82728 | 0.82983 |
|  |  | Self-medication | -0.16 | 0.23037 | 0.23259 | 0.81692 | 0.82073 |
|  |  | mFTQ | -0.12 | 0.22352 | 0.22695 | 0.81692 | 0.82073 |
|  |  | ICD-10 | -0.10 | 0.25534 | 0.25680 | 0.81692 | 0.82073 |
|  |  | Withdrawal | -0.29 | 0.27576 | 0.27341 | 0.81692 | 0.82073 |
|  | rs3750752 | No. cigarettes | 0.43 | 0.20344 | 0.20571 | 0.81687 | 0.82073 |
|  |  | ND/craving | 0.71 | 0.45435 | 0.45523 | 0.86906 | 0.87224 |
|  |  | Self-medication | 0.12 | 0.63563 | 0.63902 | 0.92368 | 0.92547 |
|  |  | mFTQ | 0.08 | 0.67200 | 0.67216 | 0.93294 | 0.93404 |
|  |  | ICD-10 | 0.11 | 0.52193 | 0.52498 | 0.88888 | 0.89054 |
|  |  | Withdrawal | 0.06 | 0.90440 | 0.90679 | 0.98078 | 0.98172 |
|  | rs3793790 | No. cigarettes | 0.20 | 0.25224 | 0.25404 | 0.81692 | 0.82073 |
|  |  | ND/craving | 0.41 | 0.38762 | 0.39173 | 0.84836 | 0.85202 |
|  |  | Self-medication | 0.01 | 0.94554 | 0.94493 | 0.98902 | 0.98910 |
|  |  | mFTQ | 0.06 | 0.57112 | 0.57578 | 0.89822 | 0.90267 |
|  |  | ICD-10 | 0.08 | 0.34833 | 0.35311 | 0.83652 | 0.84367 |
|  |  | Withdrawal | 0.12 | 0.63655 | 0.63786 | 0.92368 | 0.92547 |
|  | rs3793791 | No. cigarettes | -0.16 | 0.54850 | 0.54731 | 0.89247 | 0.89469 |
|  |  | ND/craving | -0.66 | 0.37304 | 0.37221 | 0.84491 | 0.84876 |
|  |  | Self-medication | -0.20 | 0.31595 | 0.31559 | 0.82728 | 0.82983 |
|  |  | mFTQ | -0.11 | 0.48397 | 0.48366 | 0.87147 | 0.87340 |
|  |  | ICD-10 | -0.11 | 0.40206 | 0.40113 | 0.85202 | 0.85368 |
|  |  | Withdrawal | -0.57 | 0.15800 | 0.15831 | 0.81063 | 0.81586 |
|  | rs3810950 | No. cigarettes | 0.20 | 0.27146 | 0.27181 | 0.81692 | 0.82073 |
|  |  | ND/craving | 0.69 | 0.17094 | 0.17028 | 0.81132 | 0.81734 |
|  |  | Self-medication | 0.15 | 0.27807 | 0.27842 | 0.81692 | 0.82073 |
|  |  | mFTQ | 0.18 | 0.07761 | 0.07728 | 0.71245 | 0.71554 |
|  |  | ICD-10 | 0.10 | 0.27821 | 0.27690 | 0.81692 | 0.82073 |
|  |  | Withdrawal | 0.10 | 0.72079 | 0.72173 | 0.94944 | 0.95098 |
|  | rs3810951 | No. cigarettes | -0.20 | 0.51399 | 0.51833 | 0.88888 | 0.89054 |
|  |  | ND/craving | -0.99 | 0.25607 | 0.25884 | 0.81692 | 0.82073 |
|  |  | Self-medication | -0.24 | 0.31867 | 0.32216 | 0.82728 | 0.83152 |
|  |  | mFTQ | -0.21 | 0.24402 | 0.24764 | 0.81692 | 0.82073 |
|  |  | ICD-10 | -0.17 | 0.26852 | 0.27124 | 0.81692 | 0.82073 |
|  |  | Withdrawal | -0.42 | 0.36644 | 0.36617 | 0.84491 | 0.84876 |
|  | rs4838392 | No. cigarettes | 0.06 | 0.70120 | 0.69991 | 0.94243 | 0.94333 |
|  |  | ND/craving | 0.06 | 0.90033 | 0.90189 | 0.98061 | 0.98062 |
|  |  | Self-medication | -0.01 | 0.93469 | 0.93587 | 0.98732 | 0.98766 |
|  |  | mFTQ | -0.02 | 0.80785 | 0.80721 | 0.96758 | 0.96843 |
|  |  | ICD-10 | 0.00 | 0.98461 | 0.98462 | 0.99655 | 0.99647 |
|  |  | Withdrawal | 0.13 | 0.60075 | 0.60148 | 0.91171 | 0.91247 |
|  | rs7067651 | No. cigarettes | -0.06 | 0.77068 | 0.77172 | 0.96011 | 0.96140 |
|  |  | ND/craving | -0.18 | 0.76427 | 0.76594 | 0.95770 | 0.95944 |
|  |  | Self-medication | -0.08 | 0.61153 | 0.61359 | 0.91338 | 0.91505 |
|  |  | mFTQ | -0.11 | 0.35103 | 0.35282 | 0.83974 | 0.84367 |
|  |  | ICD-10 | -0.03 | 0.78945 | 0.79038 | 0.96758 | 0.96843 |
|  |  | Withdrawal | 0.12 | 0.71439 | 0.71671 | 0.94741 | 0.94996 |
|  | rs7076926 | No. cigarettes | -0.40 | 0.12882 | 0.13021 | 0.77775 | 0.78587 |
|  |  | ND/craving | -1.32 | 0.07430 | 0.07519 | 0.70147 | 0.70910 |
|  |  | Self-medication | -0.26 | 0.20007 | 0.20169 | 0.81687 | 0.82073 |
|  |  | mFTQ | -0.31 | 0.04171 | 0.04305 | 0.68275 | 0.69122 |
|  |  | ICD-10 | -0.16 | 0.22182 | 0.22235 | 0.81692 | 0.82073 |
|  |  | Withdrawal | -0.71 | 0.07036 | 0.07088 | 0.68903 | 0.69122 |
|  | rs7091005 | No. cigarettes | -0.02 | 0.95347 | 0.95403 | 0.99193 | 0.99216 |
|  |  | ND/craving | -0.35 | 0.62905 | 0.62870 | 0.92274 | 0.92348 |
|  |  | Self-medication | -0.12 | 0.55121 | 0.55258 | 0.89247 | 0.89469 |
|  |  | mFTQ | -0.14 | 0.37420 | 0.37637 | 0.84491 | 0.84876 |
|  |  | ICD-10 | -0.05 | 0.72980 | 0.73235 | 0.95166 | 0.95174 |
|  |  | Withdrawal | -0.35 | 0.37686 | 0.37839 | 0.84491 | 0.84876 |
|  | rs733722 | No. cigarettes | 0.18 | 0.36804 | 0.37150 | 0.84491 | 0.84876 |
|  |  | ND/craving | 0.25 | 0.65573 | 0.65806 | 0.92727 | 0.92887 |
|  |  | Self-medication | 0.11 | 0.47084 | 0.47580 | 0.87147 | 0.87340 |
|  |  | mFTQ | 0.06 | 0.59089 | 0.59224 | 0.90572 | 0.90766 |
|  |  | ICD-10 | 0.02 | 0.83872 | 0.83989 | 0.97441 | 0.97564 |
|  |  | Withdrawal | 0.28 | 0.35723 | 0.36094 | 0.84407 | 0.84859 |
|  | rs7903496 | No. cigarettes | 0.21 | 0.17588 | 0.17567 | 0.81239 | 0.81734 |
|  |  | ND/craving | 0.76 | 0.07626 | 0.07697 | 0.70952 | 0.71554 |
|  |  | Self-medication | 0.20 | 0.08525 | 0.08714 | 0.71335 | 0.72276 |
|  |  | mFTQ | 0.14 | 0.12867 | 0.13051 | 0.77775 | 0.78587 |
|  |  | ICD-10 | 0.12 | 0.11647 | 0.11840 | 0.74516 | 0.75260 |
|  |  | Withdrawal | 0.29 | 0.20751 | 0.20979 | 0.81692 | 0.82073 |
|  | rs8178980† | No. cigarettes | -0.52 | 0.15326 | 0.15519 | 0.81063 | 0.81147 |
|  |  | ND/craving | -0.86 | 0.39290 | 0.39423 | 0.84836 | 0.85202 |
|  |  | Self-medication | -0.09 | 0.75291 | 0.75446 | 0.95749 | 0.95849 |
|  |  | mFTQ | -0.13 | 0.54046 | 0.54008 | 0.89247 | 0.89469 |
|  |  | ICD-10 | -0.09 | 0.63382 | 0.63492 | 0.92368 | 0.92547 |
|  |  | Withdrawal | 0.09 | 0.86561 | 0.86715 | 0.97659 | 0.97697 |
|  | rs8178990† | No. cigarettes | 0.35 | 0.32446 | 0.32725 | 0.82813 | 0.83152 |
|  |  | ND/craving | 1.27 | 0.20170 | 0.20285 | 0.81687 | 0.82073 |
|  |  | Self-medication | 0.28 | 0.30222 | 0.30435 | 0.82443 | 0.82839 |
|  |  | mFTQ | 0.16 | 0.44747 | 0.44946 | 0.86722 | 0.87224 |
|  |  | ICD-10 | 0.21 | 0.24402 | 0.24540 | 0.81692 | 0.82073 |
|  |  | Withdrawal | 0.07 | 0.90111 | 0.90301 | 0.98061 | 0.98062 |
|  | rs868750 | No. cigarettes | 0.17 | 0.42903 | 0.42721 | 0.85895 | 0.86008 |
|  |  | ND/craving | 0.41 | 0.48641 | 0.48517 | 0.87147 | 0.87340 |
|  |  | Self-medication | 0.11 | 0.48417 | 0.48334 | 0.87147 | 0.87340 |
|  |  | mFTQ | 0.09 | 0.46670 | 0.46462 | 0.87147 | 0.87340 |
|  |  | ICD-10 | 0.04 | 0.73653 | 0.73804 | 0.95166 | 0.95343 |
|  |  | Withdrawal | 0.59 | 0.06261 | 0.06201 | 0.68275 | 0.69122 |
|  | rs885834 | No. cigarettes | 0.09 | 0.58299 | 0.58548 | 0.90261 | 0.90574 |
|  |  | ND/craving | -0.10 | 0.82614 | 0.82555 | 0.96998 | 0.97071 |
|  |  | Self-medication | -0.03 | 0.79379 | 0.79368 | 0.96758 | 0.96843 |
|  |  | mFTQ | -0.08 | 0.40576 | 0.40796 | 0.85202 | 0.85488 |
|  |  | ICD-10 | -0.03 | 0.69788 | 0.69839 | 0.94166 | 0.94333 |
|  |  | Withdrawal | 0.12 | 0.63062 | 0.63334 | 0.92368 | 0.92547 |
| *CHRNA3* | rs1051730 | No. cigarettes | 0.06 | 0.71869 | 0.72125 | 0.94944 | 0.95098 |
|  |  | ND/craving | 0.07 | 0.87972 | 0.88004 | 0.97659 | 0.97697 |
|  |  | Self-medication | -0.03 | 0.82562 | 0.82699 | 0.96998 | 0.97121 |
|  |  | mFTQ | 0.06 | 0.57657 | 0.57845 | 0.89923 | 0.90267 |
|  |  | ICD-10 | -0.03 | 0.75996 | 0.76067 | 0.95749 | 0.95943 |
|  |  | Withdrawal | 0.02 | 0.95226 | 0.95334 | 0.99193 | 0.99216 |
|  | rs3743077 | No. cigarettes | -0.01 | 0.93209 | 0.93194 | 0.98732 | 0.98766 |
|  |  | ND/craving | 0.04 | 0.93386 | 0.93481 | 0.98732 | 0.98766 |
|  |  | Self-medication | -0.01 | 0.96604 | 0.96660 | 0.99583 | 0.99647 |
|  |  | mFTQ | -0.04 | 0.63762 | 0.63688 | 0.92368 | 0.92547 |
|  |  | ICD-10 | 0.00 | 0.99119 | 0.99078 | 0.99669 | 0.99647 |
|  |  | Withdrawal | -0.05 | 0.85052 | 0.85206 | 0.97659 | 0.97697 |
|  | rs4887069 | No. cigarettes | -0.04 | 0.81299 | 0.81329 | 0.96846 | 0.96905 |
|  |  | ND/craving | -0.01 | 0.98252 | 0.98240 | 0.99655 | 0.99647 |
|  |  | Self-medication | 0.05 | 0.72337 | 0.72348 | 0.94944 | 0.95098 |
|  |  | mFTQ | 0.04 | 0.72257 | 0.72331 | 0.94944 | 0.95098 |
|  |  | ICD-10 | 0.05 | 0.61040 | 0.61397 | 0.91320 | 0.91505 |
|  |  | Withdrawal | 0.07 | 0.80593 | 0.80752 | 0.96758 | 0.96843 |
|  | rs578776 | No. cigarettes | -0.02 | 0.90152 | 0.90323 | 0.98061 | 0.98062 |
|  |  | ND/craving | 0.18 | 0.71389 | 0.71016 | 0.94741 | 0.94578 |
|  |  | Self-medication | 0.12 | 0.36153 | 0.36129 | 0.84491 | 0.84859 |
|  |  | mFTQ | 0.02 | 0.80577 | 0.80580 | 0.96758 | 0.96843 |
|  |  | ICD-10 | 0.07 | 0.42974 | 0.42739 | 0.85904 | 0.86008 |
|  |  | Withdrawal | 0.27 | 0.30560 | 0.30366 | 0.82443 | 0.82839 |
| *CHRNA4* | rs1044396 | No. cigarettes | 0.15 | 0.32660 | 0.32854 | 0.82817 | 0.83365 |
|  |  | ND/craving | 0.09 | 0.83211 | 0.83364 | 0.97193 | 0.97322 |
|  |  | Self-medication | -0.03 | 0.79781 | 0.80074 | 0.96758 | 0.96843 |
|  |  | mFTQ | -0.01 | 0.88287 | 0.88175 | 0.97659 | 0.97697 |
|  |  | ICD-10 | 0.05 | 0.53880 | 0.53915 | 0.89247 | 0.89469 |
|  |  | Withdrawal | 0.11 | 0.62280 | 0.62523 | 0.91847 | 0.92275 |
|  | rs2093107 | No. cigarettes | -0.65 | 0.01091 | 0.01116 | 0.52414 | 0.53402 |
|  |  | ND/craving | -1.32 | 0.06743 | 0.06902 | 0.68275 | 0.69122 |
|  |  | Self-medication | -0.27 | 0.17275 | 0.17463 | 0.81132 | 0.81734 |
|  |  | mFTQ | -0.29 | 0.05777 | 0.05982 | 0.68275 | 0.69122 |
|  |  | ICD-10 | -0.21 | 0.11053 | 0.11153 | 0.74516 | 0.75032 |
|  |  | Withdrawal | -0.38 | 0.33625 | 0.33935 | 0.82975 | 0.83365 |
|  | rs3787138 | No. cigarettes | 0.21 | 0.35279 | 0.35502 | 0.84094 | 0.84519 |
|  |  | ND/craving | 0.15 | 0.80739 | 0.80565 | 0.96758 | 0.96843 |
|  |  | Self-medication | -0.02 | 0.92644 | 0.92653 | 0.98534 | 0.98615 |
|  |  | mFTQ | 0.12 | 0.37471 | 0.37416 | 0.84491 | 0.84876 |
|  |  | ICD-10 | 0.00 | 0.98565 | 0.98543 | 0.99655 | 0.99647 |
|  |  | Withdrawal | -0.40 | 0.23271 | 0.23534 | 0.81692 | 0.82073 |
|  | rs4522666 | No. cigarettes | -0.30 | 0.06664 | 0.06567 | 0.68275 | 0.69122 |
|  |  | ND/craving | -0.42 | 0.35185 | 0.35194 | 0.83974 | 0.84307 |
|  |  | Self-medication | -0.08 | 0.50505 | 0.50559 | 0.88796 | 0.89019 |
|  |  | mFTQ | -0.11 | 0.24598 | 0.24772 | 0.81692 | 0.82073 |
|  |  | ICD-10 | -0.10 | 0.22670 | 0.22802 | 0.81692 | 0.82073 |
|  |  | Withdrawal | -0.25 | 0.31334 | 0.31376 | 0.82443 | 0.82983 |
|  | rs4809538 | No. cigarettes | -0.09 | 0.60130 | 0.60248 | 0.91171 | 0.91247 |
|  |  | ND/craving | 0.13 | 0.80187 | 0.80130 | 0.96758 | 0.96843 |
|  |  | Self-medication | 0.08 | 0.55995 | 0.55870 | 0.89357 | 0.89469 |
|  |  | mFTQ | 0.06 | 0.55098 | 0.55157 | 0.89247 | 0.89469 |
|  |  | ICD-10 | 0.03 | 0.75987 | 0.75820 | 0.95749 | 0.95912 |
|  |  | Withdrawal | 0.26 | 0.33680 | 0.33683 | 0.82975 | 0.83365 |
|  | rs6011747† | No. cigarettes | 0.01 | 0.98395 | 0.98397 | 0.99655 | 0.99647 |
|  |  | ND/craving | 0.51 | 0.57502 | 0.57621 | 0.89916 | 0.90267 |
|  |  | Self-medication | 0.11 | 0.65456 | 0.65526 | 0.92697 | 0.92797 |
|  |  | mFTQ | 0.05 | 0.80714 | 0.80893 | 0.96758 | 0.96843 |
|  |  | ICD-10 | 0.01 | 0.95247 | 0.95125 | 0.99193 | 0.99216 |
|  |  | Withdrawal | 0.34 | 0.48536 | 0.48550 | 0.87147 | 0.87340 |
|  | rs735501 | No. cigarettes | -0.02 | 0.92266 | 0.92392 | 0.98451 | 0.98586 |
|  |  | ND/craving | 0.15 | 0.80196 | 0.80387 | 0.96758 | 0.96843 |
|  |  | Self-medication | 0.11 | 0.50729 | 0.50810 | 0.88805 | 0.89032 |
|  |  | mFTQ | 0.07 | 0.57025 | 0.57425 | 0.89822 | 0.90267 |
|  |  | ICD-10 | -0.05 | 0.66835 | 0.67177 | 0.93275 | 0.93404 |
|  |  | Withdrawal | 0.23 | 0.46618 | 0.46750 | 0.87147 | 0.87340 |
|  | rs755203 | No. cigarettes | -0.20 | 0.19678 | 0.19895 | 0.81687 | 0.82073 |
|  |  | ND/craving | -0.28 | 0.50632 | 0.50695 | 0.88805 | 0.89019 |
|  |  | Self-medication | 0.00 | 0.97172 | 0.97167 | 0.99655 | 0.99647 |
|  |  | mFTQ | 0.02 | 0.85250 | 0.85379 | 0.97659 | 0.97697 |
|  |  | ICD-10 | -0.08 | 0.31980 | 0.32012 | 0.82787 | 0.83152 |
|  |  | Withdrawal | -0.04 | 0.86979 | 0.87201 | 0.97659 | 0.97697 |
|  | rs755204 | No. cigarettes | 0.49 | 0.09844 | 0.09999 | 0.71653 | 0.72775 |
|  |  | ND/craving | 0.90 | 0.27627 | 0.27946 | 0.81692 | 0.82073 |
|  |  | Self-medication | 0.06 | 0.78014 | 0.78015 | 0.96379 | 0.96319 |
|  |  | mFTQ | 0.33 | 0.05210 | 0.05198 | 0.68275 | 0.69122 |
|  |  | ICD-10 | 0.09 | 0.54511 | 0.54686 | 0.89247 | 0.89469 |
|  |  | Withdrawal | -0.16 | 0.72114 | 0.72328 | 0.94944 | 0.95098 |
|  | rs9680065 | No. cigarettes | -0.16 | 0.31547 | 0.31600 | 0.82728 | 0.82983 |
|  |  | ND/craving | 0.04 | 0.92246 | 0.92141 | 0.98451 | 0.98444 |
|  |  | Self-medication | 0.05 | 0.67592 | 0.67537 | 0.93294 | 0.93426 |
|  |  | mFTQ | 0.00 | 0.98172 | 0.98167 | 0.99655 | 0.99647 |
|  |  | ICD-10 | -0.01 | 0.87301 | 0.87189 | 0.97659 | 0.97697 |
|  |  | Withdrawal | 0.19 | 0.42723 | 0.42572 | 0.85803 | 0.86008 |
| *CHRNA5* | rs11637635 | No. cigarettes | -0.01 | 0.93405 | 0.93353 | 0.98732 | 0.98766 |
|  |  | ND/craving | -0.06 | 0.88979 | 0.88879 | 0.97659 | 0.97697 |
|  |  | Self-medication | -0.08 | 0.54089 | 0.54032 | 0.89247 | 0.89469 |
|  |  | mFTQ | -0.05 | 0.55999 | 0.55880 | 0.89357 | 0.89469 |
|  |  | ICD-10 | 0.00 | 0.95969 | 0.96105 | 0.99503 | 0.99540 |
|  |  | Withdrawal | -0.12 | 0.61979 | 0.61634 | 0.91735 | 0.91665 |
|  | rs16969968 | No. cigarettes | 0.09 | 0.59163 | 0.59286 | 0.90572 | 0.90766 |
|  |  | ND/craving | 0.20 | 0.67409 | 0.67546 | 0.93294 | 0.93426 |
|  |  | Self-medication | 0.00 | 0.99439 | 0.99426 | 0.99801 | 0.99789 |
|  |  | mFTQ | 0.08 | 0.42609 | 0.42910 | 0.85803 | 0.86099 |
|  |  | ICD-10 | 0.00 | 0.96406 | 0.96439 | 0.99503 | 0.99540 |
|  |  | Withdrawal | 0.06 | 0.82391 | 0.82374 | 0.96985 | 0.97009 |
|  | rs569207 | No. cigarettes | 0.14 | 0.44300 | 0.44433 | 0.86364 | 0.86705 |
|  |  | ND/craving | 0.21 | 0.68636 | 0.68666 | 0.93866 | 0.93995 |
|  |  | Self-medication | 0.09 | 0.52417 | 0.52493 | 0.88948 | 0.89054 |
|  |  | mFTQ | 0.09 | 0.43395 | 0.43636 | 0.85904 | 0.86375 |
|  |  | ICD-10 | 0.09 | 0.36513 | 0.36635 | 0.84491 | 0.84876 |
|  |  | Withdrawal | 0.09 | 0.74478 | 0.74537 | 0.95528 | 0.95552 |
| *CHRNA6* | rs7828365 | No. cigarettes | 0.17 | 0.46727 | 0.46637 | 0.87147 | 0.87340 |
|  |  | ND/craving | 0.71 | 0.26341 | 0.26342 | 0.81692 | 0.82073 |
|  |  | Self-medication | 0.19 | 0.27463 | 0.27428 | 0.81692 | 0.82073 |
|  |  | mFTQ | 0.15 | 0.24069 | 0.24085 | 0.81692 | 0.82073 |
|  |  | ICD-10 | 0.04 | 0.73517 | 0.73621 | 0.95166 | 0.95343 |
|  |  | Withdrawal | 0.14 | 0.68080 | 0.68227 | 0.93525 | 0.93727 |
|  | rs7812298 | No. cigarettes | 0.08 | 0.66165 | 0.66072 | 0.93018 | 0.92955 |
|  |  | ND/craving | 0.49 | 0.35084 | 0.34847 | 0.83974 | 0.83895 |
|  |  | Self-medication | 0.08 | 0.56989 | 0.56771 | 0.89822 | 0.89972 |
|  |  | mFTQ | 0.12 | 0.27138 | 0.27227 | 0.81692 | 0.82073 |
|  |  | ICD-10 | 0.04 | 0.69867 | 0.69915 | 0.94166 | 0.94333 |
|  |  | Withdrawal | 0.20 | 0.47576 | 0.47567 | 0.87147 | 0.87340 |
|  | rs2304297 | No. cigarettes | 0.03 | 0.87533 | 0.87771 | 0.97659 | 0.97697 |
|  |  | ND/craving | 0.36 | 0.47800 | 0.47926 | 0.87147 | 0.87340 |
|  |  | Self-medication | 0.04 | 0.74834 | 0.74827 | 0.95566 | 0.95631 |
|  |  | mFTQ | 0.06 | 0.57311 | 0.57372 | 0.89916 | 0.90267 |
|  |  | ICD-10 | 0.03 | 0.72416 | 0.72378 | 0.94944 | 0.95098 |
|  |  | Withdrawal | 0.23 | 0.39445 | 0.39468 | 0.84836 | 0.85202 |
|  | rs17621710 | No. cigarettes | -0.37 | 0.15225 | 0.15520 | 0.81063 | 0.81147 |
|  |  | ND/craving | -0.95 | 0.19475 | 0.19588 | 0.81687 | 0.82073 |
|  |  | Self-medication | -0.23 | 0.24762 | 0.25059 | 0.81692 | 0.82073 |
|  |  | mFTQ | -0.20 | 0.19075 | 0.19420 | 0.81687 | 0.82073 |
|  |  | ICD-10 | -0.16 | 0.23260 | 0.23440 | 0.81692 | 0.82073 |
|  |  | Withdrawal | -0.65 | 0.10262 | 0.10369 | 0.73197 | 0.73783 |
|  | rs16891604† | No. cigarettes | -0.64 | 0.07944 | 0.08004 | 0.71335 | 0.72276 |
|  |  | ND/craving | -0.93 | 0.36553 | 0.36555 | 0.84491 | 0.84876 |
|  |  | Self-medication | -0.16 | 0.56315 | 0.56463 | 0.89583 | 0.89872 |
|  |  | mFTQ | -0.20 | 0.34593 | 0.34607 | 0.83458 | 0.83774 |
|  |  | ICD-10 | -0.16 | 0.40878 | 0.41101 | 0.85250 | 0.85509 |
|  |  | Withdrawal | -0.09 | 0.87679 | 0.87861 | 0.97659 | 0.97697 |
| *CHRNA7* | rs10438342 | No. cigarettes | -0.24 | 0.14339 | 0.14441 | 0.79921 | 0.80409 |
|  |  | ND/craving | -0.15 | 0.74645 | 0.74917 | 0.95566 | 0.95684 |
|  |  | Self-medication | -0.09 | 0.47987 | 0.48322 | 0.87147 | 0.87340 |
|  |  | mFTQ | -0.07 | 0.44330 | 0.44433 | 0.86364 | 0.86705 |
|  |  | ICD-10 | 0.02 | 0.81981 | 0.82108 | 0.96985 | 0.97009 |
|  |  | Withdrawal | 0.00 | 0.98461 | 0.98447 | 0.99655 | 0.99647 |
|  | rs11071512 | No. cigarettes | -0.19 | 0.54863 | 0.55095 | 0.89247 | 0.89469 |
|  |  | ND/craving | -0.39 | 0.66899 | 0.67084 | 0.93275 | 0.93384 |
|  |  | Self-medication | -0.06 | 0.80165 | 0.79993 | 0.96758 | 0.96843 |
|  |  | mFTQ | -0.21 | 0.26608 | 0.26649 | 0.81692 | 0.82073 |
|  |  | ICD-10 | -0.10 | 0.55919 | 0.55907 | 0.89357 | 0.89469 |
|  |  | Withdrawal | -0.33 | 0.50467 | 0.50673 | 0.88796 | 0.89019 |
|  | rs11071530 | No. cigarettes | 0.04 | 0.86414 | 0.86322 | 0.97659 | 0.97697 |
|  |  | ND/craving | -0.19 | 0.78957 | 0.78855 | 0.96758 | 0.96798 |
|  |  | Self-medication | -0.12 | 0.55435 | 0.55538 | 0.89350 | 0.89469 |
|  |  | mFTQ | -0.13 | 0.36901 | 0.37062 | 0.84491 | 0.84876 |
|  |  | ICD-10 | 0.02 | 0.87099 | 0.87233 | 0.97659 | 0.97697 |
|  |  | Withdrawal | -0.06 | 0.88139 | 0.88054 | 0.97659 | 0.97697 |
|  | rs11852956 | No. cigarettes | -0.16 | 0.41043 | 0.41112 | 0.85250 | 0.85509 |
|  |  | ND/craving | 0.02 | 0.97295 | 0.97274 | 0.99655 | 0.99647 |
|  |  | Self-medication | -0.06 | 0.67699 | 0.67814 | 0.93294 | 0.93426 |
|  |  | mFTQ | -0.06 | 0.57017 | 0.57064 | 0.89822 | 0.90234 |
|  |  | ICD-10 | 0.01 | 0.92611 | 0.92651 | 0.98534 | 0.98615 |
|  |  | Withdrawal | 0.10 | 0.74188 | 0.73919 | 0.95449 | 0.95343 |
|  | rs12904458 | No. cigarettes | 0.34 | 0.03251 | 0.03298 | 0.68275 | 0.69122 |
|  |  | ND/craving | 0.59 | 0.18308 | 0.18636 | 0.81687 | 0.82073 |
|  |  | Self-medication | 0.07 | 0.53517 | 0.53603 | 0.89247 | 0.89469 |
|  |  | mFTQ | 0.19 | 0.04141 | 0.04230 | 0.68275 | 0.69122 |
|  |  | ICD-10 | 0.03 | 0.72228 | 0.72347 | 0.94944 | 0.95098 |
|  |  | Withdrawal | 0.14 | 0.54444 | 0.54571 | 0.89247 | 0.89469 |
|  | rs12906868 | No. cigarettes | -0.45 | 0.05835 | 0.06004 | 0.68275 | 0.69122 |
|  |  | ND/craving | -1.15 | 0.08080 | 0.08256 | 0.71335 | 0.72276 |
|  |  | Self-medication | -0.20 | 0.27433 | 0.27663 | 0.81692 | 0.82073 |
|  |  | mFTQ | -0.26 | 0.06049 | 0.06202 | 0.68275 | 0.69122 |
|  |  | ICD-10 | -0.16 | 0.19450 | 0.19685 | 0.81687 | 0.82073 |
|  |  | Withdrawal | -0.46 | 0.20098 | 0.20331 | 0.81687 | 0.82073 |
|  | rs16956223 | No. cigarettes | -0.07 | 0.76740 | 0.76829 | 0.95851 | 0.95961 |
|  |  | ND/craving | 0.32 | 0.62143 | 0.62773 | 0.91735 | 0.92348 |
|  |  | Self-medication | 0.08 | 0.65528 | 0.65691 | 0.92727 | 0.92887 |
|  |  | mFTQ | 0.11 | 0.42136 | 0.42592 | 0.85605 | 0.86008 |
|  |  | ICD-10 | 0.13 | 0.26324 | 0.26609 | 0.81692 | 0.82073 |
|  |  | Withdrawal | 0.22 | 0.51773 | 0.52169 | 0.88888 | 0.89054 |
|  | rs17604861† | No. cigarettes | -0.64 | 0.06319 | 0.06382 | 0.68275 | 0.69122 |
|  |  | ND/craving | -1.42 | 0.13784 | 0.13731 | 0.79750 | 0.79665 |
|  |  | Self-medication | -0.49 | 0.05798 | 0.05742 | 0.68275 | 0.69122 |
|  |  | mFTQ | -0.38 | 0.05304 | 0.05336 | 0.68275 | 0.69122 |
|  |  | ICD-10 | -0.21 | 0.21748 | 0.21569 | 0.81692 | 0.82073 |
|  |  | Withdrawal | -0.70 | 0.17589 | 0.17569 | 0.81239 | 0.81734 |
|  | rs2337980 | No. cigarettes | 0.09 | 0.54930 | 0.54864 | 0.89247 | 0.89469 |
|  |  | ND/craving | 0.07 | 0.87195 | 0.87201 | 0.97659 | 0.97697 |
|  |  | Self-medication | -0.09 | 0.46914 | 0.47134 | 0.87147 | 0.87340 |
|  |  | mFTQ | 0.01 | 0.88514 | 0.88612 | 0.97659 | 0.97697 |
|  |  | ICD-10 | -0.01 | 0.88304 | 0.88430 | 0.97659 | 0.97697 |
|  |  | Withdrawal | -0.21 | 0.39313 | 0.39571 | 0.84836 | 0.85202 |
|  | rs2611603 | No. cigarettes | 0.31 | 0.05896 | 0.06002 | 0.68275 | 0.69122 |
|  |  | ND/craving | 0.92 | 0.04681 | 0.04753 | 0.68275 | 0.69122 |
|  |  | Self-medication | 0.13 | 0.29319 | 0.29404 | 0.81833 | 0.82138 |
|  |  | mFTQ | 0.22 | 0.01919 | 0.01978 | 0.58241 | 0.60471 |
|  |  | ICD-10 | 0.16 | 0.06320 | 0.06361 | 0.68275 | 0.69122 |
|  |  | Withdrawal | 0.47 | 0.06359 | 0.06547 | 0.68275 | 0.69122 |
|  | rs2611605 | No. cigarettes | -0.03 | 0.87478 | 0.87544 | 0.97659 | 0.97697 |
|  |  | ND/craving | 0.19 | 0.73874 | 0.73975 | 0.95259 | 0.95343 |
|  |  | Self-medication | 0.12 | 0.43563 | 0.43608 | 0.85904 | 0.86375 |
|  |  | mFTQ | 0.04 | 0.75115 | 0.75134 | 0.95711 | 0.95769 |
|  |  | ICD-10 | 0.02 | 0.83722 | 0.83838 | 0.97441 | 0.97550 |
|  |  | Withdrawal | 0.14 | 0.65643 | 0.65841 | 0.92757 | 0.92887 |
|  | rs4779565 | No. cigarettes | 0.01 | 0.95161 | 0.95225 | 0.99193 | 0.99216 |
|  |  | ND/craving | 0.10 | 0.82924 | 0.82974 | 0.97146 | 0.97156 |
|  |  | Self-medication | -0.04 | 0.77171 | 0.77268 | 0.96078 | 0.96198 |
|  |  | mFTQ | -0.05 | 0.57527 | 0.57712 | 0.89916 | 0.90267 |
|  |  | ICD-10 | 0.02 | 0.78011 | 0.77914 | 0.96379 | 0.96318 |
|  |  | Withdrawal | 0.09 | 0.70908 | 0.71204 | 0.94589 | 0.94578 |
|  | rs6494212 | No. cigarettes | -0.29 | 0.08638 | 0.08745 | 0.71335 | 0.72276 |
|  |  | ND/craving | -0.65 | 0.16799 | 0.16786 | 0.81132 | 0.81734 |
|  |  | Self-medication | -0.22 | 0.08314 | 0.08389 | 0.71335 | 0.72276 |
|  |  | mFTQ | -0.18 | 0.07087 | 0.07106 | 0.68903 | 0.69122 |
|  |  | ICD-10 | -0.11 | 0.18664 | 0.18740 | 0.81687 | 0.82073 |
|  |  | Withdrawal | -0.23 | 0.36218 | 0.36251 | 0.84491 | 0.84876 |
|  | rs7175581 | No. cigarettes | -0.02 | 0.88444 | 0.88471 | 0.97659 | 0.97697 |
|  |  | ND/craving | -0.45 | 0.30618 | 0.30614 | 0.82443 | 0.82839 |
|  |  | Self-medication | -0.05 | 0.65810 | 0.65901 | 0.92789 | 0.92887 |
|  |  | mFTQ | -0.03 | 0.72757 | 0.72893 | 0.95166 | 0.95174 |
|  |  | ICD-10 | -0.09 | 0.29193 | 0.28872 | 0.81833 | 0.82138 |
|  |  | Withdrawal | -0.21 | 0.37372 | 0.37473 | 0.84491 | 0.84876 |
|  | rs7178176 | No. cigarettes | 0.06 | 0.74516 | 0.74470 | 0.95528 | 0.95552 |
|  |  | ND/craving | -0.06 | 0.90156 | 0.90202 | 0.98061 | 0.98062 |
|  |  | Self-medication | 0.00 | 0.97397 | 0.97408 | 0.99655 | 0.99647 |
|  |  | mFTQ | 0.00 | 0.96452 | 0.96509 | 0.99503 | 0.99559 |
|  |  | ICD-10 | 0.00 | 0.97848 | 0.97844 | 0.99655 | 0.99647 |
|  |  | Withdrawal | 0.18 | 0.50872 | 0.51159 | 0.88826 | 0.89041 |
|  | rs8028396 | No. cigarettes | 0.03 | 0.86107 | 0.86096 | 0.97659 | 0.97697 |
|  |  | ND/craving | -0.20 | 0.67336 | 0.67503 | 0.93294 | 0.93426 |
|  |  | Self-medication | -0.03 | 0.83023 | 0.82981 | 0.97146 | 0.97156 |
|  |  | mFTQ | -0.02 | 0.82960 | 0.82953 | 0.97146 | 0.97156 |
|  |  | ICD-10 | -0.07 | 0.40087 | 0.40161 | 0.85202 | 0.85375 |
|  |  | Withdrawal | -0.16 | 0.52938 | 0.52920 | 0.89247 | 0.89250 |
|  | rs868437 | No. cigarettes | -0.42 | 0.01448 | 0.01584 | 0.57781 | 0.58246 |
|  |  | ND/craving | -1.17 | 0.01596 | 0.01673 | 0.57781 | 0.58246 |
|  |  | Self-medication | -0.23 | 0.08648 | 0.08680 | 0.71335 | 0.72276 |
|  |  | mFTQ | -0.30 | 0.00331 | 0.00352 | 0.35432 | 0.37678 |
|  |  | ICD-10 | -0.17 | 0.05586 | 0.05623 | 0.68275 | 0.69122 |
|  |  | Withdrawal | -0.17 | 0.52691 | 0.52908 | 0.89177 | 0.89250 |
|  | rs885071 | No. cigarettes | -0.43 | 0.02799 | 0.02905 | 0.66747 | 0.69083 |
|  |  | ND/craving | -0.86 | 0.11827 | 0.12017 | 0.74683 | 0.75572 |
|  |  | Self-medication | -0.17 | 0.27156 | 0.27305 | 0.81692 | 0.82073 |
|  |  | mFTQ | -0.23 | 0.04450 | 0.04556 | 0.68275 | 0.69122 |
|  |  | ICD-10 | -0.12 | 0.23170 | 0.23579 | 0.81692 | 0.82073 |
|  |  | Withdrawal | 0.01 | 0.96455 | 0.96428 | 0.99503 | 0.99540 |
| *CHRNB2* | rs1127309 | No. cigarettes | -0.20 | 0.23000 | 0.22861 | 0.81692 | 0.82073 |
|  |  | ND/craving | -0.22 | 0.62679 | 0.62649 | 0.92223 | 0.92348 |
|  |  | Self-medication | 0.06 | 0.64785 | 0.64851 | 0.92516 | 0.92659 |
|  |  | mFTQ | -0.04 | 0.69043 | 0.68986 | 0.93977 | 0.94037 |
|  |  | ICD-10 | 0.01 | 0.92601 | 0.92675 | 0.98534 | 0.98615 |
|  |  | Withdrawal | -0.27 | 0.28222 | 0.28002 | 0.81833 | 0.82073 |
|  | rs2072659 | No. cigarettes | -0.45 | 0.09859 | 0.10100 | 0.71653 | 0.72854 |
|  |  | ND/craving | -1.40 | 0.06747 | 0.07009 | 0.68275 | 0.69122 |
|  |  | Self-medication | -0.39 | 0.06218 | 0.06307 | 0.68275 | 0.69122 |
|  |  | mFTQ | -0.26 | 0.10452 | 0.10834 | 0.73503 | 0.75032 |
|  |  | ICD-10 | -0.24 | 0.08815 | 0.09092 | 0.71335 | 0.72276 |
|  |  | Withdrawal | -0.54 | 0.18882 | 0.19281 | 0.81687 | 0.82073 |
|  | rs2072660 | No. cigarettes | -0.04 | 0.81109 | 0.81084 | 0.96758 | 0.96843 |
|  |  | ND/craving | 0.16 | 0.75568 | 0.75614 | 0.95749 | 0.95874 |
|  |  | Self-medication | 0.11 | 0.40957 | 0.41016 | 0.85250 | 0.85509 |
|  |  | mFTQ | 0.00 | 0.98966 | 0.98969 | 0.99655 | 0.99647 |
|  |  | ICD-10 | 0.02 | 0.80092 | 0.79942 | 0.96758 | 0.96843 |
|  |  | Withdrawal | -0.17 | 0.54638 | 0.54493 | 0.89247 | 0.89469 |
|  | rs3811450 | No. cigarettes | -0.25 | 0.40721 | 0.40747 | 0.85202 | 0.85488 |
|  |  | ND/craving | -0.67 | 0.43818 | 0.44186 | 0.86203 | 0.86665 |
|  |  | Self-medication | -0.05 | 0.83763 | 0.83914 | 0.97441 | 0.97550 |
|  |  | mFTQ | -0.22 | 0.20915 | 0.21149 | 0.81692 | 0.82073 |
|  |  | ICD-10 | -0.12 | 0.43516 | 0.43880 | 0.85904 | 0.86439 |
|  |  | Withdrawal | -0.35 | 0.45540 | 0.45953 | 0.86906 | 0.87224 |
|  | rs4845652 | No. cigarettes | -0.27 | 0.32680 | 0.32577 | 0.82817 | 0.83152 |
|  |  | ND/craving | -0.88 | 0.25182 | 0.25281 | 0.81692 | 0.82073 |
|  |  | Self-medication | -0.18 | 0.38926 | 0.38990 | 0.84836 | 0.85142 |
|  |  | mFTQ | -0.17 | 0.29468 | 0.29802 | 0.81833 | 0.82588 |
|  |  | ICD-10 | -0.14 | 0.31208 | 0.31394 | 0.82443 | 0.82983 |
|  |  | Withdrawal | 0.12 | 0.77392 | 0.77605 | 0.96129 | 0.96227 |
|  | rs9427092 | No. cigarettes | 0.33 | 0.08104 | 0.08082 | 0.71335 | 0.72276 |
|  |  | ND/craving | 0.65 | 0.21713 | 0.21624 | 0.81692 | 0.82073 |
|  |  | Self-medication | 0.11 | 0.45243 | 0.45490 | 0.86906 | 0.87224 |
|  |  | mFTQ | 0.12 | 0.25765 | 0.25748 | 0.81692 | 0.82073 |
|  |  | ICD-10 | 0.10 | 0.29422 | 0.29469 | 0.81833 | 0.82138 |
|  |  | Withdrawal | 0.58 | 0.04130 | 0.04152 | 0.68275 | 0.69122 |
|  | rs9427094 | No. cigarettes | 0.16 | 0.29487 | 0.29305 | 0.81833 | 0.82138 |
|  |  | ND/craving | 0.23 | 0.59384 | 0.59341 | 0.90701 | 0.90779 |
|  |  | Self-medication | 0.00 | 0.96902 | 0.96821 | 0.99655 | 0.99647 |
|  |  | mFTQ | 0.02 | 0.82217 | 0.82221 | 0.96985 | 0.97009 |
|  |  | ICD-10 | 0.01 | 0.91725 | 0.91758 | 0.98436 | 0.98444 |
|  |  | Withdrawal | 0.20 | 0.39408 | 0.39509 | 0.84836 | 0.85202 |
|  | rs9616 | No. cigarettes | -0.04 | 0.82270 | 0.82114 | 0.96985 | 0.97009 |
|  |  | ND/craving | -0.22 | 0.65055 | 0.64930 | 0.92516 | 0.92702 |
|  |  | Self-medication | -0.11 | 0.41076 | 0.41371 | 0.85250 | 0.85586 |
|  |  | mFTQ | -0.06 | 0.57141 | 0.57338 | 0.89822 | 0.90267 |
|  |  | ICD-10 | -0.07 | 0.42037 | 0.42094 | 0.85531 | 0.85791 |
|  |  | Withdrawal | -0.28 | 0.28766 | 0.28890 | 0.81833 | 0.82138 |
| *CHRNB3* | rs6987323 | No. cigarettes | -0.18 | 0.59213 | 0.59462 | 0.90572 | 0.90846 |
|  |  | ND/craving | -0.27 | 0.77887 | 0.77913 | 0.96345 | 0.96318 |
|  |  | Self-medication | 0.03 | 0.91310 | 0.91240 | 0.98436 | 0.98444 |
|  |  | mFTQ | -0.18 | 0.37889 | 0.38319 | 0.84491 | 0.85010 |
|  |  | ICD-10 | -0.07 | 0.69221 | 0.69449 | 0.94086 | 0.94333 |
|  |  | Withdrawal | 0.06 | 0.90818 | 0.90780 | 0.98267 | 0.98226 |
| *CHRNB4* | rs11636605 | No. cigarettes | -0.06 | 0.76662 | 0.76784 | 0.95851 | 0.95961 |
|  |  | ND/craving | -0.46 | 0.41026 | 0.41103 | 0.85250 | 0.85509 |
|  |  | Self-medication | -0.06 | 0.69115 | 0.69210 | 0.94008 | 0.94137 |
|  |  | mFTQ | 0.02 | 0.86388 | 0.86337 | 0.97659 | 0.97697 |
|  |  | ICD-10 | -0.05 | 0.60191 | 0.60525 | 0.91171 | 0.91247 |
|  |  | Withdrawal | -0.21 | 0.49211 | 0.49464 | 0.87760 | 0.88128 |
|  | rs17487223 | No. cigarettes | 0.20 | 0.25204 | 0.25198 | 0.81692 | 0.82073 |
|  |  | ND/craving | 0.40 | 0.39644 | 0.39836 | 0.84836 | 0.85202 |
|  |  | Self-medication | 0.07 | 0.60544 | 0.60547 | 0.91171 | 0.91247 |
|  |  | mFTQ | 0.12 | 0.23148 | 0.23217 | 0.81692 | 0.82073 |
|  |  | ICD-10 | 0.06 | 0.51116 | 0.51469 | 0.88853 | 0.89054 |
|  |  | Withdrawal | 0.19 | 0.47102 | 0.47259 | 0.87147 | 0.87340 |
|  | rs1948 | No. cigarettes | -0.13 | 0.41954 | 0.41779 | 0.85531 | 0.85620 |
|  |  | ND/craving | -0.50 | 0.27250 | 0.27184 | 0.81692 | 0.82073 |
|  |  | Self-medication | -0.13 | 0.30790 | 0.30823 | 0.82443 | 0.82839 |
|  |  | mFTQ | -0.13 | 0.17088 | 0.17107 | 0.81132 | 0.81734 |
|  |  | ICD-10 | -0.08 | 0.33935 | 0.33927 | 0.83024 | 0.83365 |
|  |  | Withdrawal | -0.40 | 0.10148 | 0.10336 | 0.72931 | 0.73783 |
|  | rs6495309 | No. cigarettes | 0.17 | 0.38025 | 0.38246 | 0.84491 | 0.85010 |
|  |  | ND/craving | 0.53 | 0.31092 | 0.31072 | 0.82443 | 0.82983 |
|  |  | Self-medication | 0.19 | 0.19736 | 0.19753 | 0.81687 | 0.82073 |
|  |  | mFTQ | 0.15 | 0.17001 | 0.17237 | 0.81132 | 0.81734 |
|  |  | ICD-10 | 0.15 | 0.11254 | 0.11310 | 0.74516 | 0.75032 |
|  |  | Withdrawal | 0.29 | 0.30744 | 0.30799 | 0.82443 | 0.82839 |
|  | rs950776 | No. cigarettes | -0.16 | 0.33564 | 0.33383 | 0.82975 | 0.83365 |
|  |  | ND/craving | -0.41 | 0.37830 | 0.37600 | 0.84491 | 0.84876 |
|  |  | Self-medication | -0.14 | 0.26559 | 0.26396 | 0.81692 | 0.82073 |
|  |  | mFTQ | -0.12 | 0.19399 | 0.19313 | 0.81687 | 0.82073 |
|  |  | ICD-10 | -0.05 | 0.51463 | 0.51286 | 0.88888 | 0.89054 |
|  |  | Withdrawal | -0.34 | 0.17116 | 0.17143 | 0.81132 | 0.81734 |
| *COMT* | rs165728 | No. cigarettes | -0.15 | 0.62154 | 0.62502 | 0.91735 | 0.92275 |
|  |  | ND/craving | -1.05 | 0.19728 | 0.19724 | 0.81687 | 0.82073 |
|  |  | Self-medication | -0.27 | 0.23373 | 0.23464 | 0.81692 | 0.82073 |
|  |  | mFTQ | -0.19 | 0.25224 | 0.25188 | 0.81692 | 0.82073 |
|  |  | ICD-10 | -0.10 | 0.49643 | 0.49871 | 0.87960 | 0.88283 |
|  |  | Withdrawal | -0.43 | 0.33267 | 0.33448 | 0.82975 | 0.83365 |
|  | rs165774 | No. cigarettes | 0.02 | 0.90183 | 0.90200 | 0.98061 | 0.98062 |
|  |  | ND/craving | 0.32 | 0.50852 | 0.50849 | 0.88826 | 0.89032 |
|  |  | Self-medication | 0.25 | 0.06424 | 0.06495 | 0.68275 | 0.69122 |
|  |  | mFTQ | 0.05 | 0.60539 | 0.60422 | 0.91171 | 0.91247 |
|  |  | ICD-10 | 0.05 | 0.57661 | 0.57748 | 0.89923 | 0.90267 |
|  |  | Withdrawal | 0.30 | 0.26078 | 0.26272 | 0.81692 | 0.82073 |
|  | rs165824 | No. cigarettes | -0.19 | 0.43392 | 0.43682 | 0.85904 | 0.86375 |
|  |  | ND/craving | -1.11 | 0.09221 | 0.09154 | 0.71653 | 0.72276 |
|  |  | Self-medication | -0.35 | 0.05447 | 0.05397 | 0.68275 | 0.69122 |
|  |  | mFTQ | -0.22 | 0.11866 | 0.11707 | 0.74688 | 0.75032 |
|  |  | ICD-10 | -0.12 | 0.31056 | 0.31121 | 0.82443 | 0.82983 |
|  |  | Withdrawal | -0.68 | 0.06165 | 0.06087 | 0.68275 | 0.69122 |
|  | rs16982844† | No. cigarettes | 0.03 | 0.93424 | 0.93397 | 0.98732 | 0.98766 |
|  |  | ND/craving | -0.33 | 0.74314 | 0.74230 | 0.95464 | 0.95502 |
|  |  | Self-medication | 0.13 | 0.65006 | 0.65069 | 0.92516 | 0.92763 |
|  |  | mFTQ | 0.00 | 0.99670 | 0.99624 | 0.99828 | 0.99812 |
|  |  | ICD-10 | -0.09 | 0.64561 | 0.64540 | 0.92403 | 0.92557 |
|  |  | Withdrawal | 0.20 | 0.71135 | 0.71118 | 0.94689 | 0.94578 |
|  | rs174696 | No. cigarettes | -0.22 | 0.20106 | 0.20315 | 0.81687 | 0.82073 |
|  |  | ND/craving | -1.17 | 0.01685 | 0.01642 | 0.57781 | 0.58246 |
|  |  | Self-medication | -0.36 | 0.00734 | 0.00763 | 0.50491 | 0.52493 |
|  |  | mFTQ | -0.15 | 0.14855 | 0.15066 | 0.80364 | 0.80972 |
|  |  | ICD-10 | -0.15 | 0.09843 | 0.09943 | 0.71653 | 0.72775 |
|  |  | Withdrawal | -0.46 | 0.08293 | 0.08452 | 0.71335 | 0.72276 |
|  | rs2020917 | No. cigarettes | 0.44 | 0.01770 | 0.01813 | 0.57781 | 0.58246 |
|  |  | ND/craving | 1.39 | 0.00681 | 0.00647 | 0.49934 | 0.49807 |
|  |  | Self-medication | 0.32 | 0.02148 | 0.02181 | 0.61858 | 0.61765 |
|  |  | mFTQ | 0.23 | 0.03466 | 0.03535 | 0.68275 | 0.69122 |
|  |  | ICD-10 | 0.24 | 0.01108 | 0.01106 | 0.52414 | 0.53402 |
|  |  | Withdrawal | 0.58 | 0.03770 | 0.03743 | 0.68275 | 0.69122 |
|  | rs2239395 | No. cigarettes | -0.58 | 0.03367 | 0.03274 | 0.68275 | 0.69122 |
|  |  | ND/craving | -1.79 | 0.02207 | 0.02130 | 0.61858 | 0.61765 |
|  |  | Self-medication | -0.53 | 0.01285 | 0.01278 | 0.53798 | 0.54712 |
|  |  | mFTQ | -0.40 | 0.01415 | 0.01412 | 0.57781 | 0.57858 |
|  |  | ICD-10 | -0.33 | 0.02251 | 0.02221 | 0.61858 | 0.62008 |
|  |  | Withdrawal | -0.61 | 0.15239 | 0.15025 | 0.81063 | 0.80972 |
|  | rs4646310 | No. cigarettes | -0.41 | 0.03917 | 0.04081 | 0.68275 | 0.69122 |
|  |  | ND/craving | -0.80 | 0.15429 | 0.15433 | 0.81063 | 0.81147 |
|  |  | Self-medication | -0.11 | 0.45537 | 0.45644 | 0.86906 | 0.87224 |
|  |  | mFTQ | -0.12 | 0.29832 | 0.30074 | 0.82435 | 0.82839 |
|  |  | ICD-10 | -0.13 | 0.19822 | 0.19930 | 0.81687 | 0.82073 |
|  |  | Withdrawal | -0.23 | 0.45916 | 0.46020 | 0.86906 | 0.87224 |
|  | rs4646316 | No. cigarettes | 0.06 | 0.75330 | 0.75495 | 0.95749 | 0.95849 |
|  |  | ND/craving | 0.35 | 0.51079 | 0.51110 | 0.88853 | 0.89041 |
|  |  | Self-medication | 0.12 | 0.39577 | 0.39751 | 0.84836 | 0.85202 |
|  |  | mFTQ | 0.02 | 0.86623 | 0.86720 | 0.97659 | 0.97697 |
|  |  | ICD-10 | 0.03 | 0.76007 | 0.75990 | 0.95749 | 0.95943 |
|  |  | Withdrawal | 0.22 | 0.43473 | 0.43580 | 0.85904 | 0.86375 |
|  | rs4680 | No. cigarettes | -0.01 | 0.95792 | 0.95726 | 0.99406 | 0.99443 |
|  |  | ND/craving | 0.09 | 0.83693 | 0.83925 | 0.97441 | 0.97550 |
|  |  | Self-medication | 0.08 | 0.50191 | 0.50239 | 0.88686 | 0.88771 |
|  |  | mFTQ | 0.08 | 0.37681 | 0.37814 | 0.84491 | 0.84876 |
|  |  | ICD-10 | 0.00 | 0.96329 | 0.96353 | 0.99503 | 0.99540 |
|  |  | Withdrawal | -0.05 | 0.84573 | 0.84726 | 0.97554 | 0.97697 |
|  | rs5746848 | No. cigarettes | -0.04 | 0.80558 | 0.80464 | 0.96758 | 0.96843 |
|  |  | ND/craving | -0.27 | 0.54281 | 0.54468 | 0.89247 | 0.89469 |
|  |  | Self-medication | -0.04 | 0.75649 | 0.75940 | 0.95749 | 0.95943 |
|  |  | mFTQ | -0.04 | 0.66863 | 0.66960 | 0.93275 | 0.93384 |
|  |  | ICD-10 | -0.07 | 0.38028 | 0.38400 | 0.84491 | 0.85010 |
|  |  | Withdrawal | -0.26 | 0.27851 | 0.28422 | 0.81692 | 0.82138 |
|  | rs740601 | No. cigarettes | 0.17 | 0.29277 | 0.29573 | 0.81833 | 0.82308 |
|  |  | ND/craving | 0.62 | 0.17439 | 0.17683 | 0.81132 | 0.81790 |
|  |  | Self-medication | 0.17 | 0.17189 | 0.17360 | 0.81132 | 0.81734 |
|  |  | mFTQ | 0.07 | 0.48310 | 0.48315 | 0.87147 | 0.87340 |
|  |  | ICD-10 | 0.10 | 0.23158 | 0.23389 | 0.81692 | 0.82073 |
|  |  | Withdrawal | 0.29 | 0.23541 | 0.23938 | 0.81692 | 0.82073 |
|  | rs740603 | No. cigarettes | 0.08 | 0.60008 | 0.60246 | 0.91171 | 0.91247 |
|  |  | ND/craving | 0.24 | 0.57473 | 0.57608 | 0.89916 | 0.90267 |
|  |  | Self-medication | 0.02 | 0.89205 | 0.89293 | 0.97785 | 0.97873 |
|  |  | mFTQ | 0.11 | 0.23551 | 0.23764 | 0.81692 | 0.82073 |
|  |  | ICD-10 | 0.06 | 0.42201 | 0.42355 | 0.85648 | 0.86008 |
|  |  | Withdrawal | 0.03 | 0.89404 | 0.89335 | 0.97915 | 0.97873 |
|  | rs887200 | No. cigarettes | -0.53 | 0.01123 | 0.01192 | 0.52414 | 0.53402 |
|  |  | ND/craving | -2.08 | 0.00038 | 0.00045 | 0.12423 | 0.12539 |
|  |  | Self-medication | -0.58 | 0.00031 | 0.00040 | 0.12423 | 0.12539 |
|  |  | mFTQ | -0.42 | 0.00052 | 0.00050 | 0.12423 | 0.12539 |
|  |  | ICD-10 | -0.33 | 0.00211 | 0.00204 | 0.23925 | 0.23090 |
|  |  | Withdrawal | -0.75 | 0.01935 | 0.02010 | 0.58241 | 0.60481 |
|  | rs9265 | No. cigarettes | -0.16 | 0.32592 | 0.32485 | 0.82813 | 0.83152 |
|  |  | ND/craving | -0.55 | 0.22603 | 0.22599 | 0.81692 | 0.82073 |
|  |  | Self-medication | -0.24 | 0.04813 | 0.04848 | 0.68275 | 0.69122 |
|  |  | mFTQ | -0.15 | 0.10662 | 0.10661 | 0.73868 | 0.74810 |
|  |  | ICD-10 | -0.08 | 0.33256 | 0.33366 | 0.82975 | 0.83365 |
|  |  | Withdrawal | -0.40 | 0.10134 | 0.10051 | 0.72931 | 0.72775 |
|  | rs9306235 | No. cigarettes | 0.26 | 0.41276 | 0.41282 | 0.85327 | 0.85586 |
|  |  | ND/craving | 1.27 | 0.15522 | 0.15798 | 0.81063 | 0.81586 |
|  |  | Self-medication | 0.32 | 0.18967 | 0.19172 | 0.81687 | 0.82073 |
|  |  | mFTQ | 0.24 | 0.19110 | 0.19212 | 0.81687 | 0.82073 |
|  |  | ICD-10 | 0.28 | 0.08865 | 0.09001 | 0.71440 | 0.72276 |
|  |  | Withdrawal | 0.95 | 0.05012 | 0.05026 | 0.68275 | 0.69122 |
|  | rs9332377 | No. cigarettes | 0.14 | 0.51674 | 0.51839 | 0.88888 | 0.89054 |
|  |  | ND/craving | 0.53 | 0.38443 | 0.38651 | 0.84811 | 0.85020 |
|  |  | Self-medication | 0.08 | 0.61176 | 0.61024 | 0.91338 | 0.91476 |
|  |  | mFTQ | 0.04 | 0.75812 | 0.75844 | 0.95749 | 0.95912 |
|  |  | ICD-10 | 0.08 | 0.46467 | 0.46662 | 0.87147 | 0.87340 |
|  |  | Withdrawal | 0.16 | 0.63420 | 0.63184 | 0.92368 | 0.92547 |
|  | rs9605030 | No. cigarettes | 0.44 | 0.07428 | 0.07548 | 0.70147 | 0.70910 |
|  |  | ND/craving | 1.53 | 0.02799 | 0.02857 | 0.66747 | 0.69083 |
|  |  | Self-medication | 0.36 | 0.05784 | 0.05708 | 0.68275 | 0.69122 |
|  |  | mFTQ | 0.25 | 0.08788 | 0.08956 | 0.71335 | 0.72276 |
|  |  | ICD-10 | 0.18 | 0.15923 | 0.16145 | 0.81074 | 0.81586 |
|  |  | Withdrawal | 0.66 | 0.07920 | 0.08002 | 0.71335 | 0.72276 |
|  | rs9617850 | No. cigarettes | -0.30 | 0.12288 | 0.12426 | 0.76588 | 0.77377 |
|  |  | ND/craving | -0.95 | 0.08140 | 0.08251 | 0.71335 | 0.72276 |
|  |  | Self-medication | -0.14 | 0.34577 | 0.34808 | 0.83458 | 0.83895 |
|  |  | mFTQ | -0.14 | 0.22932 | 0.23225 | 0.81692 | 0.82073 |
|  |  | ICD-10 | -0.18 | 0.06058 | 0.06205 | 0.68275 | 0.69122 |
|  |  | Withdrawal | -0.22 | 0.45137 | 0.45269 | 0.86906 | 0.87224 |
|  | rs8140265 | No. cigarettes | 0.44 | 0.01658 | 0.01695 | 0.57781 | 0.58246 |
|  |  | ND/craving | 1.41 | 0.00633 | 0.00626 | 0.48756 | 0.49807 |
|  |  | Self-medication | 0.31 | 0.02591 | 0.02574 | 0.64414 | 0.65220 |
|  |  | mFTQ | 0.23 | 0.03275 | 0.03316 | 0.68275 | 0.69122 |
|  |  | ICD-10 | 0.24 | 0.01100 | 0.01104 | 0.52414 | 0.53402 |
|  |  | Withdrawal | 0.56 | 0.04569 | 0.04599 | 0.68275 | 0.69122 |
| *DBH* | rs1029372 | No. cigarettes | -0.06 | 0.71978 | 0.72043 | 0.94944 | 0.95098 |
|  |  | ND/craving | -0.36 | 0.41012 | 0.40928 | 0.85250 | 0.85497 |
|  |  | Self-medication | -0.16 | 0.17374 | 0.17509 | 0.81132 | 0.81734 |
|  |  | mFTQ | -0.08 | 0.40723 | 0.40703 | 0.85202 | 0.85488 |
|  |  | ICD-10 | -0.07 | 0.38160 | 0.38143 | 0.84553 | 0.84929 |
|  |  | Withdrawal | -0.14 | 0.54848 | 0.54752 | 0.89247 | 0.89469 |
|  | rs1076150 | No. cigarettes | -0.23 | 0.14745 | 0.14560 | 0.80222 | 0.80409 |
|  |  | ND/craving | -0.69 | 0.12483 | 0.12494 | 0.76898 | 0.77377 |
|  |  | Self-medication | -0.16 | 0.19150 | 0.19059 | 0.81687 | 0.82073 |
|  |  | mFTQ | -0.17 | 0.07063 | 0.07007 | 0.68903 | 0.69122 |
|  |  | ICD-10 | -0.12 | 0.13161 | 0.13209 | 0.78234 | 0.78762 |
|  |  | Withdrawal | -0.18 | 0.45030 | 0.45217 | 0.86906 | 0.87224 |
|  | rs1076153 | No. cigarettes | -0.20 | 0.31276 | 0.31392 | 0.82443 | 0.82983 |
|  |  | ND/craving | -0.38 | 0.49151 | 0.49279 | 0.87733 | 0.88043 |
|  |  | Self-medication | -0.10 | 0.49084 | 0.49146 | 0.87733 | 0.87970 |
|  |  | mFTQ | -0.04 | 0.71276 | 0.71174 | 0.94741 | 0.94578 |
|  |  | ICD-10 | -0.09 | 0.37991 | 0.38015 | 0.84491 | 0.84876 |
|  |  | Withdrawal | -0.05 | 0.87180 | 0.87252 | 0.97659 | 0.97697 |
|  | rs10993874 | No. cigarettes | -0.30 | 0.18375 | 0.18417 | 0.81687 | 0.82073 |
|  |  | ND/craving | -0.93 | 0.13557 | 0.13755 | 0.79126 | 0.79665 |
|  |  | Self-medication | -0.24 | 0.15807 | 0.16048 | 0.81063 | 0.81586 |
|  |  | mFTQ | -0.18 | 0.17650 | 0.17757 | 0.81324 | 0.81818 |
|  |  | ICD-10 | -0.17 | 0.12984 | 0.13139 | 0.77907 | 0.78587 |
|  |  | Withdrawal | -0.49 | 0.14918 | 0.15093 | 0.80364 | 0.80972 |
|  | rs10993947 | No. cigarettes | -0.07 | 0.81709 | 0.81809 | 0.96885 | 0.97009 |
|  |  | ND/craving | -0.33 | 0.69628 | 0.69605 | 0.94166 | 0.94333 |
|  |  | Self-medication | -0.13 | 0.57154 | 0.57462 | 0.89822 | 0.90267 |
|  |  | mFTQ | 0.02 | 0.92925 | 0.92922 | 0.98716 | 0.98714 |
|  |  | ICD-10 | 0.01 | 0.96372 | 0.96439 | 0.99503 | 0.99540 |
|  |  | Withdrawal | -0.72 | 0.11975 | 0.12046 | 0.75128 | 0.75572 |
|  | rs1108580 | No. cigarettes | -0.27 | 0.08589 | 0.08447 | 0.71335 | 0.72276 |
|  |  | ND/craving | -0.80 | 0.07355 | 0.07325 | 0.70129 | 0.70731 |
|  |  | Self-medication | -0.20 | 0.10907 | 0.11014 | 0.74516 | 0.75032 |
|  |  | mFTQ | -0.18 | 0.05385 | 0.05387 | 0.68275 | 0.69122 |
|  |  | ICD-10 | -0.13 | 0.11091 | 0.11087 | 0.74516 | 0.75032 |
|  |  | Withdrawal | -0.22 | 0.36831 | 0.36761 | 0.84491 | 0.84876 |
|  | rs1108581 | No. cigarettes | 0.17 | 0.37156 | 0.37458 | 0.84491 | 0.84876 |
|  |  | ND/craving | 0.16 | 0.76304 | 0.76258 | 0.95755 | 0.95944 |
|  |  | Self-medication | 0.02 | 0.87794 | 0.87776 | 0.97659 | 0.97697 |
|  |  | mFTQ | 0.02 | 0.88945 | 0.89023 | 0.97659 | 0.97697 |
|  |  | ICD-10 | 0.03 | 0.79605 | 0.79591 | 0.96758 | 0.96843 |
|  |  | Withdrawal | -0.25 | 0.39374 | 0.39461 | 0.84836 | 0.85202 |
|  | rs129882 | No. cigarettes | -0.19 | 0.32413 | 0.32520 | 0.82813 | 0.83152 |
|  |  | ND/craving | -0.76 | 0.16988 | 0.17168 | 0.81132 | 0.81734 |
|  |  | Self-medication | -0.19 | 0.20090 | 0.20266 | 0.81687 | 0.82073 |
|  |  | mFTQ | -0.11 | 0.35567 | 0.35654 | 0.84407 | 0.84778 |
|  |  | ICD-10 | -0.12 | 0.24413 | 0.24569 | 0.81692 | 0.82073 |
|  |  | Withdrawal | -0.50 | 0.09751 | 0.09829 | 0.71653 | 0.72775 |
|  | rs13306304 | No. cigarettes | 0.20 | 0.45781 | 0.45753 | 0.86906 | 0.87224 |
|  |  | ND/craving | 0.34 | 0.65246 | 0.65190 | 0.92536 | 0.92773 |
|  |  | Self-medication | 0.10 | 0.61340 | 0.61145 | 0.91370 | 0.91476 |
|  |  | mFTQ | 0.12 | 0.45376 | 0.45093 | 0.86906 | 0.87224 |
|  |  | ICD-10 | 0.14 | 0.30875 | 0.30986 | 0.82443 | 0.82927 |
|  |  | Withdrawal | -0.11 | 0.78272 | 0.78228 | 0.96451 | 0.96520 |
|  | rs1541332 | No. cigarettes | -0.18 | 0.25819 | 0.25592 | 0.81692 | 0.82073 |
|  |  | ND/craving | -0.59 | 0.18862 | 0.18614 | 0.81687 | 0.82073 |
|  |  | Self-medication | -0.16 | 0.19428 | 0.19244 | 0.81687 | 0.82073 |
|  |  | mFTQ | -0.18 | 0.05619 | 0.05682 | 0.68275 | 0.69122 |
|  |  | ICD-10 | -0.07 | 0.36892 | 0.36786 | 0.84491 | 0.84876 |
|  |  | Withdrawal | -0.11 | 0.64505 | 0.64505 | 0.92403 | 0.92557 |
|  | rs1611114 | No. cigarettes | -0.19 | 0.29354 | 0.29300 | 0.81833 | 0.82138 |
|  |  | ND/craving | -0.52 | 0.30515 | 0.30650 | 0.82443 | 0.82839 |
|  |  | Self-medication | -0.12 | 0.40005 | 0.40359 | 0.85202 | 0.85488 |
|  |  | mFTQ | -0.11 | 0.27468 | 0.27648 | 0.81692 | 0.82073 |
|  |  | ICD-10 | -0.12 | 0.19258 | 0.19309 | 0.81687 | 0.82073 |
|  |  | Withdrawal | -0.20 | 0.47135 | 0.47321 | 0.87147 | 0.87340 |
|  | rs1611115 | No. cigarettes | -0.06 | 0.75732 | 0.75558 | 0.95749 | 0.95866 |
|  |  | ND/craving | -0.20 | 0.69845 | 0.69759 | 0.94166 | 0.94333 |
|  |  | Self-medication | -0.03 | 0.81329 | 0.81414 | 0.96846 | 0.96912 |
|  |  | mFTQ | -0.09 | 0.39687 | 0.39806 | 0.84836 | 0.85202 |
|  |  | ICD-10 | 0.01 | 0.94262 | 0.94237 | 0.98879 | 0.98910 |
|  |  | Withdrawal | 0.06 | 0.84000 | 0.84090 | 0.97519 | 0.97564 |
|  | rs1611120 | No. cigarettes | -0.09 | 0.79336 | 0.79407 | 0.96758 | 0.96843 |
|  |  | ND/craving | -0.18 | 0.84523 | 0.84510 | 0.97554 | 0.97638 |
|  |  | Self-medication | -0.01 | 0.98008 | 0.98045 | 0.99655 | 0.99647 |
|  |  | mFTQ | -0.15 | 0.42055 | 0.42166 | 0.85531 | 0.85847 |
|  |  | ICD-10 | -0.04 | 0.82978 | 0.83083 | 0.97146 | 0.97182 |
|  |  | Withdrawal | -0.40 | 0.41996 | 0.42088 | 0.85531 | 0.85791 |
|  | rs1611124 | No. cigarettes | 0.22 | 0.51957 | 0.51885 | 0.88888 | 0.89054 |
|  |  | ND/craving | 0.34 | 0.71040 | 0.70774 | 0.94687 | 0.94578 |
|  |  | Self-medication | 0.03 | 0.91675 | 0.91808 | 0.98436 | 0.98444 |
|  |  | mFTQ | 0.21 | 0.27203 | 0.27111 | 0.81692 | 0.82073 |
|  |  | ICD-10 | 0.11 | 0.50476 | 0.50292 | 0.88796 | 0.88783 |
|  |  | Withdrawal | -0.46 | 0.36465 | 0.36313 | 0.84491 | 0.84876 |
|  | rs17150735 | No. cigarettes | 0.58 | 0.05323 | 0.05457 | 0.68275 | 0.69122 |
|  |  | ND/craving | 0.83 | 0.31766 | 0.31983 | 0.82728 | 0.83152 |
|  |  | Self-medication | 0.03 | 0.88267 | 0.88545 | 0.97659 | 0.97697 |
|  |  | mFTQ | 0.17 | 0.31794 | 0.32031 | 0.82728 | 0.83152 |
|  |  | ICD-10 | 0.02 | 0.89895 | 0.89990 | 0.98061 | 0.98062 |
|  |  | Withdrawal | -0.11 | 0.79870 | 0.80206 | 0.96758 | 0.96843 |
|  | rs2007153 | No. cigarettes | 0.08 | 0.62093 | 0.62099 | 0.91735 | 0.91938 |
|  |  | ND/craving | 0.06 | 0.89648 | 0.89743 | 0.97915 | 0.98054 |
|  |  | Self-medication | 0.01 | 0.90066 | 0.90049 | 0.98061 | 0.98062 |
|  |  | mFTQ | -0.01 | 0.88570 | 0.88675 | 0.97659 | 0.97697 |
|  |  | ICD-10 | 0.03 | 0.69823 | 0.69880 | 0.94166 | 0.94333 |
|  |  | Withdrawal | -0.06 | 0.81358 | 0.81358 | 0.96846 | 0.96905 |
|  | rs2073837 | No. cigarettes | -0.03 | 0.87182 | 0.87164 | 0.97659 | 0.97697 |
|  |  | ND/craving | -0.46 | 0.31249 | 0.30956 | 0.82443 | 0.82927 |
|  |  | Self-medication | -0.11 | 0.39462 | 0.39412 | 0.84836 | 0.85202 |
|  |  | mFTQ | -0.14 | 0.14371 | 0.14317 | 0.79921 | 0.80409 |
|  |  | ICD-10 | -0.11 | 0.17392 | 0.17403 | 0.81132 | 0.81734 |
|  |  | Withdrawal | -0.23 | 0.35557 | 0.35406 | 0.84407 | 0.84397 |
|  | rs2097628 | No. cigarettes | 0.14 | 0.39312 | 0.39567 | 0.84836 | 0.85202 |
|  |  | ND/craving | -0.06 | 0.89533 | 0.89500 | 0.97915 | 0.97997 |
|  |  | Self-medication | 0.02 | 0.85389 | 0.85713 | 0.97659 | 0.97697 |
|  |  | mFTQ | -0.02 | 0.79608 | 0.79565 | 0.96758 | 0.96843 |
|  |  | ICD-10 | 0.00 | 0.97307 | 0.97416 | 0.99655 | 0.99647 |
|  |  | Withdrawal | -0.15 | 0.54956 | 0.55302 | 0.89247 | 0.89469 |
|  | rs2283123 | No. cigarettes | 0.25 | 0.32386 | 0.32429 | 0.82813 | 0.83152 |
|  |  | ND/craving | 0.52 | 0.46070 | 0.46128 | 0.86906 | 0.87224 |
|  |  | Self-medication | 0.10 | 0.58653 | 0.59056 | 0.90520 | 0.90738 |
|  |  | mFTQ | 0.17 | 0.24689 | 0.24649 | 0.81692 | 0.82073 |
|  |  | ICD-10 | 0.16 | 0.20401 | 0.20359 | 0.81687 | 0.82073 |
|  |  | Withdrawal | 0.01 | 0.98805 | 0.98794 | 0.99655 | 0.99647 |
|  | rs2283124 | No. cigarettes | 0.23 | 0.33511 | 0.33588 | 0.82975 | 0.83365 |
|  |  | ND/craving | 0.54 | 0.41378 | 0.41349 | 0.85330 | 0.85586 |
|  |  | Self-medication | 0.10 | 0.60155 | 0.60499 | 0.91171 | 0.91247 |
|  |  | mFTQ | 0.18 | 0.20563 | 0.20551 | 0.81692 | 0.82073 |
|  |  | ICD-10 | 0.17 | 0.15868 | 0.15750 | 0.81063 | 0.81586 |
|  |  | Withdrawal | -0.09 | 0.79732 | 0.79834 | 0.96758 | 0.96843 |
|  | rs251914 | No. cigarettes | 0.25 | 0.13954 | 0.14153 | 0.79750 | 0.80409 |
|  |  | ND/craving | 0.75 | 0.11131 | 0.11241 | 0.74516 | 0.75032 |
|  |  | Self-medication | 0.24 | 0.05796 | 0.05891 | 0.68275 | 0.69122 |
|  |  | mFTQ | 0.16 | 0.11186 | 0.11276 | 0.74516 | 0.75032 |
|  |  | ICD-10 | 0.14 | 0.09593 | 0.09624 | 0.71653 | 0.72775 |
|  |  | Withdrawal | 0.53 | 0.03856 | 0.03868 | 0.68275 | 0.69122 |
|  | rs2519143 | No. cigarettes | -0.04 | 0.84910 | 0.84808 | 0.97659 | 0.97697 |
|  |  | ND/craving | -0.14 | 0.79141 | 0.79330 | 0.96758 | 0.96843 |
|  |  | Self-medication | -0.03 | 0.82328 | 0.82219 | 0.96985 | 0.97009 |
|  |  | mFTQ | -0.07 | 0.54462 | 0.54759 | 0.89247 | 0.89469 |
|  |  | ICD-10 | 0.03 | 0.77574 | 0.77713 | 0.96142 | 0.96227 |
|  |  | Withdrawal | 0.07 | 0.80088 | 0.80110 | 0.96758 | 0.96843 |
|  | rs2519148 | No. cigarettes | 0.03 | 0.86097 | 0.85986 | 0.97659 | 0.97697 |
|  |  | ND/craving | 0.10 | 0.81959 | 0.82044 | 0.96985 | 0.97009 |
|  |  | Self-medication | 0.00 | 0.98633 | 0.98610 | 0.99655 | 0.99647 |
|  |  | mFTQ | 0.02 | 0.84076 | 0.84085 | 0.97548 | 0.97564 |
|  |  | ICD-10 | 0.01 | 0.88009 | 0.88294 | 0.97659 | 0.97697 |
|  |  | Withdrawal | -0.08 | 0.73152 | 0.73111 | 0.95166 | 0.95174 |
|  | rs2519154 | No. cigarettes | -0.30 | 0.05238 | 0.05242 | 0.68275 | 0.69122 |
|  |  | ND/craving | -1.01 | 0.02062 | 0.02081 | 0.61098 | 0.61664 |
|  |  | Self-medication | -0.28 | 0.01686 | 0.01761 | 0.57781 | 0.58246 |
|  |  | mFTQ | -0.21 | 0.01739 | 0.01767 | 0.57781 | 0.58246 |
|  |  | ICD-10 | -0.17 | 0.03063 | 0.03057 | 0.68275 | 0.69122 |
|  |  | Withdrawal | -0.38 | 0.10302 | 0.10409 | 0.73197 | 0.73783 |
|  | rs2797849 | No. cigarettes | 0.11 | 0.52930 | 0.52828 | 0.89247 | 0.89250 |
|  |  | ND/craving | 0.53 | 0.26106 | 0.26061 | 0.81692 | 0.82073 |
|  |  | Self-medication | 0.15 | 0.23705 | 0.23563 | 0.81692 | 0.82073 |
|  |  | mFTQ | 0.14 | 0.15578 | 0.15429 | 0.81063 | 0.81147 |
|  |  | ICD-10 | 0.10 | 0.24457 | 0.24569 | 0.81692 | 0.82073 |
|  |  | Withdrawal | 0.36 | 0.15834 | 0.15883 | 0.81063 | 0.81586 |
|  | rs2797853 | No. cigarettes | 0.31 | 0.06405 | 0.06402 | 0.68275 | 0.69122 |
|  |  | ND/craving | 0.99 | 0.03586 | 0.03584 | 0.68275 | 0.69122 |
|  |  | Self-medication | 0.24 | 0.06136 | 0.06068 | 0.68275 | 0.69122 |
|  |  | mFTQ | 0.22 | 0.02318 | 0.02344 | 0.61858 | 0.62356 |
|  |  | ICD-10 | 0.15 | 0.08018 | 0.08047 | 0.71335 | 0.72276 |
|  |  | Withdrawal | 0.64 | 0.01259 | 0.01230 | 0.53798 | 0.53824 |
|  | rs2797855 | No. cigarettes | 0.13 | 0.43445 | 0.43490 | 0.85904 | 0.86375 |
|  |  | ND/craving | 0.60 | 0.18715 | 0.18756 | 0.81687 | 0.82073 |
|  |  | Self-medication | 0.12 | 0.32283 | 0.32276 | 0.82813 | 0.83152 |
|  |  | mFTQ | 0.18 | 0.06043 | 0.06192 | 0.68275 | 0.69122 |
|  |  | ICD-10 | 0.12 | 0.14397 | 0.14498 | 0.79921 | 0.80409 |
|  |  | Withdrawal | 0.16 | 0.52244 | 0.52146 | 0.88888 | 0.89054 |
|  | rs3025343† | No. cigarettes | 0.31 | 0.25869 | 0.25898 | 0.81692 | 0.82073 |
|  |  | ND/craving | 0.73 | 0.34523 | 0.34588 | 0.83458 | 0.83774 |
|  |  | Self-medication | 0.19 | 0.37836 | 0.37916 | 0.84491 | 0.84876 |
|  |  | mFTQ | 0.10 | 0.52805 | 0.52734 | 0.89213 | 0.89172 |
|  |  | ICD-10 | 0.02 | 0.87971 | 0.88005 | 0.97659 | 0.97697 |
|  |  | Withdrawal | 0.40 | 0.33566 | 0.33892 | 0.82975 | 0.83365 |
|  | rs3025355 | No. cigarettes | 0.62 | 0.08241 | 0.08387 | 0.71335 | 0.72276 |
|  |  | ND/craving | 1.62 | 0.11188 | 0.11493 | 0.74516 | 0.75032 |
|  |  | Self-medication | 0.25 | 0.37240 | 0.37548 | 0.84491 | 0.84876 |
|  |  | mFTQ | 0.36 | 0.08937 | 0.09039 | 0.71653 | 0.72276 |
|  |  | ICD-10 | 0.14 | 0.44074 | 0.44427 | 0.86332 | 0.86705 |
|  |  | Withdrawal | 0.79 | 0.14980 | 0.15283 | 0.80364 | 0.81147 |
|  | rs3025365 | No. cigarettes | -0.36 | 0.12444 | 0.12559 | 0.76898 | 0.77527 |
|  |  | ND/craving | -0.88 | 0.17996 | 0.18043 | 0.81687 | 0.82073 |
|  |  | Self-medication | -0.21 | 0.25034 | 0.25055 | 0.81692 | 0.82073 |
|  |  | mFTQ | -0.18 | 0.17939 | 0.18016 | 0.81687 | 0.82073 |
|  |  | ICD-10 | -0.10 | 0.42577 | 0.42677 | 0.85803 | 0.86008 |
|  |  | Withdrawal | -0.34 | 0.34590 | 0.34613 | 0.83458 | 0.83774 |
|  | rs3025369 | No. cigarettes | -0.19 | 0.54148 | 0.54493 | 0.89247 | 0.89469 |
|  |  | ND/craving | -0.73 | 0.40288 | 0.40583 | 0.85202 | 0.85488 |
|  |  | Self-medication | -0.51 | 0.03112 | 0.03154 | 0.68275 | 0.69122 |
|  |  | mFTQ | -0.18 | 0.33257 | 0.33571 | 0.82975 | 0.83365 |
|  |  | ICD-10 | -0.16 | 0.30535 | 0.30777 | 0.82443 | 0.82839 |
|  |  | Withdrawal | -0.29 | 0.54306 | 0.54548 | 0.89247 | 0.89469 |
|  | rs3025373 | No. cigarettes | 0.05 | 0.82295 | 0.82346 | 0.96985 | 0.97009 |
|  |  | ND/craving | 0.25 | 0.70250 | 0.70318 | 0.94352 | 0.94515 |
|  |  | Self-medication | -0.05 | 0.77623 | 0.77653 | 0.96142 | 0.96227 |
|  |  | mFTQ | 0.06 | 0.63757 | 0.63907 | 0.92368 | 0.92547 |
|  |  | ICD-10 | 0.00 | 0.98788 | 0.98750 | 0.99655 | 0.99647 |
|  |  | Withdrawal | -0.05 | 0.88623 | 0.88516 | 0.97659 | 0.97697 |
|  | rs3025382 | No. cigarettes | 0.24 | 0.33400 | 0.33306 | 0.82975 | 0.83365 |
|  |  | ND/craving | 0.20 | 0.77838 | 0.77661 | 0.96345 | 0.96227 |
|  |  | Self-medication | 0.04 | 0.85096 | 0.85356 | 0.97659 | 0.97697 |
|  |  | mFTQ | 0.09 | 0.51573 | 0.51415 | 0.88888 | 0.89054 |
|  |  | ICD-10 | 0.04 | 0.73333 | 0.73447 | 0.95166 | 0.95258 |
|  |  | Withdrawal | -0.30 | 0.41983 | 0.41935 | 0.85531 | 0.85695 |
|  | rs3025388 | No. cigarettes | 0.24 | 0.24667 | 0.24793 | 0.81692 | 0.82073 |
|  |  | ND/craving | 0.41 | 0.47783 | 0.47993 | 0.87147 | 0.87340 |
|  |  | Self-medication | 0.04 | 0.79533 | 0.79557 | 0.96758 | 0.96843 |
|  |  | mFTQ | 0.08 | 0.51400 | 0.51424 | 0.88888 | 0.89054 |
|  |  | ICD-10 | 0.06 | 0.54791 | 0.54832 | 0.89247 | 0.89469 |
|  |  | Withdrawal | -0.15 | 0.63994 | 0.63979 | 0.92368 | 0.92547 |
|  | rs3025411 | No. cigarettes | 0.34 | 0.17856 | 0.18130 | 0.81687 | 0.82073 |
|  |  | ND/craving | 0.84 | 0.24095 | 0.24436 | 0.81692 | 0.82073 |
|  |  | Self-medication | 0.43 | 0.02586 | 0.02682 | 0.64414 | 0.66232 |
|  |  | mFTQ | 0.14 | 0.34420 | 0.34623 | 0.83458 | 0.83774 |
|  |  | ICD-10 | 0.18 | 0.17302 | 0.17708 | 0.81132 | 0.81790 |
|  |  | Withdrawal | 0.72 | 0.05878 | 0.06090 | 0.68275 | 0.69122 |
|  | rs5320 | No. cigarettes | -0.14 | 0.65016 | 0.65268 | 0.92516 | 0.92773 |
|  |  | ND/craving | -0.38 | 0.66302 | 0.66437 | 0.93074 | 0.93263 |
|  |  | Self-medication | -0.11 | 0.64852 | 0.65018 | 0.92516 | 0.92759 |
|  |  | mFTQ | -0.20 | 0.27379 | 0.27323 | 0.81692 | 0.82073 |
|  |  | ICD-10 | -0.08 | 0.61926 | 0.62099 | 0.91735 | 0.91938 |
|  |  | Withdrawal | -0.51 | 0.28558 | 0.28639 | 0.81833 | 0.82138 |
|  | rs6271 | No. cigarettes | -0.09 | 0.73428 | 0.73270 | 0.95166 | 0.95174 |
|  |  | ND/craving | 0.16 | 0.83674 | 0.83681 | 0.97441 | 0.97550 |
|  |  | Self-medication | -0.04 | 0.85722 | 0.85602 | 0.97659 | 0.97697 |
|  |  | mFTQ | 0.02 | 0.92092 | 0.92135 | 0.98436 | 0.98444 |
|  |  | ICD-10 | -0.02 | 0.86577 | 0.86721 | 0.97659 | 0.97697 |
|  |  | Withdrawal | 0.61 | 0.14444 | 0.14672 | 0.79921 | 0.80409 |
|  | rs6479643 | No. cigarettes | -0.21 | 0.17106 | 0.17361 | 0.81132 | 0.81734 |
|  |  | ND/craving | -0.85 | 0.04868 | 0.04941 | 0.68275 | 0.69122 |
|  |  | Self-medication | -0.18 | 0.13269 | 0.13338 | 0.78635 | 0.79286 |
|  |  | mFTQ | -0.15 | 0.08550 | 0.08809 | 0.71335 | 0.72276 |
|  |  | ICD-10 | -0.16 | 0.04164 | 0.04214 | 0.68275 | 0.69122 |
|  |  | Withdrawal | -0.45 | 0.05416 | 0.05524 | 0.68275 | 0.69122 |
|  | rs732833 | No. cigarettes | 0.20 | 0.21051 | 0.21305 | 0.81692 | 0.82073 |
|  |  | ND/craving | 0.24 | 0.58740 | 0.59039 | 0.90572 | 0.90738 |
|  |  | Self-medication | 0.08 | 0.52068 | 0.52526 | 0.88888 | 0.89054 |
|  |  | mFTQ | 0.01 | 0.91515 | 0.91593 | 0.98436 | 0.98444 |
|  |  | ICD-10 | 0.03 | 0.73982 | 0.74386 | 0.95259 | 0.95552 |
|  |  | Withdrawal | -0.05 | 0.82136 | 0.82250 | 0.96985 | 0.97009 |
|  | rs77905 | No. cigarettes | 0.10 | 0.52008 | 0.51934 | 0.88888 | 0.89054 |
|  |  | ND/craving | 0.72 | 0.09810 | 0.09940 | 0.71653 | 0.72775 |
|  |  | Self-medication | 0.17 | 0.16838 | 0.16859 | 0.81132 | 0.81734 |
|  |  | mFTQ | 0.11 | 0.24754 | 0.25092 | 0.81692 | 0.82073 |
|  |  | ICD-10 | 0.09 | 0.24033 | 0.24278 | 0.81692 | 0.82073 |
|  |  | Withdrawal | 0.54 | 0.02416 | 0.02529 | 0.62050 | 0.64956 |
|  | rs7848964 | No. cigarettes | 0.18 | 0.26479 | 0.26876 | 0.81692 | 0.82073 |
|  |  | ND/craving | 0.36 | 0.43469 | 0.43893 | 0.85904 | 0.86439 |
|  |  | Self-medication | 0.11 | 0.37644 | 0.37819 | 0.84491 | 0.84876 |
|  |  | mFTQ | 0.07 | 0.45530 | 0.45863 | 0.86906 | 0.87224 |
|  |  | ICD-10 | 0.04 | 0.66492 | 0.66946 | 0.93268 | 0.93384 |
|  |  | Withdrawal | 0.10 | 0.68255 | 0.68394 | 0.93699 | 0.93823 |
|  | rs7872903 | No. cigarettes | 0.35 | 0.05744 | 0.05810 | 0.68275 | 0.69122 |
|  |  | ND/craving | 0.57 | 0.27684 | 0.27798 | 0.81692 | 0.82073 |
|  |  | Self-medication | 0.10 | 0.47240 | 0.47264 | 0.87147 | 0.87340 |
|  |  | mFTQ | 0.10 | 0.34623 | 0.34620 | 0.83458 | 0.83774 |
|  |  | ICD-10 | 0.01 | 0.94502 | 0.94498 | 0.98902 | 0.98910 |
|  |  | Withdrawal | 0.00 | 0.98893 | 0.98871 | 0.99655 | 0.99647 |
| *DDC* | rs10499696† | No. cigarettes | 0.22 | 0.45993 | 0.46206 | 0.86906 | 0.87248 |
|  |  | ND/craving | -0.01 | 0.98601 | 0.98655 | 0.99655 | 0.99647 |
|  |  | Self-medication | 0.04 | 0.86946 | 0.87289 | 0.97659 | 0.97697 |
|  |  | mFTQ | -0.06 | 0.71978 | 0.72476 | 0.94944 | 0.95098 |
|  |  | ICD-10 | -0.05 | 0.71690 | 0.71972 | 0.94933 | 0.95098 |
|  |  | Withdrawal | -0.15 | 0.73604 | 0.73919 | 0.95166 | 0.95343 |
|  | rs11238133 | No. cigarettes | -0.24 | 0.13893 | 0.13774 | 0.79750 | 0.79665 |
|  |  | ND/craving | -0.33 | 0.46683 | 0.46561 | 0.87147 | 0.87340 |
|  |  | Self-medication | -0.06 | 0.60718 | 0.60784 | 0.91291 | 0.91336 |
|  |  | mFTQ | -0.09 | 0.35761 | 0.35811 | 0.84407 | 0.84859 |
|  |  | ICD-10 | -0.01 | 0.88813 | 0.88855 | 0.97659 | 0.97697 |
|  |  | Withdrawal | -0.11 | 0.64377 | 0.64657 | 0.92403 | 0.92627 |
|  | rs11238134 | No. cigarettes | -0.26 | 0.11336 | 0.11248 | 0.74516 | 0.75032 |
|  |  | ND/craving | -0.39 | 0.39221 | 0.39186 | 0.84836 | 0.85202 |
|  |  | Self-medication | -0.07 | 0.56979 | 0.57029 | 0.89822 | 0.90234 |
|  |  | mFTQ | -0.10 | 0.29348 | 0.29401 | 0.81833 | 0.82138 |
|  |  | ICD-10 | -0.02 | 0.78763 | 0.78770 | 0.96746 | 0.96783 |
|  |  | Withdrawal | -0.15 | 0.55762 | 0.55905 | 0.89350 | 0.89469 |
|  | rs11575282 | No. cigarettes | -0.20 | 0.46582 | 0.46750 | 0.87147 | 0.87340 |
|  |  | ND/craving | -0.59 | 0.44073 | 0.44196 | 0.86332 | 0.86665 |
|  |  | Self-medication | -0.23 | 0.26618 | 0.26707 | 0.81692 | 0.82073 |
|  |  | mFTQ | -0.16 | 0.31842 | 0.32028 | 0.82728 | 0.83152 |
|  |  | ICD-10 | -0.17 | 0.22788 | 0.22946 | 0.81692 | 0.82073 |
|  |  | Withdrawal | -0.46 | 0.26994 | 0.26966 | 0.81692 | 0.82073 |
|  | rs11575383 | No. cigarettes | -0.11 | 0.69846 | 0.70140 | 0.94166 | 0.94469 |
|  |  | ND/craving | -0.17 | 0.82337 | 0.82452 | 0.96985 | 0.97009 |
|  |  | Self-medication | -0.12 | 0.56998 | 0.57411 | 0.89822 | 0.90267 |
|  |  | mFTQ | 0.02 | 0.89449 | 0.89621 | 0.97915 | 0.98018 |
|  |  | ICD-10 | -0.02 | 0.88945 | 0.88880 | 0.97659 | 0.97697 |
|  |  | Withdrawal | -0.24 | 0.56097 | 0.56313 | 0.89365 | 0.89710 |
|  | rs11575464 | No. cigarettes | 0.31 | 0.28354 | 0.28428 | 0.81833 | 0.82138 |
|  |  | ND/craving | 0.68 | 0.40362 | 0.40614 | 0.85202 | 0.85488 |
|  |  | Self-medication | -0.12 | 0.58795 | 0.59252 | 0.90572 | 0.90766 |
|  |  | mFTQ | 0.10 | 0.54744 | 0.54829 | 0.89247 | 0.89469 |
|  |  | ICD-10 | 0.03 | 0.85929 | 0.85908 | 0.97659 | 0.97697 |
|  |  | Withdrawal | 0.23 | 0.59750 | 0.59607 | 0.91084 | 0.90969 |
|  | rs11575500 | No. cigarettes | 0.11 | 0.72241 | 0.72263 | 0.94944 | 0.95098 |
|  |  | ND/craving | 0.95 | 0.26585 | 0.26592 | 0.81692 | 0.82073 |
|  |  | Self-medication | 0.14 | 0.53030 | 0.53286 | 0.89247 | 0.89469 |
|  |  | mFTQ | 0.15 | 0.39255 | 0.39389 | 0.84836 | 0.85202 |
|  |  | ICD-10 | 0.16 | 0.28959 | 0.29132 | 0.81833 | 0.82138 |
|  |  | Withdrawal | 0.03 | 0.95291 | 0.95399 | 0.99193 | 0.99216 |
|  | rs11575548 | No. cigarettes | 0.05 | 0.86657 | 0.86399 | 0.97659 | 0.97697 |
|  |  | ND/craving | 0.25 | 0.73722 | 0.73816 | 0.95166 | 0.95343 |
|  |  | Self-medication | -0.01 | 0.94310 | 0.94380 | 0.98879 | 0.98910 |
|  |  | mFTQ | 0.07 | 0.66229 | 0.66398 | 0.93039 | 0.93263 |
|  |  | ICD-10 | 0.03 | 0.84274 | 0.84227 | 0.97554 | 0.97638 |
|  |  | Withdrawal | -0.10 | 0.80181 | 0.80166 | 0.96758 | 0.96843 |
|  | rs11575553 | No. cigarettes | -0.03 | 0.90226 | 0.90249 | 0.98061 | 0.98062 |
|  |  | ND/craving | -0.01 | 0.98923 | 0.98902 | 0.99655 | 0.99647 |
|  |  | Self-medication | -0.13 | 0.53998 | 0.53947 | 0.89247 | 0.89469 |
|  |  | mFTQ | -0.03 | 0.85811 | 0.85819 | 0.97659 | 0.97697 |
|  |  | ICD-10 | -0.02 | 0.90994 | 0.90888 | 0.98402 | 0.98287 |
|  |  | Withdrawal | -0.25 | 0.55894 | 0.55976 | 0.89357 | 0.89469 |
|  | rs11575575 | No. cigarettes | -0.18 | 0.47360 | 0.47355 | 0.87147 | 0.87340 |
|  |  | ND/craving | -0.60 | 0.38869 | 0.38953 | 0.84836 | 0.85142 |
|  |  | Self-medication | -0.11 | 0.54532 | 0.54727 | 0.89247 | 0.89469 |
|  |  | mFTQ | -0.05 | 0.70422 | 0.70549 | 0.94518 | 0.94578 |
|  |  | ICD-10 | -0.10 | 0.44343 | 0.44484 | 0.86364 | 0.86716 |
|  |  | Withdrawal | -0.31 | 0.39874 | 0.40074 | 0.85047 | 0.85368 |
|  | rs11980368 | No. cigarettes | -0.18 | 0.24267 | 0.24391 | 0.81692 | 0.82073 |
|  |  | ND/craving | -0.41 | 0.33585 | 0.33642 | 0.82975 | 0.83365 |
|  |  | Self-medication | -0.10 | 0.38194 | 0.38388 | 0.84553 | 0.85010 |
|  |  | mFTQ | -0.05 | 0.56855 | 0.56805 | 0.89822 | 0.89972 |
|  |  | ICD-10 | -0.04 | 0.64269 | 0.64304 | 0.92403 | 0.92557 |
|  |  | Withdrawal | -0.21 | 0.37641 | 0.37828 | 0.84491 | 0.84876 |
|  | rs17133877 | No. cigarettes | -0.01 | 0.98757 | 0.98827 | 0.99655 | 0.99647 |
|  |  | ND/craving | -0.35 | 0.72302 | 0.72562 | 0.94944 | 0.95136 |
|  |  | Self-medication | -0.06 | 0.82499 | 0.82785 | 0.96998 | 0.97156 |
|  |  | mFTQ | -0.01 | 0.97533 | 0.97629 | 0.99655 | 0.99647 |
|  |  | ICD-10 | -0.03 | 0.86969 | 0.87081 | 0.97659 | 0.97697 |
|  |  | Withdrawal | -0.02 | 0.97219 | 0.97216 | 0.99655 | 0.99647 |
|  | rs2044859 | No. cigarettes | -0.29 | 0.08728 | 0.08916 | 0.71335 | 0.72276 |
|  |  | ND/craving | -0.89 | 0.06336 | 0.06476 | 0.68275 | 0.69122 |
|  |  | Self-medication | -0.08 | 0.55657 | 0.55909 | 0.89350 | 0.89469 |
|  |  | mFTQ | -0.17 | 0.08081 | 0.08368 | 0.71335 | 0.72276 |
|  |  | ICD-10 | -0.11 | 0.21752 | 0.22035 | 0.81692 | 0.82073 |
|  |  | Withdrawal | -0.23 | 0.36882 | 0.36976 | 0.84491 | 0.84876 |
|  | rs2060762 | No. cigarettes | -0.36 | 0.06302 | 0.06415 | 0.68275 | 0.69122 |
|  |  | ND/craving | -0.76 | 0.16476 | 0.16761 | 0.81132 | 0.81734 |
|  |  | Self-medication | -0.18 | 0.22270 | 0.22747 | 0.81692 | 0.82073 |
|  |  | mFTQ | -0.19 | 0.08786 | 0.08950 | 0.71335 | 0.72276 |
|  |  | ICD-10 | -0.13 | 0.20787 | 0.20888 | 0.81692 | 0.82073 |
|  |  | Withdrawal | -0.11 | 0.70069 | 0.70439 | 0.94241 | 0.94578 |
|  | rs2167364 | No. cigarettes | 0.07 | 0.67256 | 0.67454 | 0.93294 | 0.93426 |
|  |  | ND/craving | -0.06 | 0.90512 | 0.90572 | 0.98101 | 0.98166 |
|  |  | Self-medication | -0.01 | 0.91799 | 0.91974 | 0.98436 | 0.98444 |
|  |  | mFTQ | 0.01 | 0.90577 | 0.90635 | 0.98117 | 0.98172 |
|  |  | ICD-10 | -0.03 | 0.70496 | 0.70513 | 0.94551 | 0.94578 |
|  |  | Withdrawal | -0.24 | 0.36400 | 0.36697 | 0.84491 | 0.84876 |
|  | rs3735273 | No. cigarettes | -0.35 | 0.05020 | 0.05159 | 0.68275 | 0.69122 |
|  |  | ND/craving | -0.75 | 0.12977 | 0.13136 | 0.77907 | 0.78587 |
|  |  | Self-medication | -0.10 | 0.44661 | 0.45075 | 0.86722 | 0.87224 |
|  |  | mFTQ | -0.17 | 0.10457 | 0.10807 | 0.73503 | 0.75032 |
|  |  | ICD-10 | -0.06 | 0.48536 | 0.48468 | 0.87147 | 0.87340 |
|  |  | Withdrawal | -0.12 | 0.65974 | 0.65927 | 0.92885 | 0.92887 |
|  | rs3779078 | No. cigarettes | -0.40 | 0.02807 | 0.02889 | 0.66747 | 0.69083 |
|  |  | ND/craving | -0.86 | 0.09297 | 0.09478 | 0.71653 | 0.72775 |
|  |  | Self-medication | -0.14 | 0.33188 | 0.33655 | 0.82975 | 0.83365 |
|  |  | mFTQ | -0.19 | 0.07117 | 0.07345 | 0.68903 | 0.70731 |
|  |  | ICD-10 | -0.09 | 0.32581 | 0.32659 | 0.82813 | 0.83152 |
|  |  | Withdrawal | -0.26 | 0.35749 | 0.36017 | 0.84407 | 0.84859 |
|  | rs4947510 | No. cigarettes | -0.28 | 0.10307 | 0.10420 | 0.73197 | 0.73783 |
|  |  | ND/craving | -0.54 | 0.25774 | 0.25670 | 0.81692 | 0.82073 |
|  |  | Self-medication | -0.16 | 0.22092 | 0.22241 | 0.81692 | 0.82073 |
|  |  | mFTQ | -0.14 | 0.17059 | 0.17207 | 0.81132 | 0.81734 |
|  |  | ICD-10 | -0.08 | 0.37718 | 0.37614 | 0.84491 | 0.84876 |
|  |  | Withdrawal | -0.10 | 0.69778 | 0.69932 | 0.94166 | 0.94333 |
|  | rs4947644 | No. cigarettes | -0.38 | 0.01603 | 0.01672 | 0.57781 | 0.58246 |
|  |  | ND/craving | -1.14 | 0.00977 | 0.01026 | 0.52414 | 0.53402 |
|  |  | Self-medication | -0.21 | 0.08519 | 0.08777 | 0.71335 | 0.72276 |
|  |  | mFTQ | -0.24 | 0.01048 | 0.01107 | 0.52414 | 0.53402 |
|  |  | ICD-10 | -0.15 | 0.06603 | 0.06731 | 0.68275 | 0.69122 |
|  |  | Withdrawal | -0.48 | 0.04707 | 0.04812 | 0.68275 | 0.69122 |
|  | rs6592963 | No. cigarettes | -0.10 | 0.60532 | 0.60650 | 0.91171 | 0.91331 |
|  |  | ND/craving | -0.64 | 0.24063 | 0.24307 | 0.81692 | 0.82073 |
|  |  | Self-medication | -0.16 | 0.26563 | 0.26706 | 0.81692 | 0.82073 |
|  |  | mFTQ | -0.14 | 0.20396 | 0.20565 | 0.81687 | 0.82073 |
|  |  | ICD-10 | -0.17 | 0.08518 | 0.08679 | 0.71335 | 0.72276 |
|  |  | Withdrawal | -0.47 | 0.11046 | 0.11090 | 0.74516 | 0.75032 |
|  | rs6593011 | No. cigarettes | 0.23 | 0.34682 | 0.35055 | 0.83496 | 0.84081 |
|  |  | ND/craving | -0.11 | 0.86864 | 0.87095 | 0.97659 | 0.97697 |
|  |  | Self-medication | 0.02 | 0.91661 | 0.91863 | 0.98436 | 0.98444 |
|  |  | mFTQ | -0.02 | 0.85931 | 0.86111 | 0.97659 | 0.97697 |
|  |  | ICD-10 | -0.09 | 0.46729 | 0.47304 | 0.87147 | 0.87340 |
|  |  | Withdrawal | -0.20 | 0.58933 | 0.59235 | 0.90572 | 0.90766 |
|  | rs6951648 | No. cigarettes | 0.16 | 0.42458 | 0.42589 | 0.85803 | 0.86008 |
|  |  | ND/craving | -0.02 | 0.97742 | 0.97776 | 0.99655 | 0.99647 |
|  |  | Self-medication | 0.06 | 0.70651 | 0.70680 | 0.94554 | 0.94578 |
|  |  | mFTQ | 0.02 | 0.88157 | 0.88196 | 0.97659 | 0.97697 |
|  |  | ICD-10 | -0.02 | 0.84587 | 0.84609 | 0.97554 | 0.97638 |
|  |  | Withdrawal | -0.20 | 0.49408 | 0.49510 | 0.87945 | 0.88128 |
|  | rs880028 | No. cigarettes | 0.03 | 0.88669 | 0.88796 | 0.97659 | 0.97697 |
|  |  | ND/craving | -0.05 | 0.92186 | 0.92216 | 0.98451 | 0.98452 |
|  |  | Self-medication | -0.13 | 0.35987 | 0.36313 | 0.84491 | 0.84876 |
|  |  | mFTQ | 0.06 | 0.61330 | 0.61481 | 0.91370 | 0.91509 |
|  |  | ICD-10 | -0.03 | 0.75648 | 0.75751 | 0.95749 | 0.95912 |
|  |  | Withdrawal | -0.32 | 0.26773 | 0.26989 | 0.81692 | 0.82073 |
|  | rs921451 | No. cigarettes | -0.44 | 0.00568 | 0.00608 | 0.48756 | 0.49807 |
|  |  | ND/craving | -1.01 | 0.02345 | 0.02396 | 0.61858 | 0.62356 |
|  |  | Self-medication | -0.21 | 0.08446 | 0.08742 | 0.71335 | 0.72276 |
|  |  | mFTQ | -0.20 | 0.03351 | 0.03417 | 0.68275 | 0.69122 |
|  |  | ICD-10 | -0.12 | 0.15335 | 0.15547 | 0.81063 | 0.81147 |
|  |  | Withdrawal | -0.35 | 0.15256 | 0.15362 | 0.81063 | 0.81147 |
|  | rs978784 | No. cigarettes | -0.41 | 0.02297 | 0.02389 | 0.61858 | 0.62356 |
|  |  | ND/craving | -1.08 | 0.03072 | 0.03170 | 0.68275 | 0.69122 |
|  |  | Self-medication | -0.14 | 0.32577 | 0.33099 | 0.82813 | 0.83365 |
|  |  | mFTQ | -0.20 | 0.05183 | 0.05422 | 0.68275 | 0.69122 |
|  |  | ICD-10 | -0.09 | 0.31331 | 0.31453 | 0.82443 | 0.82983 |
|  |  | Withdrawal | -0.20 | 0.45720 | 0.46131 | 0.86906 | 0.87224 |
| *DRD2* | rs1079597 | No. cigarettes | 0.15 | 0.47442 | 0.47615 | 0.87147 | 0.87340 |
|  |  | ND/craving | 0.19 | 0.74778 | 0.74672 | 0.95566 | 0.95560 |
|  |  | Self-medication | 0.02 | 0.91469 | 0.91547 | 0.98436 | 0.98444 |
|  |  | mFTQ | 0.05 | 0.67260 | 0.67105 | 0.93294 | 0.93384 |
|  |  | ICD-10 | 0.08 | 0.45690 | 0.45616 | 0.86906 | 0.87224 |
|  |  | Withdrawal | 0.08 | 0.79893 | 0.79826 | 0.96758 | 0.96843 |
|  | rs1107162 | No. cigarettes | -0.40 | 0.01074 | 0.01112 | 0.52414 | 0.53402 |
|  |  | ND/craving | -0.80 | 0.06592 | 0.06662 | 0.68275 | 0.69122 |
|  |  | Self-medication | -0.24 | 0.04142 | 0.04109 | 0.68275 | 0.69122 |
|  |  | mFTQ | -0.19 | 0.03703 | 0.03745 | 0.68275 | 0.69122 |
|  |  | ICD-10 | -0.12 | 0.14420 | 0.14581 | 0.79921 | 0.80409 |
|  |  | Withdrawal | -0.50 | 0.03252 | 0.03194 | 0.68275 | 0.69122 |
|  | rs11214606† | No. cigarettes | -0.18 | 0.60704 | 0.60799 | 0.91291 | 0.91336 |
|  |  | ND/craving | -0.40 | 0.69522 | 0.69755 | 0.94166 | 0.94333 |
|  |  | Self-medication | 0.22 | 0.43055 | 0.43541 | 0.85904 | 0.86375 |
|  |  | mFTQ | -0.15 | 0.47853 | 0.48350 | 0.87147 | 0.87340 |
|  |  | ICD-10 | -0.07 | 0.71458 | 0.71715 | 0.94741 | 0.94996 |
|  |  | Withdrawal | -0.50 | 0.36518 | 0.36863 | 0.84491 | 0.84876 |
|  | rs17529477 | No. cigarettes | 0.21 | 0.19234 | 0.19338 | 0.81687 | 0.82073 |
|  |  | ND/craving | 0.52 | 0.24881 | 0.25226 | 0.81692 | 0.82073 |
|  |  | Self-medication | 0.09 | 0.47978 | 0.48190 | 0.87147 | 0.87340 |
|  |  | mFTQ | 0.08 | 0.39809 | 0.39858 | 0.85001 | 0.85202 |
|  |  | ICD-10 | 0.06 | 0.49361 | 0.49366 | 0.87945 | 0.88117 |
|  |  | Withdrawal | 0.39 | 0.10588 | 0.10868 | 0.73616 | 0.75032 |
|  | rs1799978 | No. cigarettes | -0.43 | 0.19576 | 0.19795 | 0.81687 | 0.82073 |
|  |  | ND/craving | 0.43 | 0.65178 | 0.65505 | 0.92536 | 0.92797 |
|  |  | Self-medication | 0.09 | 0.73246 | 0.73328 | 0.95166 | 0.95174 |
|  |  | mFTQ | 0.09 | 0.63886 | 0.64207 | 0.92368 | 0.92557 |
|  |  | ICD-10 | -0.03 | 0.87631 | 0.87771 | 0.97659 | 0.97697 |
|  |  | Withdrawal | 0.37 | 0.47753 | 0.48272 | 0.87147 | 0.87340 |
|  | rs2440390 | No. cigarettes | -0.46 | 0.03781 | 0.03829 | 0.68275 | 0.69122 |
|  |  | ND/craving | -1.01 | 0.10583 | 0.10682 | 0.73616 | 0.74810 |
|  |  | Self-medication | -0.21 | 0.21670 | 0.21860 | 0.81692 | 0.82073 |
|  |  | mFTQ | -0.22 | 0.09261 | 0.09382 | 0.71653 | 0.72775 |
|  |  | ICD-10 | -0.14 | 0.23644 | 0.23911 | 0.81692 | 0.82073 |
|  |  | Withdrawal | -0.62 | 0.06590 | 0.06770 | 0.68275 | 0.69122 |
|  | rs4245146 | No. cigarettes | 0.02 | 0.91559 | 0.91497 | 0.98436 | 0.98444 |
|  |  | ND/craving | 0.13 | 0.77238 | 0.77492 | 0.96099 | 0.96227 |
|  |  | Self-medication | 0.00 | 0.99394 | 0.99409 | 0.99801 | 0.99789 |
|  |  | mFTQ | -0.03 | 0.76188 | 0.76559 | 0.95755 | 0.95944 |
|  |  | ICD-10 | 0.00 | 0.98406 | 0.98397 | 0.99655 | 0.99647 |
|  |  | Withdrawal | 0.07 | 0.76062 | 0.76414 | 0.95749 | 0.95944 |
|  | rs4586205 | No. cigarettes | -0.55 | 0.00114 | 0.00129 | 0.15836 | 0.17757 |
|  |  | ND/craving | -0.94 | 0.04322 | 0.04393 | 0.68275 | 0.69122 |
|  |  | Self-medication | -0.26 | 0.04585 | 0.04682 | 0.68275 | 0.69122 |
|  |  | mFTQ | -0.23 | 0.01696 | 0.01720 | 0.57781 | 0.58246 |
|  |  | ICD-10 | -0.15 | 0.07805 | 0.07919 | 0.71245 | 0.72276 |
|  |  | Withdrawal | -0.43 | 0.09319 | 0.09359 | 0.71653 | 0.72775 |
|  | rs4630328 | No. cigarettes | 0.16 | 0.29934 | 0.30298 | 0.82443 | 0.82839 |
|  |  | ND/craving | 0.37 | 0.39480 | 0.39745 | 0.84836 | 0.85202 |
|  |  | Self-medication | 0.05 | 0.68835 | 0.68995 | 0.93909 | 0.94037 |
|  |  | mFTQ | 0.05 | 0.59071 | 0.59078 | 0.90572 | 0.90738 |
|  |  | ICD-10 | 0.03 | 0.69713 | 0.69916 | 0.94166 | 0.94333 |
|  |  | Withdrawal | 0.19 | 0.41557 | 0.41801 | 0.85340 | 0.85620 |
|  | rs4936270 | No. cigarettes | -0.87 | 0.00115 | 0.00113 | 0.15836 | 0.16775 |
|  |  | ND/craving | -1.39 | 0.06503 | 0.06635 | 0.68275 | 0.69122 |
|  |  | Self-medication | -0.29 | 0.15806 | 0.16068 | 0.81063 | 0.81586 |
|  |  | mFTQ | -0.40 | 0.01112 | 0.01145 | 0.52414 | 0.53402 |
|  |  | ICD-10 | -0.25 | 0.06962 | 0.07092 | 0.68903 | 0.69122 |
|  |  | Withdrawal | -0.71 | 0.08660 | 0.08782 | 0.71335 | 0.72276 |
|  | rs4938019 | No. cigarettes | 0.02 | 0.93998 | 0.93963 | 0.98821 | 0.98861 |
|  |  | ND/craving | -0.14 | 0.82076 | 0.82262 | 0.96985 | 0.97009 |
|  |  | Self-medication | -0.05 | 0.76377 | 0.76666 | 0.95770 | 0.95944 |
|  |  | mFTQ | 0.07 | 0.57928 | 0.58087 | 0.90047 | 0.90294 |
|  |  | ICD-10 | 0.01 | 0.92366 | 0.92610 | 0.98504 | 0.98615 |
|  |  | Withdrawal | -0.07 | 0.83142 | 0.83154 | 0.97193 | 0.97182 |
|  | rs6276 | No. cigarettes | -0.44 | 0.00700 | 0.00722 | 0.49934 | 0.51529 |
|  |  | ND/craving | -0.72 | 0.11292 | 0.11432 | 0.74516 | 0.75032 |
|  |  | Self-medication | -0.23 | 0.06382 | 0.06488 | 0.68275 | 0.69122 |
|  |  | mFTQ | -0.19 | 0.04735 | 0.04795 | 0.68275 | 0.69122 |
|  |  | ICD-10 | -0.12 | 0.14066 | 0.14257 | 0.79921 | 0.80409 |
|  |  | Withdrawal | -0.52 | 0.03549 | 0.03580 | 0.68275 | 0.69122 |
|  | rs7125415 | No. cigarettes | -0.45 | 0.06564 | 0.06644 | 0.68275 | 0.69122 |
|  |  | ND/craving | -0.78 | 0.24991 | 0.25153 | 0.81692 | 0.82073 |
|  |  | Self-medication | -0.25 | 0.17403 | 0.17555 | 0.81132 | 0.81734 |
|  |  | mFTQ | -0.21 | 0.13440 | 0.13469 | 0.79100 | 0.79456 |
|  |  | ICD-10 | -0.19 | 0.11568 | 0.11748 | 0.74516 | 0.75032 |
|  |  | Withdrawal | -0.38 | 0.30299 | 0.30197 | 0.82443 | 0.82839 |
|  | rs7131056 | No. cigarettes | -0.13 | 0.41674 | 0.41832 | 0.85388 | 0.85620 |
|  |  | ND/craving | -0.38 | 0.38667 | 0.38922 | 0.84836 | 0.85142 |
|  |  | Self-medication | -0.01 | 0.92075 | 0.92055 | 0.98436 | 0.98444 |
|  |  | mFTQ | -0.11 | 0.21844 | 0.22108 | 0.81692 | 0.82073 |
|  |  | ICD-10 | -0.02 | 0.80108 | 0.80077 | 0.96758 | 0.96843 |
|  |  | Withdrawal | -0.20 | 0.40772 | 0.41302 | 0.85202 | 0.85586 |
| *DRD4* | rs752306† | No. cigarettes | -0.35 | 0.25829 | 0.25816 | 0.81692 | 0.82073 |
|  |  | ND/craving | -0.93 | 0.27749 | 0.27823 | 0.81692 | 0.82073 |
|  |  | Self-medication | -0.39 | 0.09783 | 0.09803 | 0.71653 | 0.72775 |
|  |  | mFTQ | -0.22 | 0.21973 | 0.21832 | 0.81692 | 0.82073 |
|  |  | ICD-10 | -0.29 | 0.06205 | 0.06221 | 0.68275 | 0.69122 |
|  |  | Withdrawal | -0.62 | 0.18861 | 0.18820 | 0.81687 | 0.82073 |
|  | rs4331145 | No. cigarettes | -0.06 | 0.68675 | 0.68911 | 0.93866 | 0.94037 |
|  |  | ND/craving | -0.32 | 0.46971 | 0.47107 | 0.87147 | 0.87340 |
|  |  | Self-medication | -0.09 | 0.47663 | 0.47717 | 0.87147 | 0.87340 |
|  |  | mFTQ | 0.00 | 0.98096 | 0.98125 | 0.99655 | 0.99647 |
|  |  | ICD-10 | -0.05 | 0.51281 | 0.51407 | 0.88888 | 0.89054 |
|  |  | Withdrawal | -0.26 | 0.27677 | 0.27703 | 0.81692 | 0.82073 |
|  | rs3758653 | No. cigarettes | 0.32 | 0.12054 | 0.12253 | 0.75377 | 0.76622 |
|  |  | ND/craving | 0.56 | 0.33238 | 0.33467 | 0.82975 | 0.83365 |
|  |  | Self-medication | 0.02 | 0.89165 | 0.89333 | 0.97785 | 0.97873 |
|  |  | mFTQ | 0.08 | 0.51836 | 0.52015 | 0.88888 | 0.89054 |
|  |  | ICD-10 | 0.01 | 0.92735 | 0.92800 | 0.98569 | 0.98638 |
|  |  | Withdrawal | -0.05 | 0.86086 | 0.86231 | 0.97659 | 0.97697 |
|  | rs11246228 | No. cigarettes | 0.02 | 0.92035 | 0.91961 | 0.98436 | 0.98444 |
|  |  | ND/craving | 0.19 | 0.67718 | 0.67772 | 0.93294 | 0.93426 |
|  |  | Self-medication | 0.10 | 0.40373 | 0.40641 | 0.85202 | 0.85488 |
|  |  | mFTQ | -0.03 | 0.73561 | 0.73546 | 0.95166 | 0.95323 |
|  |  | ICD-10 | 0.04 | 0.58893 | 0.59004 | 0.90572 | 0.90738 |
|  |  | Withdrawal | 0.36 | 0.14909 | 0.14973 | 0.80364 | 0.80972 |
| *EGLN2* | rs7937 | No. cigarettes | 0.01 | 0.95355 | 0.95404 | 0.99193 | 0.99216 |
|  |  | ND/craving | 0.20 | 0.65088 | 0.65255 | 0.92516 | 0.92773 |
|  |  | Self-medication | 0.09 | 0.42646 | 0.42993 | 0.85803 | 0.86164 |
|  |  | mFTQ | 0.08 | 0.40236 | 0.40262 | 0.85202 | 0.85488 |
|  |  | ICD-10 | 0.08 | 0.28633 | 0.28736 | 0.81833 | 0.82138 |
|  |  | Withdrawal | 0.45 | 0.05618 | 0.05656 | 0.68275 | 0.69122 |
|  | rs4803372 | No. cigarettes | -0.07 | 0.65761 | 0.65830 | 0.92788 | 0.92887 |
|  |  | ND/craving | 0.31 | 0.48009 | 0.48041 | 0.87147 | 0.87340 |
|  |  | Self-medication | 0.02 | 0.84816 | 0.84823 | 0.97659 | 0.97697 |
|  |  | mFTQ | 0.03 | 0.74758 | 0.74616 | 0.95566 | 0.95552 |
|  |  | ICD-10 | 0.07 | 0.34746 | 0.34983 | 0.83547 | 0.84012 |
|  |  | Withdrawal | 0.17 | 0.46372 | 0.46487 | 0.87147 | 0.87340 |
|  | rs3733829 | No. cigarettes | 0.03 | 0.84557 | 0.84606 | 0.97554 | 0.97638 |
|  |  | ND/craving | -0.37 | 0.40556 | 0.40549 | 0.85202 | 0.85488 |
|  |  | Self-medication | -0.07 | 0.56673 | 0.56755 | 0.89822 | 0.89972 |
|  |  | mFTQ | -0.09 | 0.30292 | 0.30396 | 0.82443 | 0.82839 |
|  |  | ICD-10 | -0.11 | 0.16661 | 0.16795 | 0.81132 | 0.81734 |
|  |  | Withdrawal | -0.48 | 0.04381 | 0.04415 | 0.68275 | 0.69122 |
|  | rs2644916 | No. cigarettes | -0.03 | 0.88144 | 0.88254 | 0.97659 | 0.97697 |
|  |  | ND/craving | 0.45 | 0.37476 | 0.37746 | 0.84491 | 0.84876 |
|  |  | Self-medication | 0.11 | 0.42683 | 0.43102 | 0.85803 | 0.86205 |
|  |  | mFTQ | 0.07 | 0.52203 | 0.52276 | 0.88888 | 0.89054 |
|  |  | ICD-10 | 0.11 | 0.21629 | 0.21783 | 0.81692 | 0.82073 |
|  |  | Withdrawal | 0.38 | 0.16845 | 0.16930 | 0.81132 | 0.81734 |
|  | rs11881124 | No. cigarettes | -0.19 | 0.31086 | 0.31234 | 0.82443 | 0.82983 |
|  |  | ND/craving | -0.60 | 0.25783 | 0.25965 | 0.81692 | 0.82073 |
|  |  | Self-medication | -0.24 | 0.09596 | 0.09775 | 0.71653 | 0.72775 |
|  |  | mFTQ | -0.13 | 0.23611 | 0.23847 | 0.81692 | 0.82073 |
|  |  | ICD-10 | -0.16 | 0.09921 | 0.10026 | 0.71837 | 0.72775 |
|  |  | Withdrawal | -0.25 | 0.38392 | 0.38520 | 0.84797 | 0.85020 |
|  | rs11666504 | No. cigarettes | -0.02 | 0.91551 | 0.91449 | 0.98436 | 0.98444 |
|  |  | ND/craving | 0.28 | 0.62858 | 0.62879 | 0.92274 | 0.92348 |
|  |  | Self-medication | -0.02 | 0.87978 | 0.88022 | 0.97659 | 0.97697 |
|  |  | mFTQ | 0.02 | 0.86887 | 0.86736 | 0.97659 | 0.97697 |
|  |  | ICD-10 | 0.05 | 0.63606 | 0.63602 | 0.92368 | 0.92547 |
|  |  | Withdrawal | 0.03 | 0.92076 | 0.92075 | 0.98436 | 0.98444 |
|  | rs11083568 | No. cigarettes | -0.28 | 0.16075 | 0.16224 | 0.81132 | 0.81586 |
|  |  | ND/craving | -0.93 | 0.09720 | 0.09858 | 0.71653 | 0.72775 |
|  |  | Self-medication | -0.20 | 0.18205 | 0.18227 | 0.81687 | 0.82073 |
|  |  | mFTQ | -0.11 | 0.35517 | 0.35791 | 0.84407 | 0.84859 |
|  |  | ICD-10 | -0.15 | 0.14189 | 0.14367 | 0.79921 | 0.80409 |
|  |  | Withdrawal | -0.03 | 0.91322 | 0.91411 | 0.98436 | 0.98444 |
|  | rs10405596 | No. cigarettes | -0.20 | 0.36980 | 0.37336 | 0.84491 | 0.84876 |
|  |  | ND/craving | -0.70 | 0.25839 | 0.26105 | 0.81692 | 0.82073 |
|  |  | Self-medication | -0.21 | 0.21079 | 0.21102 | 0.81692 | 0.82073 |
|  |  | mFTQ | -0.01 | 0.93164 | 0.93108 | 0.98732 | 0.98766 |
|  |  | ICD-10 | -0.12 | 0.26828 | 0.27070 | 0.81692 | 0.82073 |
|  |  | Withdrawal | 0.13 | 0.70711 | 0.70948 | 0.94554 | 0.94578 |
|  | rs10403040 | No. cigarettes | -0.33 | 0.11821 | 0.11933 | 0.74683 | 0.75352 |
|  |  | ND/craving | -0.99 | 0.09764 | 0.09909 | 0.71653 | 0.72775 |
|  |  | Self-medication | -0.30 | 0.06915 | 0.07062 | 0.68903 | 0.69122 |
|  |  | mFTQ | -0.10 | 0.43426 | 0.43770 | 0.85904 | 0.86375 |
|  |  | ICD-10 | -0.20 | 0.06771 | 0.06957 | 0.68275 | 0.69122 |
|  |  | Withdrawal | -0.18 | 0.57714 | 0.58065 | 0.89933 | 0.90294 |
| *GSTM1* | rs534314† | No. cigarettes | -0.34 | 0.20720 | 0.21044 | 0.81692 | 0.82073 |
|  |  | ND/craving | -1.21 | 0.11580 | 0.11746 | 0.74516 | 0.75032 |
|  |  | Self-medication | -0.29 | 0.15991 | 0.16080 | 0.81074 | 0.81586 |
|  |  | mFTQ | -0.28 | 0.08155 | 0.08226 | 0.71335 | 0.72276 |
|  |  | ICD-10 | -0.24 | 0.08044 | 0.08114 | 0.71335 | 0.72276 |
|  |  | Withdrawal | -0.43 | 0.30368 | 0.30290 | 0.82443 | 0.82839 |
| *MAO-A* | rs1137070 | No. cigarettes | 0.00 | 0.98383 | 0.98382 | 0.99655 | 0.99647 |
|  |  | ND/craving | 0.09 | 0.81596 | 0.81589 | 0.96885 | 0.97009 |
|  |  | Self-medication | 0.04 | 0.73991 | 0.74007 | 0.95259 | 0.95343 |
|  |  | mFTQ | -0.04 | 0.59777 | 0.59992 | 0.91084 | 0.91247 |
|  |  | ICD-10 | 0.04 | 0.60304 | 0.60462 | 0.91171 | 0.91247 |
|  |  | Withdrawal | 0.21 | 0.33468 | 0.33804 | 0.82975 | 0.83365 |
|  | rs12843533 | No. cigarettes | 0.31 | 0.27240 | 0.27459 | 0.81692 | 0.82073 |
|  |  | ND/craving | -0.13 | 0.86929 | 0.87125 | 0.97659 | 0.97697 |
|  |  | Self-medication | -0.12 | 0.58107 | 0.58285 | 0.90108 | 0.90467 |
|  |  | mFTQ | 0.06 | 0.72283 | 0.72464 | 0.94944 | 0.95098 |
|  |  | ICD-10 | -0.02 | 0.88548 | 0.88812 | 0.97659 | 0.97697 |
|  |  | Withdrawal | -0.40 | 0.33917 | 0.33929 | 0.83024 | 0.83365 |
|  | rs3027415 | No. cigarettes | 0.28 | 0.06293 | 0.06369 | 0.68275 | 0.69122 |
|  |  | ND/craving | 0.71 | 0.09226 | 0.09256 | 0.71653 | 0.72764 |
|  |  | Self-medication | 0.23 | 0.04931 | 0.04993 | 0.68275 | 0.69122 |
|  |  | mFTQ | 0.13 | 0.15387 | 0.15436 | 0.81063 | 0.81147 |
|  |  | ICD-10 | 0.09 | 0.24056 | 0.24315 | 0.81692 | 0.82073 |
|  |  | Withdrawal | 0.25 | 0.28156 | 0.28317 | 0.81833 | 0.82138 |
|  | rs5906957 | No. cigarettes | -0.05 | 0.75388 | 0.75365 | 0.95749 | 0.95849 |
|  |  | ND/craving | -0.03 | 0.94702 | 0.94723 | 0.98967 | 0.98988 |
|  |  | Self-medication | -0.02 | 0.84327 | 0.84376 | 0.97554 | 0.97638 |
|  |  | mFTQ | -0.02 | 0.78585 | 0.78696 | 0.96713 | 0.96783 |
|  |  | ICD-10 | 0.02 | 0.82645 | 0.82628 | 0.96998 | 0.97097 |
|  |  | Withdrawal | 0.13 | 0.58197 | 0.58448 | 0.90175 | 0.90491 |
|  | rs6609257 | No. cigarettes | -0.05 | 0.69510 | 0.69726 | 0.94166 | 0.94333 |
|  |  | ND/craving | -0.05 | 0.88777 | 0.88727 | 0.97659 | 0.97697 |
|  |  | Self-medication | 0.02 | 0.84397 | 0.84316 | 0.97554 | 0.97638 |
|  |  | mFTQ | -0.08 | 0.30549 | 0.30839 | 0.82443 | 0.82839 |
|  |  | ICD-10 | 0.03 | 0.64535 | 0.64852 | 0.92403 | 0.92659 |
|  |  | Withdrawal | 0.11 | 0.58024 | 0.58375 | 0.90052 | 0.90491 |
|  | rs6610845 | No. cigarettes | -0.04 | 0.76584 | 0.76523 | 0.95851 | 0.95944 |
|  |  | ND/craving | 0.08 | 0.82807 | 0.82944 | 0.97129 | 0.97156 |
|  |  | Self-medication | 0.02 | 0.83288 | 0.83375 | 0.97220 | 0.97322 |
|  |  | mFTQ | -0.06 | 0.41562 | 0.41688 | 0.85340 | 0.85620 |
|  |  | ICD-10 | 0.04 | 0.52365 | 0.52399 | 0.88948 | 0.89054 |
|  |  | Withdrawal | 0.19 | 0.34606 | 0.34949 | 0.83458 | 0.84012 |
| *NR4A2* | rs7564993 | No. cigarettes | -0.24 | 0.43621 | 0.43646 | 0.85904 | 0.86375 |
|  |  | ND/craving | -1.02 | 0.23527 | 0.23833 | 0.81692 | 0.82073 |
|  |  | Self-medication | -0.47 | 0.04536 | 0.04567 | 0.68275 | 0.69122 |
|  |  | mFTQ | -0.23 | 0.19453 | 0.19567 | 0.81687 | 0.82073 |
|  |  | ICD-10 | -0.26 | 0.09104 | 0.09016 | 0.71653 | 0.72276 |
|  |  | Withdrawal | -0.96 | 0.03763 | 0.03734 | 0.68275 | 0.69122 |
|  | rs834835 | No. cigarettes | -0.12 | 0.47538 | 0.47767 | 0.87147 | 0.87340 |
|  |  | ND/craving | -0.53 | 0.26683 | 0.26808 | 0.81692 | 0.82073 |
|  |  | Self-medication | -0.21 | 0.11324 | 0.11449 | 0.74516 | 0.75032 |
|  |  | mFTQ | -0.14 | 0.15651 | 0.15731 | 0.81063 | 0.81586 |
|  |  | ICD-10 | -0.07 | 0.41722 | 0.41769 | 0.85396 | 0.85620 |
|  |  | Withdrawal | -0.27 | 0.29195 | 0.29133 | 0.81833 | 0.82138 |
| *OPRM1* | rs10223804 | No. cigarettes | -0.30 | 0.19721 | 0.19946 | 0.81687 | 0.82073 |
|  |  | ND/craving | -0.72 | 0.26278 | 0.26452 | 0.81692 | 0.82073 |
|  |  | Self-medication | -0.26 | 0.14206 | 0.14310 | 0.79921 | 0.80409 |
|  |  | mFTQ | -0.10 | 0.46811 | 0.46806 | 0.87147 | 0.87340 |
|  |  | ICD-10 | -0.07 | 0.57563 | 0.57794 | 0.89916 | 0.90267 |
|  |  | Withdrawal | -0.23 | 0.50942 | 0.51178 | 0.88826 | 0.89041 |
|  | rs10485058 | No. cigarettes | -0.25 | 0.29098 | 0.29403 | 0.81833 | 0.82138 |
|  |  | ND/craving | -0.73 | 0.26559 | 0.26687 | 0.81692 | 0.82073 |
|  |  | Self-medication | -0.23 | 0.19472 | 0.19666 | 0.81687 | 0.82073 |
|  |  | mFTQ | -0.11 | 0.42611 | 0.42748 | 0.85803 | 0.86008 |
|  |  | ICD-10 | -0.10 | 0.38821 | 0.38972 | 0.84836 | 0.85142 |
|  |  | Withdrawal | -0.21 | 0.54579 | 0.54788 | 0.89247 | 0.89469 |
|  | rs10485060† | No. cigarettes | -0.03 | 0.93441 | 0.93356 | 0.98732 | 0.98766 |
|  |  | ND/craving | -0.14 | 0.88784 | 0.88969 | 0.97659 | 0.97697 |
|  |  | Self-medication | -0.05 | 0.86638 | 0.86661 | 0.97659 | 0.97697 |
|  |  | mFTQ | 0.03 | 0.90322 | 0.90374 | 0.98061 | 0.98062 |
|  |  | ICD-10 | -0.09 | 0.61459 | 0.61727 | 0.91405 | 0.91733 |
|  |  | Withdrawal | -0.34 | 0.53725 | 0.54009 | 0.89247 | 0.89469 |
|  | rs11155954 | No. cigarettes | -0.05 | 0.81733 | 0.81771 | 0.96885 | 0.97009 |
|  |  | ND/craving | 0.41 | 0.47442 | 0.47705 | 0.87147 | 0.87340 |
|  |  | Self-medication | 0.04 | 0.80237 | 0.80329 | 0.96758 | 0.96843 |
|  |  | mFTQ | 0.11 | 0.36744 | 0.36976 | 0.84491 | 0.84876 |
|  |  | ICD-10 | 0.05 | 0.61829 | 0.61913 | 0.91735 | 0.91869 |
|  |  | Withdrawal | -0.19 | 0.53562 | 0.53839 | 0.89247 | 0.89469 |
|  | rs11965988 | No. cigarettes | -0.11 | 0.72775 | 0.72885 | 0.95166 | 0.95174 |
|  |  | ND/craving | -1.04 | 0.23037 | 0.23444 | 0.81692 | 0.82073 |
|  |  | Self-medication | -0.18 | 0.43936 | 0.44232 | 0.86332 | 0.86665 |
|  |  | mFTQ | -0.05 | 0.78154 | 0.78010 | 0.96429 | 0.96319 |
|  |  | ICD-10 | -0.13 | 0.39300 | 0.39728 | 0.84836 | 0.85202 |
|  |  | Withdrawal | 0.00 | 0.99989 | 0.99990 | 0.99989 | 0.99990 |
|  | rs12199124 | No. cigarettes | -0.35 | 0.20182 | 0.20327 | 0.81687 | 0.82073 |
|  |  | ND/craving | -0.70 | 0.36527 | 0.36809 | 0.84491 | 0.84876 |
|  |  | Self-medication | -0.13 | 0.54112 | 0.54350 | 0.89247 | 0.89469 |
|  |  | mFTQ | -0.18 | 0.25560 | 0.25734 | 0.81692 | 0.82073 |
|  |  | ICD-10 | -0.14 | 0.32163 | 0.32319 | 0.82813 | 0.83152 |
|  |  | Withdrawal | -0.18 | 0.67498 | 0.67784 | 0.93294 | 0.93426 |
|  | rs12527056 | No. cigarettes | -0.22 | 0.32367 | 0.32414 | 0.82813 | 0.83152 |
|  |  | ND/craving | 0.07 | 0.91385 | 0.91341 | 0.98436 | 0.98444 |
|  |  | Self-medication | 0.08 | 0.63133 | 0.63502 | 0.92368 | 0.92547 |
|  |  | mFTQ | -0.07 | 0.61070 | 0.61253 | 0.91320 | 0.91476 |
|  |  | ICD-10 | 0.01 | 0.89679 | 0.89755 | 0.97915 | 0.98054 |
|  |  | Withdrawal | -0.05 | 0.87519 | 0.87674 | 0.97659 | 0.97697 |
|  | rs13193545 | No. cigarettes | 0.09 | 0.62896 | 0.62689 | 0.92274 | 0.92348 |
|  |  | ND/craving | -0.06 | 0.90298 | 0.90249 | 0.98061 | 0.98062 |
|  |  | Self-medication | 0.08 | 0.59130 | 0.58896 | 0.90572 | 0.90738 |
|  |  | mFTQ | 0.06 | 0.60883 | 0.60765 | 0.91320 | 0.91336 |
|  |  | ICD-10 | -0.09 | 0.36222 | 0.36016 | 0.84491 | 0.84859 |
|  |  | Withdrawal | -0.35 | 0.22244 | 0.22460 | 0.81692 | 0.82073 |
|  | rs13203628 | No. cigarettes | -0.01 | 0.95159 | 0.95118 | 0.99193 | 0.99216 |
|  |  | ND/craving | -0.32 | 0.51879 | 0.51774 | 0.88888 | 0.89054 |
|  |  | Self-medication | 0.00 | 0.97747 | 0.97734 | 0.99655 | 0.99647 |
|  |  | mFTQ | -0.01 | 0.93881 | 0.93962 | 0.98821 | 0.98861 |
|  |  | ICD-10 | -0.08 | 0.35604 | 0.35921 | 0.84407 | 0.84859 |
|  |  | Withdrawal | -0.31 | 0.24314 | 0.24557 | 0.81692 | 0.82073 |
|  | rs17214592 | No. cigarettes | 0.05 | 0.81611 | 0.81856 | 0.96885 | 0.97009 |
|  |  | ND/craving | 0.36 | 0.51381 | 0.51586 | 0.88888 | 0.89054 |
|  |  | Self-medication | 0.16 | 0.28295 | 0.28579 | 0.81833 | 0.82138 |
|  |  | mFTQ | 0.03 | 0.81082 | 0.81105 | 0.96758 | 0.96843 |
|  |  | ICD-10 | 0.07 | 0.51924 | 0.52095 | 0.88888 | 0.89054 |
|  |  | Withdrawal | 0.20 | 0.50648 | 0.50810 | 0.88805 | 0.89032 |
|  | rs17277929 | No. cigarettes | 0.00 | 0.98241 | 0.98233 | 0.99655 | 0.99647 |
|  |  | ND/craving | -0.69 | 0.24323 | 0.24675 | 0.81692 | 0.82073 |
|  |  | Self-medication | -0.24 | 0.13095 | 0.13410 | 0.78201 | 0.79456 |
|  |  | mFTQ | -0.09 | 0.45529 | 0.45711 | 0.86906 | 0.87224 |
|  |  | ICD-10 | -0.11 | 0.31295 | 0.31667 | 0.82443 | 0.82983 |
|  |  | Withdrawal | -0.15 | 0.64338 | 0.64433 | 0.92403 | 0.92557 |
|  | rs17278409 | No. cigarettes | -0.01 | 0.96073 | 0.96122 | 0.99503 | 0.99540 |
|  |  | ND/craving | -0.36 | 0.48191 | 0.48509 | 0.87147 | 0.87340 |
|  |  | Self-medication | -0.10 | 0.47732 | 0.48313 | 0.87147 | 0.87340 |
|  |  | mFTQ | -0.07 | 0.53973 | 0.54377 | 0.89247 | 0.89469 |
|  |  | ICD-10 | -0.04 | 0.64026 | 0.64126 | 0.92368 | 0.92547 |
|  |  | Withdrawal | -0.11 | 0.70616 | 0.70754 | 0.94554 | 0.94578 |
|  | rs17292684† | No. cigarettes | 0.02 | 0.95793 | 0.95842 | 0.99406 | 0.99481 |
|  |  | ND/craving | 0.34 | 0.71908 | 0.72140 | 0.94944 | 0.95098 |
|  |  | Self-medication | 0.17 | 0.50530 | 0.50678 | 0.88796 | 0.89019 |
|  |  | mFTQ | -0.02 | 0.91886 | 0.92003 | 0.98436 | 0.98444 |
|  |  | ICD-10 | 0.08 | 0.63957 | 0.64121 | 0.92368 | 0.92547 |
|  |  | Withdrawal | -0.17 | 0.74839 | 0.75022 | 0.95566 | 0.95690 |
|  | rs1852629 | No. cigarettes | -0.05 | 0.74925 | 0.75012 | 0.95566 | 0.95690 |
|  |  | ND/craving | -0.10 | 0.81642 | 0.81813 | 0.96885 | 0.97009 |
|  |  | Self-medication | -0.01 | 0.90642 | 0.90528 | 0.98132 | 0.98166 |
|  |  | mFTQ | -0.11 | 0.25709 | 0.25678 | 0.81692 | 0.82073 |
|  |  | ICD-10 | -0.04 | 0.58961 | 0.58921 | 0.90572 | 0.90738 |
|  |  | Withdrawal | -0.09 | 0.70793 | 0.70639 | 0.94554 | 0.94578 |
|  | rs1950005 | No. cigarettes | -0.18 | 0.25138 | 0.25351 | 0.81692 | 0.82073 |
|  |  | ND/craving | -0.57 | 0.20507 | 0.20411 | 0.81692 | 0.82073 |
|  |  | Self-medication | -0.14 | 0.25175 | 0.25317 | 0.81692 | 0.82073 |
|  |  | mFTQ | -0.18 | 0.05060 | 0.05120 | 0.68275 | 0.69122 |
|  |  | ICD-10 | -0.14 | 0.08754 | 0.08773 | 0.71335 | 0.72276 |
|  |  | Withdrawal | -0.16 | 0.51814 | 0.51920 | 0.88888 | 0.89054 |
|  | rs2010884 | No. cigarettes | -0.14 | 0.45748 | 0.45580 | 0.86906 | 0.87224 |
|  |  | ND/craving | -0.25 | 0.63116 | 0.63256 | 0.92368 | 0.92547 |
|  |  | Self-medication | -0.06 | 0.68441 | 0.68348 | 0.93866 | 0.93823 |
|  |  | mFTQ | -0.04 | 0.68896 | 0.69210 | 0.93909 | 0.94137 |
|  |  | ICD-10 | -0.05 | 0.63480 | 0.63554 | 0.92368 | 0.92547 |
|  |  | Withdrawal | -0.22 | 0.44739 | 0.44700 | 0.86722 | 0.86962 |
|  | rs2103277 | No. cigarettes | -0.11 | 0.51091 | 0.51094 | 0.88853 | 0.89041 |
|  |  | ND/craving | -0.59 | 0.20768 | 0.20839 | 0.81692 | 0.82073 |
|  |  | Self-medication | -0.16 | 0.20239 | 0.20632 | 0.81687 | 0.82073 |
|  |  | mFTQ | -0.15 | 0.11622 | 0.11765 | 0.74516 | 0.75032 |
|  |  | ICD-10 | -0.12 | 0.15822 | 0.16002 | 0.81063 | 0.81586 |
|  |  | Withdrawal | -0.15 | 0.54852 | 0.55138 | 0.89247 | 0.89469 |
|  | rs3798683 | No. cigarettes | -0.01 | 0.94164 | 0.94111 | 0.98879 | 0.98886 |
|  |  | ND/craving | 0.39 | 0.48961 | 0.49233 | 0.87638 | 0.88043 |
|  |  | Self-medication | 0.08 | 0.62585 | 0.62867 | 0.92155 | 0.92348 |
|  |  | mFTQ | 0.03 | 0.77525 | 0.77741 | 0.96142 | 0.96227 |
|  |  | ICD-10 | 0.12 | 0.22049 | 0.22115 | 0.81692 | 0.82073 |
|  |  | Withdrawal | 0.44 | 0.14697 | 0.14697 | 0.80189 | 0.80409 |
|  | rs4295492 | No. cigarettes | -0.09 | 0.55812 | 0.55950 | 0.89355 | 0.89469 |
|  |  | ND/craving | -0.30 | 0.48608 | 0.48615 | 0.87147 | 0.87340 |
|  |  | Self-medication | -0.05 | 0.65218 | 0.65385 | 0.92536 | 0.92797 |
|  |  | mFTQ | -0.10 | 0.26819 | 0.26974 | 0.81692 | 0.82073 |
|  |  | ICD-10 | -0.08 | 0.32482 | 0.32703 | 0.82813 | 0.83152 |
|  |  | Withdrawal | -0.14 | 0.55101 | 0.55246 | 0.89247 | 0.89469 |
|  | rs4626436 | No. cigarettes | -0.18 | 0.53969 | 0.54128 | 0.89247 | 0.89469 |
|  |  | ND/craving | 0.01 | 0.98956 | 0.98989 | 0.99655 | 0.99647 |
|  |  | Self-medication | 0.08 | 0.72371 | 0.72484 | 0.94944 | 0.95098 |
|  |  | mFTQ | -0.09 | 0.59889 | 0.60152 | 0.91171 | 0.91247 |
|  |  | ICD-10 | 0.01 | 0.93283 | 0.93225 | 0.98732 | 0.98766 |
|  |  | Withdrawal | -0.34 | 0.45117 | 0.45504 | 0.86906 | 0.87224 |
|  | rs483481 | No. cigarettes | 0.19 | 0.24537 | 0.24596 | 0.81692 | 0.82073 |
|  |  | ND/craving | 0.29 | 0.51938 | 0.52018 | 0.88888 | 0.89054 |
|  |  | Self-medication | 0.16 | 0.20123 | 0.20071 | 0.81687 | 0.82073 |
|  |  | mFTQ | 0.04 | 0.65707 | 0.65693 | 0.92780 | 0.92887 |
|  |  | ICD-10 | 0.01 | 0.93753 | 0.93658 | 0.98779 | 0.98766 |
|  |  | Withdrawal | -0.05 | 0.85319 | 0.85395 | 0.97659 | 0.97697 |
|  | rs4869817 | No. cigarettes | -0.20 | 0.20652 | 0.20770 | 0.81692 | 0.82073 |
|  |  | ND/craving | -0.86 | 0.05168 | 0.05248 | 0.68275 | 0.69122 |
|  |  | Self-medication | -0.14 | 0.24705 | 0.24880 | 0.81692 | 0.82073 |
|  |  | mFTQ | -0.18 | 0.04896 | 0.04954 | 0.68275 | 0.69122 |
|  |  | ICD-10 | -0.16 | 0.05316 | 0.05395 | 0.68275 | 0.69122 |
|  |  | Withdrawal | -0.17 | 0.48312 | 0.48589 | 0.87147 | 0.87340 |
|  | rs4870266 | No. cigarettes | -0.21 | 0.50962 | 0.51175 | 0.88826 | 0.89041 |
|  |  | ND/craving | -1.67 | 0.05460 | 0.05456 | 0.68275 | 0.69122 |
|  |  | Self-medication | -0.40 | 0.09207 | 0.09115 | 0.71653 | 0.72276 |
|  |  | mFTQ | -0.32 | 0.07509 | 0.07421 | 0.70208 | 0.70910 |
|  |  | ICD-10 | -0.34 | 0.03001 | 0.02957 | 0.68275 | 0.69122 |
|  |  | Withdrawal | -0.97 | 0.03867 | 0.03918 | 0.68275 | 0.69122 |
|  | rs510587† | No. cigarettes | 0.09 | 0.79120 | 0.79305 | 0.96758 | 0.96843 |
|  |  | ND/craving | -0.01 | 0.99574 | 0.99588 | 0.99828 | 0.99812 |
|  |  | Self-medication | 0.06 | 0.81744 | 0.81718 | 0.96885 | 0.97009 |
|  |  | mFTQ | 0.22 | 0.28814 | 0.28910 | 0.81833 | 0.82138 |
|  |  | ICD-10 | 0.03 | 0.85751 | 0.85836 | 0.97659 | 0.97697 |
|  |  | Withdrawal | -0.59 | 0.25975 | 0.26063 | 0.81692 | 0.82073 |
|  | rs510769 | No. cigarettes | 0.44 | 0.01835 | 0.01875 | 0.58241 | 0.58246 |
|  |  | ND/craving | 1.67 | 0.00139 | 0.00151 | 0.16678 | 0.18127 |
|  |  | Self-medication | 0.50 | 0.00049 | 0.00070 | 0.12423 | 0.13877 |
|  |  | mFTQ | 0.30 | 0.00626 | 0.00680 | 0.48756 | 0.50408 |
|  |  | ICD-10 | 0.26 | 0.00609 | 0.00642 | 0.48756 | 0.49807 |
|  |  | Withdrawal | 0.95 | 0.00076 | 0.00079 | 0.13255 | 0.13877 |
|  | rs518596 | No. cigarettes | 0.28 | 0.07334 | 0.07516 | 0.70129 | 0.70910 |
|  |  | ND/craving | 0.52 | 0.23703 | 0.23923 | 0.81692 | 0.82073 |
|  |  | Self-medication | 0.17 | 0.16577 | 0.16572 | 0.81132 | 0.81734 |
|  |  | mFTQ | 0.14 | 0.12810 | 0.13042 | 0.77775 | 0.78587 |
|  |  | ICD-10 | 0.04 | 0.61272 | 0.61261 | 0.91370 | 0.91476 |
|  |  | Withdrawal | 0.16 | 0.50470 | 0.50680 | 0.88796 | 0.89019 |
|  | rs557748 | No. cigarettes | 0.64 | 0.00058 | 0.00052 | 0.12423 | 0.12539 |
|  |  | ND/craving | 2.10 | 0.00005 | 0.00007 | 0.05473 | 0.07632 |
|  |  | Self-medication | 0.57 | 0.00006 | 0.00008 | 0.05473 | 0.07632 |
|  |  | mFTQ | 0.35 | 0.00110 | 0.00099 | 0.15836 | 0.15810 |
|  |  | ICD-10 | 0.33 | 0.00050 | 0.00049 | 0.12423 | 0.12539 |
|  |  | Withdrawal | 1.05 | 0.00020 | 0.00025 | 0.12423 | 0.12539 |
|  | rs563649 | No. cigarettes | -0.20 | 0.48331 | 0.48451 | 0.87147 | 0.87340 |
|  |  | ND/craving | -0.70 | 0.38364 | 0.38653 | 0.84797 | 0.85020 |
|  |  | Self-medication | -0.19 | 0.39474 | 0.39841 | 0.84836 | 0.85202 |
|  |  | mFTQ | -0.09 | 0.57520 | 0.57714 | 0.89916 | 0.90267 |
|  |  | ICD-10 | -0.20 | 0.16426 | 0.16399 | 0.81132 | 0.81734 |
|  |  | Withdrawal | -0.33 | 0.44599 | 0.44835 | 0.86722 | 0.87136 |
|  | rs581439 | No. cigarettes | 0.28 | 0.09697 | 0.09617 | 0.71653 | 0.72775 |
|  |  | ND/craving | 0.61 | 0.19688 | 0.19724 | 0.81687 | 0.82073 |
|  |  | Self-medication | 0.16 | 0.19413 | 0.19587 | 0.81687 | 0.82073 |
|  |  | mFTQ | 0.15 | 0.13521 | 0.13593 | 0.79126 | 0.79573 |
|  |  | ICD-10 | 0.06 | 0.49539 | 0.49840 | 0.87955 | 0.88283 |
|  |  | Withdrawal | 0.13 | 0.61040 | 0.61162 | 0.91320 | 0.91476 |
|  | rs590761 | No. cigarettes | 0.63 | 0.02609 | 0.02645 | 0.64414 | 0.66158 |
|  |  | ND/craving | 2.29 | 0.00447 | 0.00437 | 0.43006 | 0.42088 |
|  |  | Self-medication | 0.64 | 0.00369 | 0.00394 | 0.37363 | 0.39941 |
|  |  | mFTQ | 0.33 | 0.04667 | 0.04655 | 0.68275 | 0.69122 |
|  |  | ICD-10 | 0.30 | 0.04179 | 0.04246 | 0.68275 | 0.69122 |
|  |  | Withdrawal | 0.99 | 0.02401 | 0.02352 | 0.62050 | 0.62356 |
|  | rs610231 | No. cigarettes | -0.17 | 0.50755 | 0.50949 | 0.88805 | 0.89041 |
|  |  | ND/craving | -0.55 | 0.44348 | 0.44664 | 0.86364 | 0.86962 |
|  |  | Self-medication | -0.22 | 0.26663 | 0.26954 | 0.81692 | 0.82073 |
|  |  | mFTQ | 0.00 | 0.98983 | 0.98988 | 0.99655 | 0.99647 |
|  |  | ICD-10 | -0.03 | 0.80767 | 0.81077 | 0.96758 | 0.96843 |
|  |  | Withdrawal | -0.29 | 0.45185 | 0.45482 | 0.86906 | 0.87224 |
|  | rs613341 | No. cigarettes | 0.75 | 0.00790 | 0.00804 | 0.52414 | 0.53390 |
|  |  | ND/craving | 2.72 | 0.00067 | 0.00076 | 0.12982 | 0.13877 |
|  |  | Self-medication | 0.70 | 0.00136 | 0.00151 | 0.16678 | 0.18127 |
|  |  | mFTQ | 0.39 | 0.01849 | 0.01839 | 0.58241 | 0.58246 |
|  |  | ICD-10 | 0.38 | 0.00916 | 0.00934 | 0.52414 | 0.53402 |
|  |  | Withdrawal | 1.10 | 0.01180 | 0.01146 | 0.52869 | 0.53402 |
|  | rs633387 | No. cigarettes | 0.08 | 0.64121 | 0.64066 | 0.92368 | 0.92547 |
|  |  | ND/craving | -0.24 | 0.63892 | 0.63967 | 0.92368 | 0.92547 |
|  |  | Self-medication | -0.04 | 0.74884 | 0.74759 | 0.95566 | 0.95608 |
|  |  | mFTQ | 0.03 | 0.75958 | 0.76171 | 0.95749 | 0.95944 |
|  |  | ICD-10 | -0.06 | 0.48362 | 0.48295 | 0.87147 | 0.87340 |
|  |  | Withdrawal | -0.24 | 0.38895 | 0.38610 | 0.84836 | 0.85020 |
|  | rs655059† | No. cigarettes | 0.08 | 0.82069 | 0.81986 | 0.96985 | 0.97009 |
|  |  | ND/craving | -0.06 | 0.95382 | 0.95397 | 0.99193 | 0.99216 |
|  |  | Self-medication | 0.08 | 0.76925 | 0.77037 | 0.96011 | 0.96097 |
|  |  | mFTQ | -0.10 | 0.62492 | 0.62908 | 0.92089 | 0.92348 |
|  |  | ICD-10 | -0.14 | 0.41256 | 0.41550 | 0.85327 | 0.85620 |
|  |  | Withdrawal | -0.32 | 0.53683 | 0.54000 | 0.89247 | 0.89469 |
|  | rs6557337 | No. cigarettes | -0.06 | 0.72658 | 0.72801 | 0.95166 | 0.95174 |
|  |  | ND/craving | 0.11 | 0.80456 | 0.80411 | 0.96758 | 0.96843 |
|  |  | Self-medication | 0.00 | 0.99672 | 0.99657 | 0.99828 | 0.99812 |
|  |  | mFTQ | 0.02 | 0.85588 | 0.85686 | 0.97659 | 0.97697 |
|  |  | ICD-10 | 0.10 | 0.20625 | 0.20877 | 0.81692 | 0.82073 |
|  |  | Withdrawal | 0.12 | 0.61985 | 0.62104 | 0.91735 | 0.91938 |
|  | rs6557339 | No. cigarettes | -0.01 | 0.95784 | 0.95865 | 0.99406 | 0.99481 |
|  |  | ND/craving | -0.31 | 0.52015 | 0.51897 | 0.88888 | 0.89054 |
|  |  | Self-medication | -0.06 | 0.65885 | 0.66071 | 0.92828 | 0.92955 |
|  |  | mFTQ | -0.09 | 0.34444 | 0.34490 | 0.83458 | 0.83774 |
|  |  | ICD-10 | -0.09 | 0.32489 | 0.32456 | 0.82813 | 0.83152 |
|  |  | Withdrawal | -0.07 | 0.80052 | 0.80078 | 0.96758 | 0.96843 |
|  | rs6913456 | No. cigarettes | -0.10 | 0.56326 | 0.56508 | 0.89583 | 0.89872 |
|  |  | ND/craving | -0.26 | 0.56661 | 0.56662 | 0.89822 | 0.89972 |
|  |  | Self-medication | -0.04 | 0.76026 | 0.76164 | 0.95749 | 0.95944 |
|  |  | mFTQ | -0.10 | 0.29176 | 0.29221 | 0.81833 | 0.82138 |
|  |  | ICD-10 | -0.05 | 0.57922 | 0.57996 | 0.90047 | 0.90294 |
|  |  | Withdrawal | 0.02 | 0.93443 | 0.93564 | 0.98732 | 0.98766 |
|  | rs6938037 | No. cigarettes | -0.05 | 0.77441 | 0.77337 | 0.96129 | 0.96222 |
|  |  | ND/craving | -0.19 | 0.68709 | 0.68905 | 0.93866 | 0.94037 |
|  |  | Self-medication | -0.07 | 0.60228 | 0.60391 | 0.91171 | 0.91247 |
|  |  | mFTQ | -0.10 | 0.31871 | 0.31928 | 0.82728 | 0.83152 |
|  |  | ICD-10 | -0.07 | 0.42675 | 0.42600 | 0.85803 | 0.86008 |
|  |  | Withdrawal | -0.04 | 0.87289 | 0.87215 | 0.97659 | 0.97697 |
|  | rs7745095 | No. cigarettes | -0.05 | 0.80966 | 0.81055 | 0.96758 | 0.96843 |
|  |  | ND/craving | 0.39 | 0.52750 | 0.52688 | 0.89197 | 0.89172 |
|  |  | Self-medication | 0.16 | 0.34495 | 0.34538 | 0.83458 | 0.83774 |
|  |  | mFTQ | -0.03 | 0.79167 | 0.79229 | 0.96758 | 0.96843 |
|  |  | ICD-10 | 0.06 | 0.60856 | 0.60844 | 0.91320 | 0.91336 |
|  |  | Withdrawal | 0.08 | 0.81065 | 0.81081 | 0.96758 | 0.96843 |
|  | rs790258 | No. cigarettes | -0.07 | 0.71718 | 0.71384 | 0.94933 | 0.94752 |
|  |  | ND/craving | -0.26 | 0.61454 | 0.61431 | 0.91405 | 0.91505 |
|  |  | Self-medication | -0.12 | 0.41464 | 0.41465 | 0.85340 | 0.85620 |
|  |  | mFTQ | -0.12 | 0.28433 | 0.28621 | 0.81833 | 0.82138 |
|  |  | ICD-10 | -0.10 | 0.31807 | 0.31737 | 0.82728 | 0.83051 |
|  |  | Withdrawal | -0.15 | 0.60932 | 0.61269 | 0.91320 | 0.91476 |
|  | rs9322446 | No. cigarettes | -0.47 | 0.03801 | 0.03850 | 0.68275 | 0.69122 |
|  |  | ND/craving | -0.98 | 0.11069 | 0.11094 | 0.74516 | 0.75032 |
|  |  | Self-medication | -0.27 | 0.10533 | 0.10587 | 0.73616 | 0.74687 |
|  |  | mFTQ | -0.21 | 0.09787 | 0.09832 | 0.71653 | 0.72775 |
|  |  | ICD-10 | -0.17 | 0.12873 | 0.12868 | 0.77775 | 0.78587 |
|  |  | Withdrawal | -0.32 | 0.33922 | 0.34117 | 0.83024 | 0.83599 |
|  | rs9322451 | No. cigarettes | -0.20 | 0.30702 | 0.30615 | 0.82443 | 0.82839 |
|  |  | ND/craving | -0.50 | 0.36579 | 0.36747 | 0.84491 | 0.84876 |
|  |  | Self-medication | -0.06 | 0.66953 | 0.67103 | 0.93275 | 0.93384 |
|  |  | mFTQ | -0.04 | 0.70781 | 0.71087 | 0.94554 | 0.94578 |
|  |  | ICD-10 | -0.08 | 0.40404 | 0.40548 | 0.85202 | 0.85488 |
|  |  | Withdrawal | 0.00 | 0.99152 | 0.99143 | 0.99669 | 0.99660 |
|  | rs9371331 | No. cigarettes | 0.29 | 0.11734 | 0.11887 | 0.74585 | 0.75313 |
|  |  | ND/craving | 1.25 | 0.01719 | 0.01737 | 0.57781 | 0.58246 |
|  |  | Self-medication | 0.29 | 0.04426 | 0.04411 | 0.68275 | 0.69122 |
|  |  | mFTQ | 0.27 | 0.01264 | 0.01324 | 0.53798 | 0.55418 |
|  |  | ICD-10 | 0.24 | 0.01124 | 0.01181 | 0.52414 | 0.53402 |
|  |  | Withdrawal | 0.21 | 0.47083 | 0.47118 | 0.87147 | 0.87340 |
|  | rs9383697 | No. cigarettes | -0.11 | 0.54453 | 0.54480 | 0.89247 | 0.89469 |
|  |  | ND/craving | -0.54 | 0.28356 | 0.28517 | 0.81833 | 0.82138 |
|  |  | Self-medication | -0.17 | 0.21528 | 0.21731 | 0.81692 | 0.82073 |
|  |  | mFTQ | -0.12 | 0.24613 | 0.24618 | 0.81692 | 0.82073 |
|  |  | ICD-10 | -0.13 | 0.14946 | 0.14952 | 0.80364 | 0.80972 |
|  |  | Withdrawal | -0.28 | 0.30584 | 0.31001 | 0.82443 | 0.82927 |
|  | rs9397687 | No. cigarettes | -0.26 | 0.09636 | 0.09778 | 0.71653 | 0.72775 |
|  |  | ND/craving | -0.56 | 0.20294 | 0.20360 | 0.81687 | 0.82073 |
|  |  | Self-medication | -0.12 | 0.30666 | 0.30737 | 0.82443 | 0.82839 |
|  |  | mFTQ | -0.11 | 0.22679 | 0.22828 | 0.81692 | 0.82073 |
|  |  | ICD-10 | -0.07 | 0.37948 | 0.37995 | 0.84491 | 0.84876 |
|  |  | Withdrawal | -0.20 | 0.40102 | 0.40086 | 0.85202 | 0.85368 |
|  | rs9478527 | No. cigarettes | 0.14 | 0.38034 | 0.38297 | 0.84491 | 0.85010 |
|  |  | ND/craving | 0.27 | 0.53546 | 0.53796 | 0.89247 | 0.89469 |
|  |  | Self-medication | 0.03 | 0.76970 | 0.77122 | 0.96011 | 0.96140 |
|  |  | mFTQ | 0.09 | 0.31839 | 0.32044 | 0.82728 | 0.83152 |
|  |  | ICD-10 | 0.05 | 0.55147 | 0.55505 | 0.89247 | 0.89469 |
|  |  | Withdrawal | 0.08 | 0.73701 | 0.73772 | 0.95166 | 0.95343 |
|  | rs9479769 | No. cigarettes | 0.20 | 0.19796 | 0.20020 | 0.81687 | 0.82073 |
|  |  | ND/craving | 0.70 | 0.11429 | 0.11575 | 0.74516 | 0.75032 |
|  |  | Self-medication | 0.14 | 0.24457 | 0.24894 | 0.81692 | 0.82073 |
|  |  | mFTQ | 0.16 | 0.07697 | 0.07772 | 0.71245 | 0.71619 |
|  |  | ICD-10 | 0.16 | 0.05362 | 0.05480 | 0.68275 | 0.69122 |
|  |  | Withdrawal | 0.12 | 0.62111 | 0.62216 | 0.91735 | 0.91973 |
|  | rs9479771† | No. cigarettes | -0.12 | 0.73079 | 0.73332 | 0.95166 | 0.95174 |
|  |  | ND/craving | -0.91 | 0.35683 | 0.36098 | 0.84407 | 0.84859 |
|  |  | Self-medication | -0.01 | 0.97376 | 0.97377 | 0.99655 | 0.99647 |
|  |  | mFTQ | -0.04 | 0.85707 | 0.85668 | 0.97659 | 0.97697 |
|  |  | ICD-10 | -0.08 | 0.63629 | 0.63916 | 0.92368 | 0.92547 |
|  |  | Withdrawal | 0.30 | 0.57778 | 0.58291 | 0.89960 | 0.90467 |
|  | rs9479780 | No. cigarettes | -0.06 | 0.69388 | 0.69579 | 0.94166 | 0.94333 |
|  |  | ND/craving | -0.26 | 0.57002 | 0.57158 | 0.89822 | 0.90267 |
|  |  | Self-medication | -0.08 | 0.50765 | 0.50976 | 0.88805 | 0.89041 |
|  |  | mFTQ | -0.10 | 0.27636 | 0.27884 | 0.81692 | 0.82073 |
|  |  | ICD-10 | -0.09 | 0.27695 | 0.27957 | 0.81692 | 0.82073 |
|  |  | Withdrawal | -0.13 | 0.60981 | 0.61043 | 0.91320 | 0.91476 |
|  | rs9479791 | No. cigarettes | -0.20 | 0.37868 | 0.38123 | 0.84491 | 0.84929 |
|  |  | ND/craving | -0.71 | 0.26069 | 0.26102 | 0.81692 | 0.82073 |
|  |  | Self-medication | -0.08 | 0.63875 | 0.64058 | 0.92368 | 0.92547 |
|  |  | mFTQ | -0.07 | 0.60540 | 0.60534 | 0.91171 | 0.91247 |
|  |  | ICD-10 | -0.10 | 0.36058 | 0.35900 | 0.84491 | 0.84859 |
|  |  | Withdrawal | -0.09 | 0.80125 | 0.80106 | 0.96758 | 0.96843 |
| *SCL6A3* | rs10064525† | No. cigarettes | 0.16 | 0.63250 | 0.63430 | 0.92368 | 0.92547 |
|  |  | ND/craving | 0.10 | 0.91168 | 0.91227 | 0.98436 | 0.98444 |
|  |  | Self-medication | -0.11 | 0.65370 | 0.65440 | 0.92644 | 0.92797 |
|  |  | mFTQ | 0.11 | 0.54958 | 0.55153 | 0.89247 | 0.89469 |
|  |  | ICD-10 | -0.09 | 0.57558 | 0.57882 | 0.89916 | 0.90267 |
|  |  | Withdrawal | -0.31 | 0.52588 | 0.52694 | 0.89080 | 0.89172 |
|  | rs1042098 | No. cigarettes | -0.27 | 0.11222 | 0.11278 | 0.74516 | 0.75032 |
|  |  | ND/craving | -0.72 | 0.13471 | 0.13526 | 0.79100 | 0.79456 |
|  |  | Self-medication | -0.11 | 0.40787 | 0.40880 | 0.85202 | 0.85488 |
|  |  | mFTQ | -0.19 | 0.05510 | 0.05596 | 0.68275 | 0.69122 |
|  |  | ICD-10 | -0.10 | 0.23166 | 0.23222 | 0.81692 | 0.82073 |
|  |  | Withdrawal | -0.26 | 0.30848 | 0.30828 | 0.82443 | 0.82839 |
|  | rs1048953 | No. cigarettes | 0.22 | 0.16623 | 0.16873 | 0.81132 | 0.81734 |
|  |  | ND/craving | 0.93 | 0.04011 | 0.04074 | 0.68275 | 0.69122 |
|  |  | Self-medication | 0.31 | 0.01143 | 0.01156 | 0.52414 | 0.53402 |
|  |  | mFTQ | 0.19 | 0.04501 | 0.04615 | 0.68275 | 0.69122 |
|  |  | ICD-10 | 0.16 | 0.05309 | 0.05386 | 0.68275 | 0.69122 |
|  |  | Withdrawal | 0.59 | 0.01622 | 0.01647 | 0.57781 | 0.58246 |
|  | rs11564757† | No. cigarettes | -0.29 | 0.41508 | 0.41958 | 0.85340 | 0.85695 |
|  |  | ND/craving | -0.70 | 0.48512 | 0.48757 | 0.87147 | 0.87435 |
|  |  | Self-medication | -0.38 | 0.16917 | 0.16816 | 0.81132 | 0.81734 |
|  |  | mFTQ | -0.02 | 0.93926 | 0.93984 | 0.98821 | 0.98861 |
|  |  | ICD-10 | -0.19 | 0.30434 | 0.30526 | 0.82443 | 0.82839 |
|  |  | Withdrawal | -0.99 | 0.06943 | 0.06844 | 0.68903 | 0.69122 |
|  | rs11564772 | No. cigarettes | -0.37 | 0.24765 | 0.24849 | 0.81692 | 0.82073 |
|  |  | ND/craving | -0.91 | 0.30338 | 0.30335 | 0.82443 | 0.82839 |
|  |  | Self-medication | -0.05 | 0.84421 | 0.84511 | 0.97554 | 0.97638 |
|  |  | mFTQ | -0.14 | 0.45419 | 0.45410 | 0.86906 | 0.87224 |
|  |  | ICD-10 | -0.14 | 0.36960 | 0.36901 | 0.84491 | 0.84876 |
|  |  | Withdrawal | -0.48 | 0.32189 | 0.32291 | 0.82813 | 0.83152 |
|  | rs11737901 | No. cigarettes | 0.16 | 0.35927 | 0.35957 | 0.84491 | 0.84859 |
|  |  | ND/craving | 0.85 | 0.07770 | 0.07914 | 0.71245 | 0.72276 |
|  |  | Self-medication | 0.34 | 0.01015 | 0.01028 | 0.52414 | 0.53402 |
|  |  | mFTQ | 0.18 | 0.06738 | 0.06910 | 0.68275 | 0.69122 |
|  |  | ICD-10 | 0.18 | 0.04080 | 0.04184 | 0.68275 | 0.69122 |
|  |  | Withdrawal | 0.68 | 0.00888 | 0.00882 | 0.52414 | 0.53402 |
|  | rs12516758 | No. cigarettes | 0.19 | 0.34249 | 0.34572 | 0.83458 | 0.83774 |
|  |  | ND/craving | 0.88 | 0.12397 | 0.12477 | 0.76898 | 0.77377 |
|  |  | Self-medication | 0.28 | 0.07302 | 0.07482 | 0.70129 | 0.70910 |
|  |  | mFTQ | 0.17 | 0.14662 | 0.14779 | 0.80189 | 0.80409 |
|  |  | ICD-10 | 0.13 | 0.22125 | 0.22279 | 0.81692 | 0.82073 |
|  |  | Withdrawal | 0.44 | 0.15996 | 0.16133 | 0.81074 | 0.81586 |
|  | rs12652860 | No. cigarettes | -0.07 | 0.68487 | 0.68553 | 0.93866 | 0.93907 |
|  |  | ND/craving | -0.39 | 0.43511 | 0.43551 | 0.85904 | 0.86375 |
|  |  | Self-medication | -0.10 | 0.45499 | 0.45670 | 0.86906 | 0.87224 |
|  |  | mFTQ | -0.07 | 0.52515 | 0.52357 | 0.89035 | 0.89054 |
|  |  | ICD-10 | -0.08 | 0.38979 | 0.39280 | 0.84836 | 0.85202 |
|  |  | Withdrawal | -0.16 | 0.54860 | 0.54836 | 0.89247 | 0.89469 |
|  | rs2042449 | No. cigarettes | -0.03 | 0.88988 | 0.89005 | 0.97659 | 0.97697 |
|  |  | ND/craving | -0.38 | 0.46603 | 0.46688 | 0.87147 | 0.87340 |
|  |  | Self-medication | -0.09 | 0.52152 | 0.52277 | 0.88888 | 0.89054 |
|  |  | mFTQ | -0.08 | 0.45993 | 0.46148 | 0.86906 | 0.87224 |
|  |  | ICD-10 | -0.03 | 0.76168 | 0.76663 | 0.95755 | 0.95944 |
|  |  | Withdrawal | -0.17 | 0.54127 | 0.54370 | 0.89247 | 0.89469 |
|  | rs2455391 | No. cigarettes | 0.15 | 0.41214 | 0.41497 | 0.85327 | 0.85620 |
|  |  | ND/craving | 0.43 | 0.38927 | 0.38898 | 0.84836 | 0.85142 |
|  |  | Self-medication | 0.18 | 0.19626 | 0.19791 | 0.81687 | 0.82073 |
|  |  | mFTQ | 0.06 | 0.53656 | 0.53837 | 0.89247 | 0.89469 |
|  |  | ICD-10 | 0.11 | 0.21283 | 0.21539 | 0.81692 | 0.82073 |
|  |  | Withdrawal | 0.13 | 0.64120 | 0.64464 | 0.92368 | 0.92557 |
|  | rs250681 | No. cigarettes | -0.10 | 0.55502 | 0.55548 | 0.89350 | 0.89469 |
|  |  | ND/craving | -0.64 | 0.16184 | 0.16219 | 0.81132 | 0.81586 |
|  |  | Self-medication | -0.29 | 0.01897 | 0.01866 | 0.58241 | 0.58246 |
|  |  | mFTQ | -0.11 | 0.26728 | 0.27004 | 0.81692 | 0.82073 |
|  |  | ICD-10 | -0.13 | 0.12776 | 0.12897 | 0.77775 | 0.78587 |
|  |  | Withdrawal | -0.57 | 0.02212 | 0.02174 | 0.61858 | 0.61765 |
|  | rs2550948 | No. cigarettes | -0.14 | 0.40561 | 0.40541 | 0.85202 | 0.85488 |
|  |  | ND/craving | -0.55 | 0.23942 | 0.24074 | 0.81692 | 0.82073 |
|  |  | Self-medication | -0.16 | 0.21406 | 0.21382 | 0.81692 | 0.82073 |
|  |  | mFTQ | -0.13 | 0.16979 | 0.17132 | 0.81132 | 0.81734 |
|  |  | ICD-10 | -0.10 | 0.23351 | 0.23524 | 0.81692 | 0.82073 |
|  |  | Withdrawal | -0.20 | 0.43411 | 0.43527 | 0.85904 | 0.86375 |
|  | rs2617605 | No. cigarettes | 0.12 | 0.45833 | 0.45993 | 0.86906 | 0.87224 |
|  |  | ND/craving | 0.46 | 0.32279 | 0.32654 | 0.82813 | 0.83152 |
|  |  | Self-medication | 0.14 | 0.25415 | 0.25606 | 0.81692 | 0.82073 |
|  |  | mFTQ | 0.10 | 0.28410 | 0.28705 | 0.81833 | 0.82138 |
|  |  | ICD-10 | 0.12 | 0.14351 | 0.14416 | 0.79921 | 0.80409 |
|  |  | Withdrawal | 0.00 | 0.99243 | 0.99260 | 0.99709 | 0.99726 |
|  | rs27048 | No. cigarettes | 0.05 | 0.76316 | 0.76467 | 0.95755 | 0.95944 |
|  |  | ND/craving | 0.03 | 0.93990 | 0.94052 | 0.98821 | 0.98878 |
|  |  | Self-medication | 0.00 | 0.99514 | 0.99519 | 0.99825 | 0.99812 |
|  |  | mFTQ | -0.01 | 0.91759 | 0.91824 | 0.98436 | 0.98444 |
|  |  | ICD-10 | 0.01 | 0.86973 | 0.87269 | 0.97659 | 0.97697 |
|  |  | Withdrawal | -0.06 | 0.79447 | 0.79621 | 0.96758 | 0.96843 |
|  | rs27072 | No. cigarettes | 0.18 | 0.36806 | 0.36900 | 0.84491 | 0.84876 |
|  |  | ND/craving | 0.33 | 0.56077 | 0.56187 | 0.89365 | 0.89582 |
|  |  | Self-medication | -0.03 | 0.85342 | 0.85499 | 0.97659 | 0.97697 |
|  |  | mFTQ | 0.05 | 0.63990 | 0.64149 | 0.92368 | 0.92547 |
|  |  | ICD-10 | 0.08 | 0.43082 | 0.43296 | 0.85904 | 0.86375 |
|  |  | Withdrawal | 0.15 | 0.63361 | 0.63474 | 0.92368 | 0.92547 |
|  | rs27074 | No. cigarettes | -0.40 | 0.11684 | 0.11756 | 0.74516 | 0.75032 |
|  |  | ND/craving | -0.83 | 0.24080 | 0.24374 | 0.81692 | 0.82073 |
|  |  | Self-medication | -0.24 | 0.22766 | 0.22957 | 0.81692 | 0.82073 |
|  |  | mFTQ | -0.17 | 0.25847 | 0.26284 | 0.81692 | 0.82073 |
|  |  | ICD-10 | -0.06 | 0.64413 | 0.64427 | 0.92403 | 0.92557 |
|  |  | Withdrawal | -0.23 | 0.55196 | 0.55480 | 0.89247 | 0.89469 |
|  | rs3756450 | No. cigarettes | -0.05 | 0.80726 | 0.81007 | 0.96758 | 0.96843 |
|  |  | ND/craving | -0.29 | 0.63784 | 0.63816 | 0.92368 | 0.92547 |
|  |  | Self-medication | -0.15 | 0.37686 | 0.38010 | 0.84491 | 0.84876 |
|  |  | mFTQ | -0.08 | 0.54575 | 0.54558 | 0.89247 | 0.89469 |
|  |  | ICD-10 | -0.07 | 0.55502 | 0.55876 | 0.89350 | 0.89469 |
|  |  | Withdrawal | -0.13 | 0.69972 | 0.70322 | 0.94176 | 0.94515 |
|  | rs3776511 | No. cigarettes | -0.06 | 0.77018 | 0.77027 | 0.96011 | 0.96097 |
|  |  | ND/craving | 0.07 | 0.89539 | 0.89597 | 0.97915 | 0.98018 |
|  |  | Self-medication | 0.06 | 0.69036 | 0.69039 | 0.93977 | 0.94037 |
|  |  | mFTQ | -0.02 | 0.83444 | 0.83475 | 0.97343 | 0.97379 |
|  |  | ICD-10 | 0.03 | 0.78094 | 0.78321 | 0.96417 | 0.96540 |
|  |  | Withdrawal | -0.02 | 0.95215 | 0.95353 | 0.99193 | 0.99216 |
|  | rs40184 | No. cigarettes | -0.01 | 0.93695 | 0.93752 | 0.98772 | 0.98778 |
|  |  | ND/craving | -0.11 | 0.81077 | 0.81212 | 0.96758 | 0.96905 |
|  |  | Self-medication | 0.02 | 0.88620 | 0.88649 | 0.97659 | 0.97697 |
|  |  | mFTQ | -0.07 | 0.46956 | 0.47086 | 0.87147 | 0.87340 |
|  |  | ICD-10 | 0.03 | 0.73021 | 0.73180 | 0.95166 | 0.95174 |
|  |  | Withdrawal | -0.08 | 0.74548 | 0.74592 | 0.95528 | 0.95552 |
|  | rs40358 | No. cigarettes | -0.24 | 0.30530 | 0.30834 | 0.82443 | 0.82839 |
|  |  | ND/craving | -0.85 | 0.19627 | 0.19680 | 0.81687 | 0.82073 |
|  |  | Self-medication | -0.12 | 0.49604 | 0.49738 | 0.87960 | 0.88283 |
|  |  | mFTQ | -0.18 | 0.18194 | 0.18385 | 0.81687 | 0.82073 |
|  |  | ICD-10 | -0.16 | 0.18885 | 0.18948 | 0.81687 | 0.82073 |
|  |  | Withdrawal | -0.27 | 0.46054 | 0.46017 | 0.86906 | 0.87224 |
|  | rs456082 | No. cigarettes | -0.03 | 0.87069 | 0.87300 | 0.97659 | 0.97697 |
|  |  | ND/craving | -0.14 | 0.79197 | 0.79445 | 0.96758 | 0.96843 |
|  |  | Self-medication | -0.25 | 0.08449 | 0.08466 | 0.71335 | 0.72276 |
|  |  | mFTQ | 0.02 | 0.88228 | 0.88411 | 0.97659 | 0.97697 |
|  |  | ICD-10 | -0.07 | 0.48614 | 0.48822 | 0.87147 | 0.87472 |
|  |  | Withdrawal | -0.39 | 0.16458 | 0.16675 | 0.81132 | 0.81734 |
|  | rs6347 | No. cigarettes | -0.14 | 0.40343 | 0.40837 | 0.85202 | 0.85488 |
|  |  | ND/craving | -0.32 | 0.48259 | 0.48290 | 0.87147 | 0.87340 |
|  |  | Self-medication | 0.00 | 0.98571 | 0.98595 | 0.99655 | 0.99647 |
|  |  | mFTQ | -0.08 | 0.40700 | 0.40751 | 0.85202 | 0.85488 |
|  |  | ICD-10 | -0.03 | 0.68554 | 0.68512 | 0.93866 | 0.93907 |
|  |  | Withdrawal | -0.27 | 0.28540 | 0.28665 | 0.81833 | 0.82138 |
|  | rs6876225 | No. cigarettes | -0.29 | 0.38626 | 0.38834 | 0.84836 | 0.85142 |
|  |  | ND/craving | -1.21 | 0.19913 | 0.20001 | 0.81687 | 0.82073 |
|  |  | Self-medication | -0.20 | 0.42844 | 0.43070 | 0.85870 | 0.86205 |
|  |  | mFTQ | -0.19 | 0.32971 | 0.33225 | 0.82975 | 0.83365 |
|  |  | ICD-10 | -0.25 | 0.13915 | 0.13897 | 0.79750 | 0.79829 |
|  |  | Withdrawal | -0.61 | 0.23001 | 0.23075 | 0.81692 | 0.82073 |
| *SCL6A4* | rs11080121 | No. cigarettes | -0.07 | 0.65045 | 0.65148 | 0.92516 | 0.92773 |
|  |  | ND/craving | -0.49 | 0.25570 | 0.25559 | 0.81692 | 0.82073 |
|  |  | Self-medication | -0.14 | 0.23347 | 0.23516 | 0.81692 | 0.82073 |
|  |  | mFTQ | -0.06 | 0.49452 | 0.49411 | 0.87945 | 0.88117 |
|  |  | ICD-10 | -0.10 | 0.18827 | 0.18865 | 0.81687 | 0.82073 |
|  |  | Withdrawal | -0.06 | 0.79337 | 0.79495 | 0.96758 | 0.96843 |
|  | rs140700 | No. cigarettes | 0.33 | 0.27124 | 0.27643 | 0.81692 | 0.82073 |
|  |  | ND/craving | 0.59 | 0.47487 | 0.47695 | 0.87147 | 0.87340 |
|  |  | Self-medication | 0.37 | 0.09643 | 0.09690 | 0.71653 | 0.72775 |
|  |  | mFTQ | 0.14 | 0.39638 | 0.39848 | 0.84836 | 0.85202 |
|  |  | ICD-10 | 0.11 | 0.46049 | 0.46276 | 0.86906 | 0.87294 |
|  |  | Withdrawal | 0.43 | 0.33068 | 0.33365 | 0.82975 | 0.83365 |
|  | rs2020942 | No. cigarettes | 0.20 | 0.23357 | 0.23674 | 0.81692 | 0.82073 |
|  |  | ND/craving | 0.33 | 0.46953 | 0.46795 | 0.87147 | 0.87340 |
|  |  | Self-medication | 0.02 | 0.85719 | 0.85645 | 0.97659 | 0.97697 |
|  |  | mFTQ | 0.01 | 0.88519 | 0.88603 | 0.97659 | 0.97697 |
|  |  | ICD-10 | 0.05 | 0.51828 | 0.51696 | 0.88888 | 0.89054 |
|  |  | Withdrawal | -0.20 | 0.41290 | 0.41231 | 0.85327 | 0.85586 |
|  | rs25528 | No. cigarettes | 0.02 | 0.92982 | 0.93003 | 0.98723 | 0.98745 |
|  |  | ND/craving | 0.50 | 0.36893 | 0.37104 | 0.84491 | 0.84876 |
|  |  | Self-medication | 0.19 | 0.20342 | 0.20542 | 0.81687 | 0.82073 |
|  |  | mFTQ | 0.11 | 0.32849 | 0.33064 | 0.82975 | 0.83365 |
|  |  | ICD-10 | 0.10 | 0.33535 | 0.33713 | 0.82975 | 0.83365 |
|  |  | Withdrawal | 0.55 | 0.06709 | 0.06749 | 0.68275 | 0.69122 |
|  | rs4251417 | No. cigarettes | 0.10 | 0.69972 | 0.70237 | 0.94176 | 0.94515 |
|  |  | ND/craving | -0.01 | 0.98914 | 0.99000 | 0.99655 | 0.99647 |
|  |  | Self-medication | 0.00 | 0.98889 | 0.98936 | 0.99655 | 0.99647 |
|  |  | mFTQ | -0.03 | 0.85516 | 0.85474 | 0.97659 | 0.97697 |
|  |  | ICD-10 | -0.06 | 0.64576 | 0.64538 | 0.92403 | 0.92557 |
|  |  | Withdrawal | 0.39 | 0.30137 | 0.29974 | 0.82443 | 0.82826 |
|  | rs7214991 | No. cigarettes | 0.14 | 0.38130 | 0.38025 | 0.84553 | 0.84876 |
|  |  | ND/craving | 0.55 | 0.19837 | 0.20072 | 0.81687 | 0.82073 |
|  |  | Self-medication | 0.12 | 0.29000 | 0.29034 | 0.81833 | 0.82138 |
|  |  | mFTQ | 0.09 | 0.29751 | 0.29654 | 0.82328 | 0.82308 |
|  |  | ICD-10 | 0.12 | 0.14355 | 0.14357 | 0.79921 | 0.80409 |
|  |  | Withdrawal | 0.26 | 0.26924 | 0.26919 | 0.81692 | 0.82073 |
| *TH* | rs10743149 | No. cigarettes | -0.26 | 0.26738 | 0.26853 | 0.81692 | 0.82073 |
|  |  | ND/craving | -0.22 | 0.73645 | 0.73666 | 0.95166 | 0.95343 |
|  |  | Self-medication | 0.05 | 0.78217 | 0.78345 | 0.96445 | 0.96540 |
|  |  | mFTQ | -0.13 | 0.33968 | 0.34300 | 0.83024 | 0.83774 |
|  |  | ICD-10 | 0.01 | 0.94419 | 0.94392 | 0.98902 | 0.98910 |
|  |  | Withdrawal | -0.31 | 0.38705 | 0.38655 | 0.84836 | 0.85020 |
|  | rs10840447 | No. cigarettes | 0.05 | 0.75768 | 0.75803 | 0.95749 | 0.95912 |
|  |  | ND/craving | -0.13 | 0.76707 | 0.76753 | 0.95851 | 0.95961 |
|  |  | Self-medication | -0.05 | 0.69839 | 0.69949 | 0.94166 | 0.94333 |
|  |  | mFTQ | -0.03 | 0.77357 | 0.77439 | 0.96129 | 0.96227 |
|  |  | ICD-10 | 0.00 | 0.98246 | 0.98268 | 0.99655 | 0.99647 |
|  |  | Withdrawal | 0.09 | 0.71293 | 0.71110 | 0.94741 | 0.94578 |
|  | rs10840491 | No. cigarettes | 0.11 | 0.66589 | 0.66959 | 0.93268 | 0.93384 |
|  |  | ND/craving | -0.02 | 0.98140 | 0.98250 | 0.99655 | 0.99647 |
|  |  | Self-medication | -0.04 | 0.83882 | 0.83901 | 0.97441 | 0.97550 |
|  |  | mFTQ | -0.03 | 0.84253 | 0.84453 | 0.97554 | 0.97638 |
|  |  | ICD-10 | 0.08 | 0.50428 | 0.50703 | 0.88796 | 0.89019 |
|  |  | Withdrawal | 0.07 | 0.85433 | 0.85703 | 0.97659 | 0.97697 |
|  | rs11564709† | No. cigarettes | -0.01 | 0.96636 | 0.96707 | 0.99583 | 0.99647 |
|  |  | ND/craving | 0.66 | 0.44330 | 0.44394 | 0.86364 | 0.86705 |
|  |  | Self-medication | -0.14 | 0.55701 | 0.56112 | 0.89350 | 0.89577 |
|  |  | mFTQ | 0.01 | 0.95629 | 0.95639 | 0.99397 | 0.99406 |
|  |  | ICD-10 | -0.02 | 0.91833 | 0.91916 | 0.98436 | 0.98444 |
|  |  | Withdrawal | 0.33 | 0.47946 | 0.48293 | 0.87147 | 0.87340 |
|  | rs2070762 | No. cigarettes | -0.03 | 0.86150 | 0.86245 | 0.97659 | 0.97697 |
|  |  | ND/craving | -0.46 | 0.28431 | 0.28440 | 0.81833 | 0.82138 |
|  |  | Self-medication | -0.09 | 0.46475 | 0.46552 | 0.87147 | 0.87340 |
|  |  | mFTQ | -0.06 | 0.50097 | 0.50148 | 0.88601 | 0.88692 |
|  |  | ICD-10 | -0.03 | 0.70586 | 0.70710 | 0.94554 | 0.94578 |
|  |  | Withdrawal | -0.12 | 0.61956 | 0.61804 | 0.91735 | 0.91776 |
|  | rs3842727 | No. cigarettes | -0.06 | 0.71474 | 0.71586 | 0.94741 | 0.94955 |
|  |  | ND/craving | 0.28 | 0.55235 | 0.55160 | 0.89247 | 0.89469 |
|  |  | Self-medication | 0.01 | 0.91533 | 0.91724 | 0.98436 | 0.98444 |
|  |  | mFTQ | -0.02 | 0.80949 | 0.80954 | 0.96758 | 0.96843 |
|  |  | ICD-10 | 0.04 | 0.67899 | 0.67657 | 0.93410 | 0.93426 |
|  |  | Withdrawal | 0.07 | 0.78466 | 0.78551 | 0.96628 | 0.96713 |
|  | rs3842752 | No. cigarettes | 0.09 | 0.62735 | 0.63047 | 0.92235 | 0.92482 |
|  |  | ND/craving | 0.73 | 0.17815 | 0.18065 | 0.81687 | 0.82073 |
|  |  | Self-medication | 0.06 | 0.66561 | 0.66609 | 0.93268 | 0.93384 |
|  |  | mFTQ | 0.09 | 0.42846 | 0.42916 | 0.85870 | 0.86099 |
|  |  | ICD-10 | 0.07 | 0.49734 | 0.49795 | 0.88039 | 0.88283 |
|  |  | Withdrawal | 0.23 | 0.44107 | 0.44091 | 0.86332 | 0.86652 |
|  | rs4930046 | No. cigarettes | 0.05 | 0.76256 | 0.76465 | 0.95755 | 0.95944 |
|  |  | ND/craving | 0.13 | 0.77462 | 0.77662 | 0.96129 | 0.96227 |
|  |  | Self-medication | 0.15 | 0.21273 | 0.21616 | 0.81692 | 0.82073 |
|  |  | mFTQ | 0.10 | 0.29459 | 0.29658 | 0.81833 | 0.82308 |
|  |  | ICD-10 | 0.04 | 0.66130 | 0.66406 | 0.93018 | 0.93263 |
|  |  | Withdrawal | 0.23 | 0.33045 | 0.33297 | 0.82975 | 0.83365 |
|  | rs6356 | No. cigarettes | 0.01 | 0.96265 | 0.96195 | 0.99503 | 0.99540 |
|  |  | ND/craving | 0.27 | 0.55617 | 0.55779 | 0.89350 | 0.89469 |
|  |  | Self-medication | 0.18 | 0.14242 | 0.14421 | 0.79921 | 0.80409 |
|  |  | mFTQ | 0.07 | 0.43587 | 0.43691 | 0.85904 | 0.86375 |
|  |  | ICD-10 | 0.08 | 0.33800 | 0.34041 | 0.83024 | 0.83520 |
|  |  | Withdrawal | 0.26 | 0.29602 | 0.29925 | 0.82035 | 0.82810 |
|  | rs6578993 | No. cigarettes | 0.21 | 0.34519 | 0.34810 | 0.83458 | 0.83895 |
|  |  | ND/craving | 0.15 | 0.80228 | 0.80213 | 0.96758 | 0.96843 |
|  |  | Self-medication | 0.00 | 0.98183 | 0.98291 | 0.99655 | 0.99647 |
|  |  | mFTQ | 0.03 | 0.83815 | 0.83881 | 0.97441 | 0.97550 |
|  |  | ICD-10 | 0.07 | 0.53168 | 0.53448 | 0.89247 | 0.89469 |
|  |  | Withdrawal | 0.23 | 0.49136 | 0.49613 | 0.87733 | 0.88150 |
|  | rs7119275 | No. cigarettes | 0.00 | 0.99829 | 0.99805 | 0.99933 | 0.99909 |
|  |  | ND/craving | 0.03 | 0.94588 | 0.94635 | 0.98902 | 0.98951 |
|  |  | Self-medication | -0.10 | 0.41922 | 0.42315 | 0.85531 | 0.86008 |
|  |  | mFTQ | -0.04 | 0.65049 | 0.65411 | 0.92516 | 0.92797 |
|  |  | ICD-10 | -0.05 | 0.57177 | 0.57695 | 0.89822 | 0.90267 |
|  |  | Withdrawal | -0.22 | 0.37844 | 0.38031 | 0.84491 | 0.84876 |

*Based on 88,326 permutations

FDR = false discovery rate

† SNP recoded in a dominant model (rare homozygote recoded as heterozygote)

**Table S4**. Correlation Coefficients Between Number of Cigarettes Smoked and Nicotine Dependence Phenotypes. NDIT Study (n=544), 1999-2008

|  | ND/Craving | ICD-10 | mFTQ | No. cigarettes  smoked | Self-  Medication |
| --- | --- | --- | --- | --- | --- |
| ND/Craving | - | - | - | - | - |
| ICD-10 | 0.83 | - | - | - | - |
| mFTQ | 0.82 | 0.68 | - | - | - |
| No. cigarettes smoked | 0.63 | 0.54 | 0.72 | - | - |
| Self-medication | 0.73 | 0.70 | 0.61 | 0.51 | - |
| Withdrawal | 0.71 | 0.72 | 0.59 | 0.47 | 0.67 |

**Table S5**. Linkage Disequilibrium Correlation Coefficients for SNPs

in Genes *COMT, DDC, DRD2/ANKK1* and *OPRM1* that Were Statistically

Significantly Associated with the Number of Cigarettes Smoked and/or

Nicotine Dependence Phenotypes. NDIT Study, 1999-2008.

| *COMT* | rs174696 | rs2020917 | rs887200 |
| --- | --- | --- | --- |
| rs2020917 | 0.08 | - | - |
| rs887200 | 0.20 | 0.01 | - |
| rs8140265 | 0.07 | 0.98 | 0.01 |

| *DDC* | rs921451 |
| --- | --- |
| rs4947644 | 0.55 |

| *DRD2/ANKK1* | rs4936270 | rs4586205 | rs6276 |
| --- | --- | --- | --- |
| rs4586205 | 0.18 | - | - |
| rs6276 | 0.16 | 0.69 | - |
| rs2242592 | 0.14 | 0.68 | 0.98 |

| *OPRM1* | rs510769 | rs557748 | rs590761 |
| --- | --- | --- | --- |
| rs557748 | 0.74 | - | - |
| rs590761 | 0.18 | 0.23 | - |
| rs613341 | 0.18 | 0.25 | 0.94 |
